# Genus *Myricaria*, the Smaller Sister of Tamarisks—Ornamental Value, Phytochemistry, Biological Activities and Traditional Uses

**DOI:** 10.3390/life16050832

**Published:** 2026-05-19

**Authors:** Justyna Makowska-Wąs, Danuta Sobolewska, Karolina Grabowska, Dagmara Wróbel-Biedrawa, Irma Podolak

**Affiliations:** Department of Pharmacognosy, Medical College, Jagiellonian University, 30-688 Kraków, Poland; justyna.makowska-was@uj.edu.pl (J.M.-W.); danuta.sobolewska@uj.edu.pl (D.S.); karolina1.grabowska@uj.edu.pl (K.G.); dagmara.wrobel-biedrawa@uj.edu.pl (D.W.-B.)

**Keywords:** *Myricaria*, phytochemistry, ornamental, sulfated flavonoids, tannins, triterpenes, alkanediols

## Abstract

The genus *Myricaria* is one of the four genera within the Tamaricaceae family. It comprises 13 species distributed across Eurasia. Phytochemical studies carried out on *Myricaria* plants revealed the presence of flavonoids (including rare, sulfated derivatives), tannins, phenolic acid derivatives, triterpenoids, steroids, and alkanediols. Studies on the extracts and compounds isolated from the described to date have demonstrated various biological activities, including antioxidant, anti-inflammatory, cytotoxic, antimicrobial, analgesic, antinociceptive, cholinergic, and glucose absorption reducing properties. This work provides a comprehensive overview of the botanical and detailed phytochemical characteristics, ornamental value, pharmacological properties, and traditional uses of the *Myricaria* genus representatives. The article fills a longstanding gap in the literature as no other integrative description is currently available.

## 1. Introduction

The genus *Myricaria* Desv. comprises 13 species and is a representative of family Tamaricaceae (the tamarisk family), which also includes three other genera: *Tamarix* L. (73 species), *Reaumuria* L. (25 species), and *Myrtama* Ovcz. & Kinzik (1 species) [1]. The tamarisk family includes shrubs and trees that are adapted to different water and soil conditions. They are usually found in xeric saline areas and are classified as halophytes (plants adapted to life in saline soil), rheophytes (flood-tolerant plants that are confined to the beds of swift-running streams and rivers), or xerophytes (plants adapted to dry habitats) [2,3,4]. The genus *Myricaria* comprises stress-tolerant, montane to alpine species that, atypically for the tamarisk family, usually occur in mesic and non-saline habitats [3]. Their common feature is a preference to grow along rivers and streams.

Noteworthy, however, members of the *Myricaria* genus are characterized by a high tolerance to a wide range of environmental stressors and a remarkable ability to adapt to diverse habitats. These plants can efficiently colonize undisturbed areas, often serve as effective pioneer plants in post-industrial landscapes, and are employed in the stabilization of riverbanks. Because of these characteristics—combined with the striking appearance of their distinctive flowering shoots—they have become valued as ornamental plants. Similarly to their close relatives, the tamarisks (*Tamarix* spp.), they can form dense, visually striking forms in both traditional and modern garden compositions, cover large areas, and create a structural background for other plant species.

Beyond their ornamental value, representatives of *Myricaria* are also considered highly important due to their medicinal uses, as well as their broader non-medical applications, especially ecological uses like support for biodiversity or habitat restoration, riverbank stabilization, and erosion control. Recent pharmacological and phytochemical studies have provided novel data referring to this interesting but lesser-known genus within the Tamaricaceae family.

To the best of our knowledge, the existing scientific literature lacks a comprehensive synthesis of this genus. Therefore, the aim of this work is to provide an integrative overview of *Myricaria* species, summarizing current knowledge on biology, phytochemistry, ethnobotany, and pharmacology, highlighting its ornamental value as well as medicinal significance.

## 2. Methods

This review used a structured literature search strategy, with English as the preferred language. Relevant information on the genus *Myricaria* Desv. was gathered from various electronic databases, including Scopus, PubMed, and other specialized sources (such as Google Scholar), and included publications available up to the end of February 2026. No strict date limits were applied. As keywords, the names of the genus and species, along with their synonyms listed in the Plants of the World Online database (POWO) [1], were used (see also Table 1). Therefore, the representative search basic keywords were, e.g., “Myricaria”, “Myricaria bracteata”, “M. bracteata”, “Myricaria alopecuroides”, or “Tamaricaceae”. Articles concerning *Myricaria elegans* Royle were excluded, as this name currently corresponds to a synonym of a species within a different genus—*Myrtama* Ovcz. & Kinzik.

Traditional-use reports, phytochemical investigations, and pharmacological studies were assessed using the same inclusion criteria, based on their direct relevance to plant chemical composition, biological activity, or mechanistic interpretation. Studies focusing exclusively on ecological or biogeographical aspects were included only if they provided insights relevant to the topics discussed. Additional relevant publications were identified through manual screening of the reference lists of retrieved articles. Duplicate records were removed during this process.

From the collected materials, articles with imprecise English abstracts or without access to the full texts were also excluded. Original research articles and reviews published in Chinese or Russian were considered due to the geographic distribution of *Myricaria* species. Such studies were included only when full texts were available, and methodological details could be verified. However, due to the large number of studies published predominantly in Chinese-language journals, abstracts containing sufficiently detailed information were included in the review, with appropriate annotation.

**Figure 1:**
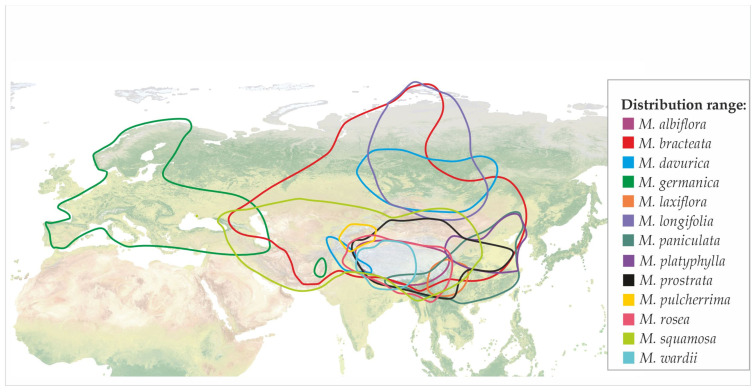
Distribution of *Myricaria* spp. in Europe and Asia (based on NaturalEarth ^CC 0^ Map View of Eurasia) [1,28].

**Figure 2:**
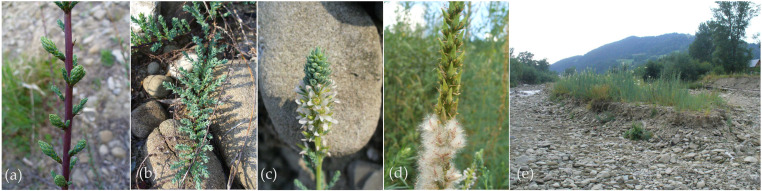
Morphology of *Myricaria germanica*: (**a**,**b**) shoots with young leaves; (**c**) inflorescence; (**d**) fruits and seeds; (**e**) typical habitat, Carpathians in South Poland (J. Makowska-Wąs).

**Figure 3:**
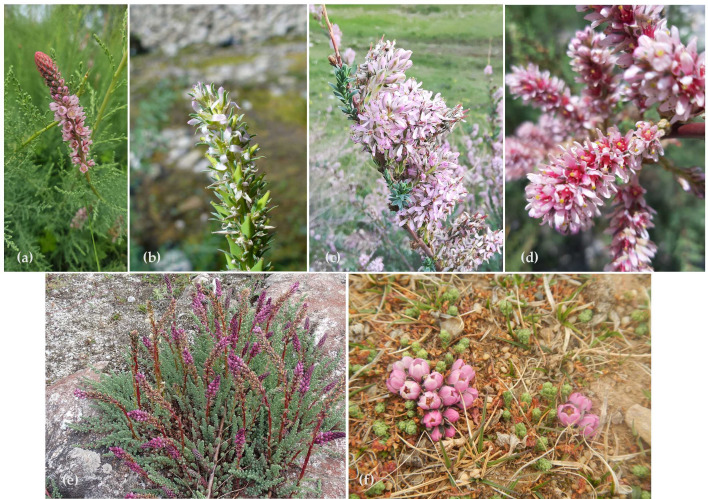
Flowering *Myricaria* species: (**a**) *M. bracteata* (Xthnob the ^CC BY-SA^, https://identify.plantnet.org/k-world-flora/observations/1028722755, accessed on 11 May 2026); (**b**) *M. germanica* (J. Makowska-Wąs); (**c**) *M. longifolia* (kholboevas ^CC BY-NC^, www.inaturalist.org/photos/159369503, accessed on 11 May 2026); (**d**) *M. squamosa* (svetlanasp ^CC BY-NC^, www.inaturalist.org/photos/410170360); (**e**) *M. rosea* (Wim Rubers ^CC BY-NC^, www.inaturalist.org/photos/393031037, accessed on 11 May 2026); (**f**) *M. prostrata* (Chao Shi ^CC 0^, www.inaturalist.org/photos/142668078, accessed on 11 May 2026).

**Figure 4:**
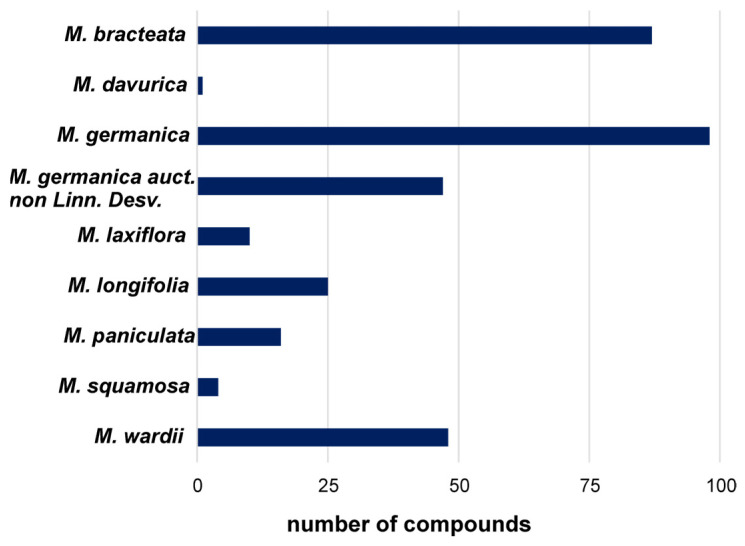
Number of compounds identified in species of the genus *Myricaria*.

**Figure 5:**
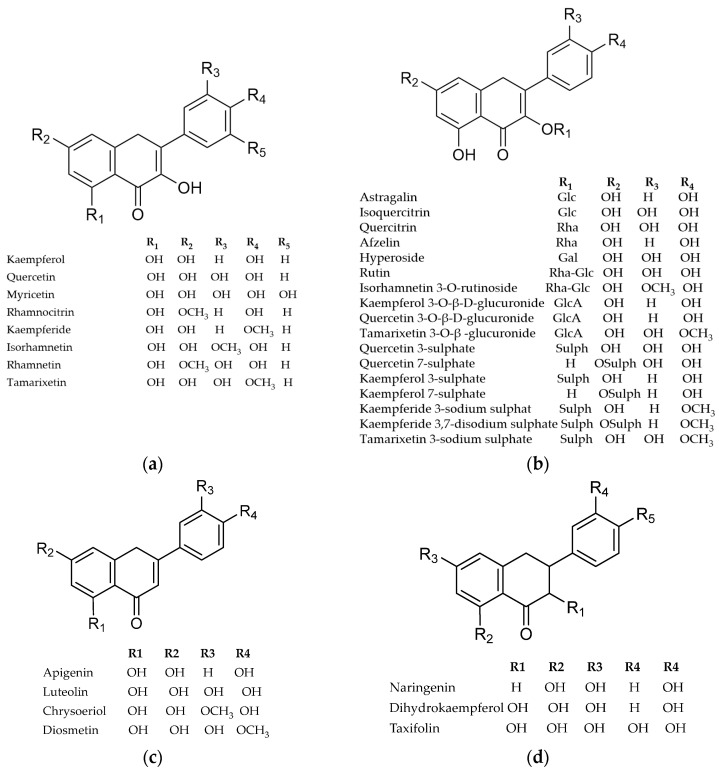
Chemical structures of flavonoids and their derivatives of the genus *Myricaria*: (**a**) Flavonol aglycones; (**b**) Flavonol derivatives (glycosides, glucuronides, and sulfates); (**c**) Flavones; (**d**) Flavanones and flavanonoles.

**Figure 6:**
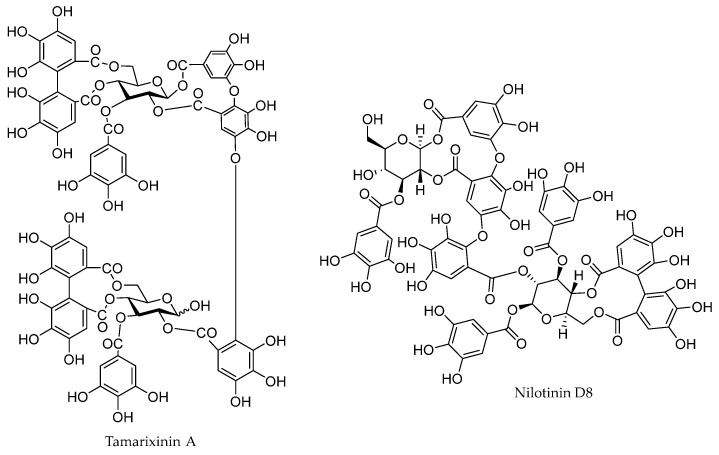
Chemical structures of selected tannins of the genus *Myricaria*.

**Figure 7:**
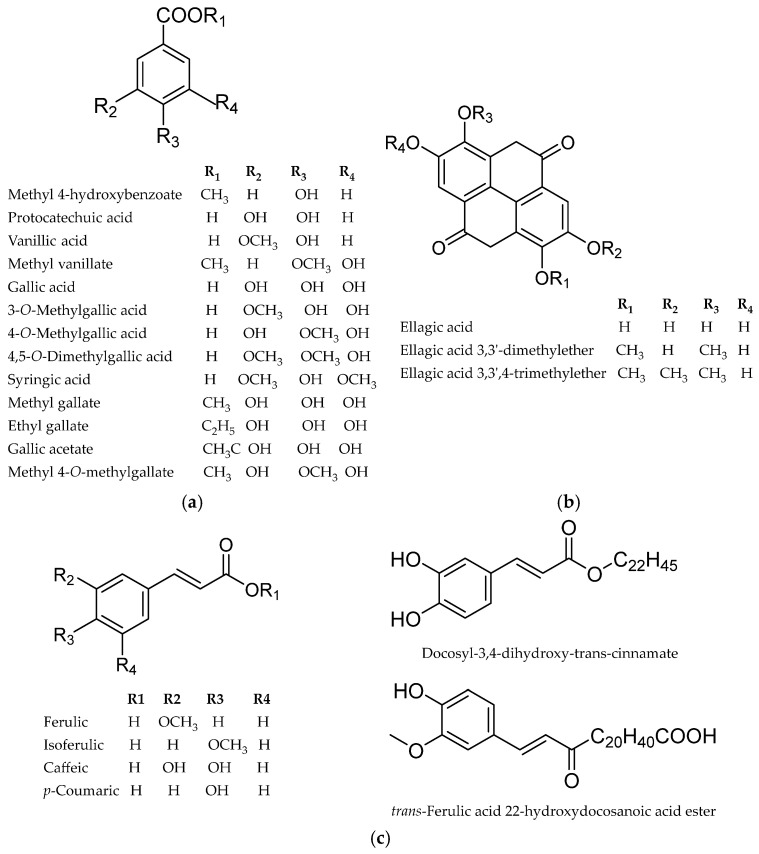
Chemical structures of phenolic acids and their derivatives in *Myricaria* species: (**a**) Gallic acid derivatives; (**b**) Ellagic acid derivatives; (**c**) Cinnamic acid derivatives.

**Figure 8:**
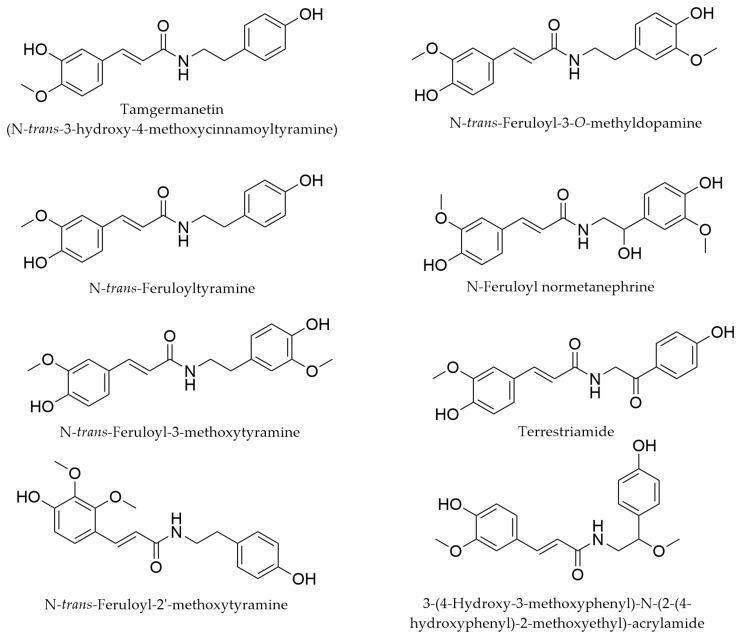
Chemical structures of feruloyl-amides of the genus *Myricaria*.

**Figure 9:**
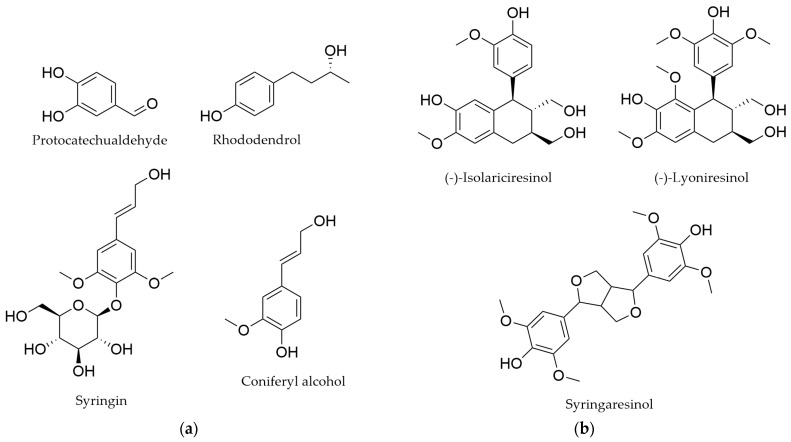
Chemical structures of selected other phenolic compounds of the genus *Myricaria*: (**a**) Other phenols; (**b**) Lignans.

**Figure 10:**
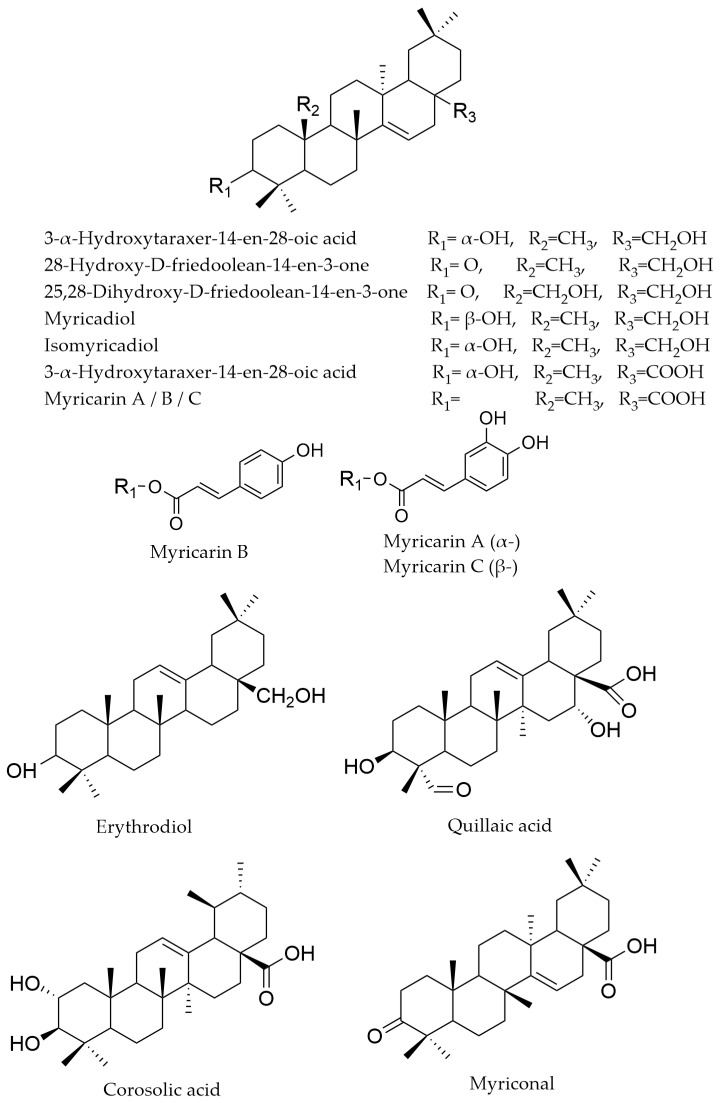

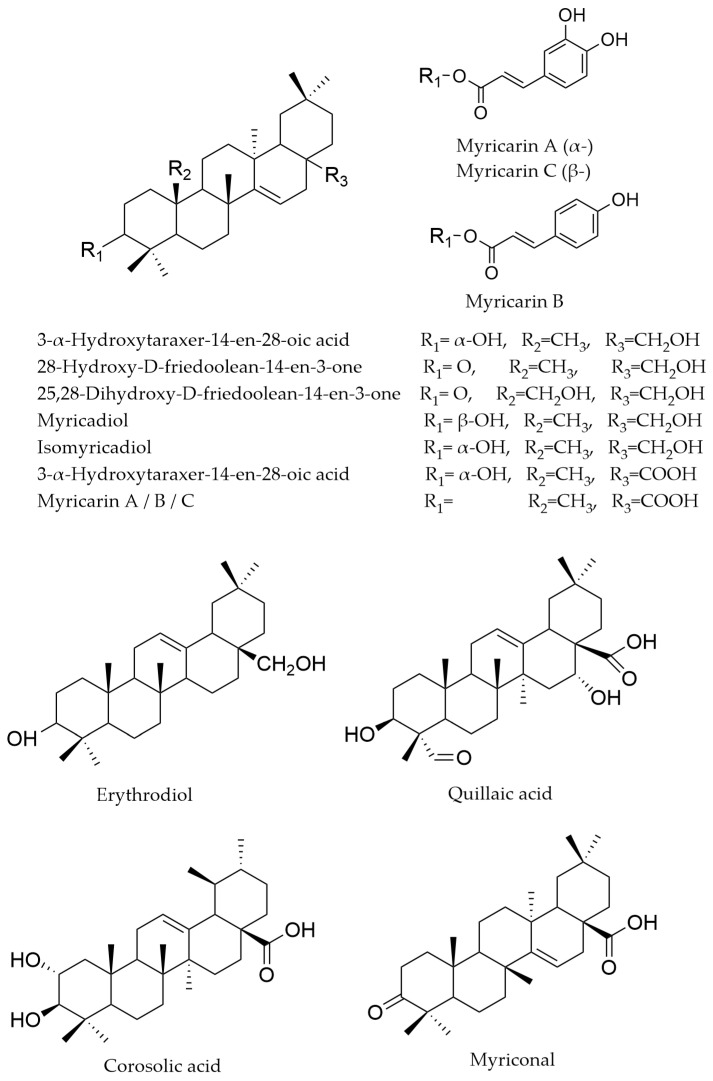
Chemical structures of triterpenoids of the genus *Myricaria*.

**Figure 11:**
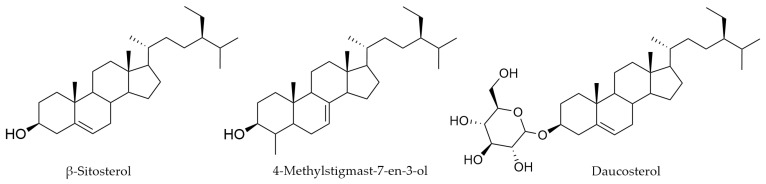
Chemical structures of sterols of the genus *Myricaria*.

**Figure 12:**
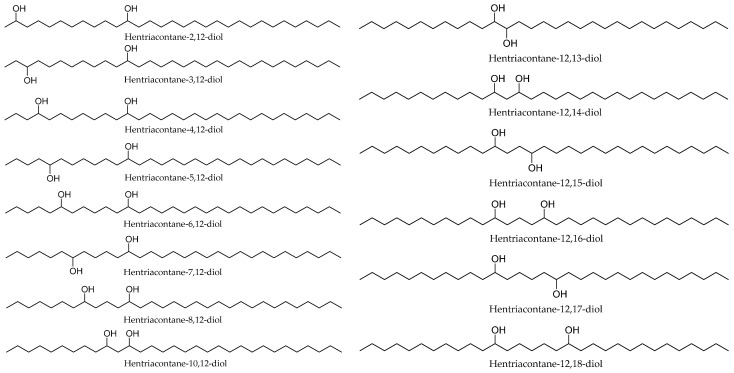
Chemical structures of selected alkanediols of the *Myricaria germanica* leaf wax.

**Figure 13:**
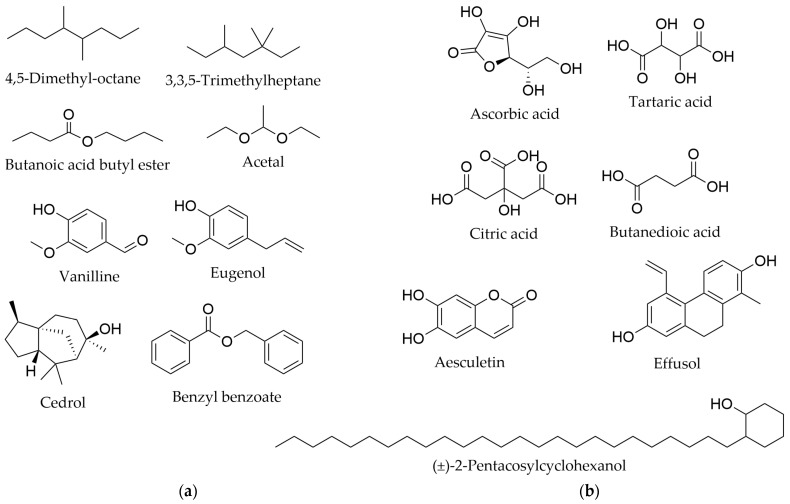
Chemical structures of selected other compounds of the genus *Myricaria*: (**a**) Essential oil; (**b**) Other compounds.

**Figure 14:**
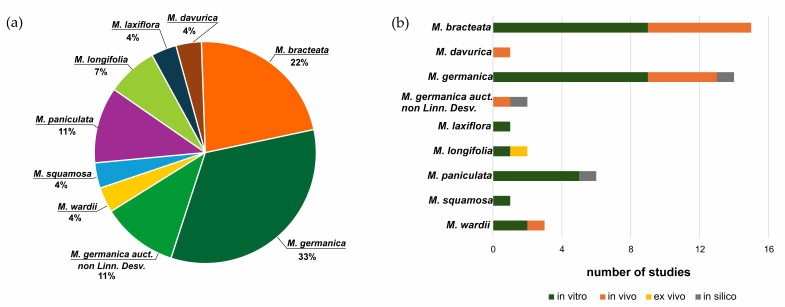
The share of species of the genus *Myricaria* in different types of biological activity studies. (**a**) Share of publications concerning biological studies of species of the genus *Myricaria*; (**b**) Number of studies investigating the biological activity (in vitro, in vivo, ex vivo, in silico) of individual species of the genus *Myricaria*.

**Figure 15:**
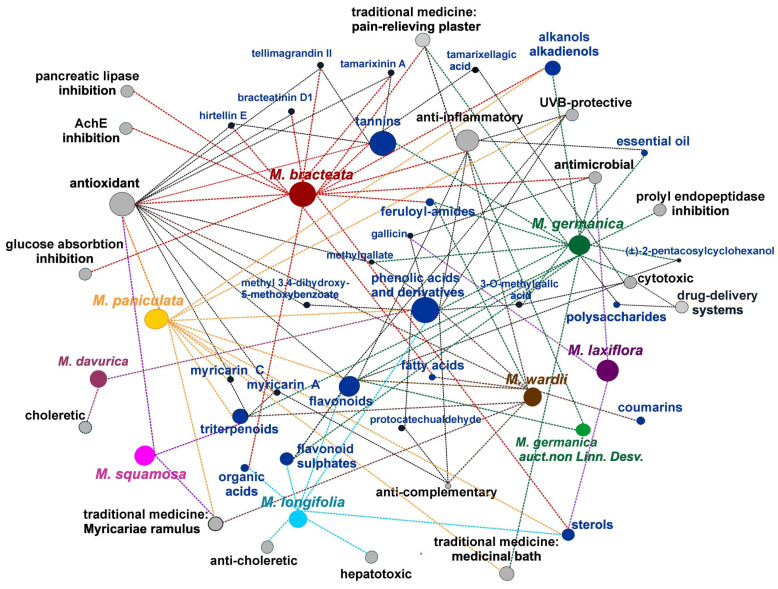
Network of relationships between *Myricaria* species, phytochemicals, and biological activities.

Plant names were verified using the Plants of the World Online database (https://powo.science.kew.org/ accessed on 15 December 2025). Chemical names and their synonym versions were verified in the PubChem database (https://pubchem.ncbi.nlm.nih.gov, accessed on 25 February 2026). SIgnal ChemDraw (Revvity Signals Software, Inc., Waltham, MA, USA; v. 23.1.2.7) software was used to draw the chemical structures of the compounds presented. All figures (Figures 1–14) were prepared using the CorelDraw Graphics Suite program (Corel Corporation, Ottava, ON, Canad; 2025, v. 26.2.0.170). Figure 15 was prepared based on data from collected articles and drawn manually with the use of the same CorelDraw version version (Corel Corporation 2025, v. 26.2.0.170).

All studies were assigned to species according to their currently accepted and taxonomically verified names, as listed in POWO (e.g., *M. alopecuroides* are presented as *M. bracteata*) [1]. The only exception was “*Myricaria germanica* auct. non Linn, Desv.”, for which the original designation of the investigated plant material was retained in accordance with the source publications (see also Section 3.1).

## 3. General Characteristics of *Myricaria* spp.

### 3.1. Taxonomy

According to the Plants of the World Online database, the genus *Myricaria* Desv. comprises 13 accepted species, as listed in Table 1. The Angiosperm Phylogeny Group (APG) classifies Tamaricaceae as part of the order Caryophyllales [5].

The taxonomic status of particular species within the genus *Myricaria* (including subspecies and varieties) has been debated for many years. Individual species are polymorphic and morphologically similar, making them difficult to distinguish. Numerous synonymous names indirectly reflect the long-standing difficulties in the unambiguous identification of individual species [1].

One such example is *M. germanica*. While taxonomic sources other than POWO indicate that *M. germanica* has been treated under synonymous names such as *M. bracteata* or *M. squamosa* [6], in this review, all are treated as separate species, following POWO [1]. Similar discrepancies between botanical sources also apply to *M. davurica* and *M. longifolia* [1,6]. Moreover, the status of the closely related genus *Myrtama* Ovcz. & Kinzik. with its sole representative species *Myrtama elegans* (Royle) Ovcz. & Kinzik. (syn. *Myricaria elegans* Royle) remains controversial [6,7,8,9,10,11].

Thus, when the name *M. germanica* is used in this review, it refers to *M. germanica* (L.) Desv., a species occurring in Europe (see also Table 1). However, in studies based on plant material collected in China (Qinghai) [12,13], a region where *M. germanica* (L.) Desv. does not occur (see also Table 1 and Figure 1) [1,14], we retain the full botanical designation: *Myricaria germanica* auct. non Linn. Desv. to emphasize this distinction.

Interestingly, even the Latin generic name of the genus etymologically refers to their natural habitats. The name derives from the Greek word *myrice*, which comes from *myro*, meaning ‘to run’ or ‘to flee’, describing the growth of the tamarisk bush along the banks of flowing streams [15]. The shrub was mentioned by Theophrastus (370–287 BC) and Dioscorides (40–90 AD). Historically, the names “tamarisk” and “myrice” were attributed to both the genera *Tamarix* and *Myricaria* [2]. The current English name “false tamarisk” is a remnant of this.

**Table 1:** Characterization of *Myricaria* species.

Species ^1^	Synonyms ^1^	Plant Overall Shape	Distribution	Habitat	References
*Myricaria albiflora* Grierson & D.G.Long	not mentioned	erect shrubs; 1–2 m tall	primarily in temperate biome; eastern Himalayas, Tibet; 2130–3050 m amsl	gravel beds by streams and rivers; stony streamsides in the drier inner valleys	[1,16,17]
*Myricaria bracteata* Royle	homotypic: *Myricaria germanica* var. *bracteata* (Royle) Franch. heterotypic: *M. alopecuroides* Schrenk ex Fisch. & C.A.Mey. *M. germanica* var. *alopecuroides* (Schrenk) Maxim. *M. germanica* subsp. *alopecuroides* (Schrenk) Kitam. *M. macrostachya* Kar. & Kir. *M. schartii* Vassilcz.	geoxyl shrubs; 2–2.5 m tall	primarily in the temperate biome; northern-central China, western Himalayas, the Pamirs, the Tien Shan, Sayan Mountains, Altai, North Caucasus, the Crimea; 1500–4200 m amsl; domesticated and used in landscaping	rocky/sandy banks of highland rivers, stream dry canals, pebbles, riparian sand; high winter hardiness and drought resistance	[1,18,19,20,21]
*Myricaria davurica* (Willd.) Ehrenb	homotypic: *Myricaria longifolia* var. *davurica* (Willd.) Maxim (POWO) *Tamarix davurica* Willd. heterotypic: *Myricaria brevifolia* Turcz. *Myricaria dahurica* DC. *Myricaria davurica* var. macrophylla Bunge	shrubs with branches erect to more or less appressed; 2.5–3 m tall	primarily in the temperate biome; South Siberia, Mongolia, western and central Himalayas; 3200–4300 m amsl	gravelly river beds	[1,22,23]
*Myricaria germanica* (L.) Desv. Subspecies: *Myricaria germanica* subsp. *germanica* *Myricaria germanica* subsp. *pakistanica* Qaiser	homotypic: *Tamarix germanica* L., *Tamariscus germanicus* (L.) Scop. heterotypic: *Myrica pannonica* Bubani *Myricaria herbacea* Desv. *Tamariscus decander* Lam. *Tamarix decandra* Salisb. *T. herbacea* Willd. *T. monogyna* Stokes *T. squamosa* Steud.	shrubs with erect and densely leafy twigs, 0.6–3 m tall	subsp. *germanica*: primarily in the temperate biome; North Caucasus, the Carpathian Mountains, the Alps, the Pyrenees, the Scandinavian Mountains introduced to Denmark, Belgium; weed in New Zealand from high-altitude running water habitats (glacier forelands) down to sea level subsp. *pakistanica*: North Pakistan	montane to subalpine riverine floodplains; non-saline limestone and dolomite soils; probably a facultative rheophyte; light-demanding pioneer species	[1,2,3,4]
*Myricaria laxiflora* (Franch.) P.Y.Zhang & Y.J.Zhang	homotypic: *Myricaria germanica* var. *laxiflora* Franch.	erect shrubs; ca. 1.5 m tall	primarily in the temperate biome; north temperate and pantropic vegetation distribution areas between the middle subtropical zone and northern subtropical zone; water-level-fluctuation zone; Three Gorges Reservoir area, along the riverbank of the Yangtze River valley; 70–155 m amsl	low-altitude riversides and shores; roadsides; habitats within the water-level fluctuation zone; clusters in the flooded areas in summer and exposed areas in winter; highly tolerant to river flooding and water submergence	[1,14,24,25]
*Myricaria longifolia* (Willd.) Ehrenb.	homotypic: *Tamarix longifolia* Willd. heterotypic: *Myricaria linearifolia* Desv. *Tamarix decandra* Pall.	shrubs up to 2 m tall	primarily in the temperate biome; Siberia, Mongolia	pebble and stony riverbanks of the mountain rivers; to the tree line	[1,26]
*Myricaria paniculata* P.Y.Zhang et Y.J.Zhang	not mentioned	shrubs, 1–3 m tall	primarily in the temperate biome; northern-central, south-central, and southeast China, Inner Mongolia, Tibet; 1000–2800 m amsl	mountain slopes of river valleys; riparian sand	[1,14,27,28]
*Myricaria platyphylla* Maxim.	not mentioned	erect shrubs; ca. 2 m tall; much branched	primarily in the temperate biome; northern-central China, Inner Mongolia; ca. 1300 m amsl	sandy riverbanks and slopes, lowlands between mobile sand dunes in the desert	[1,14,19,28]
*Myricaria prostrata* Hook.f. & Thomson	homotypic: *Myricaria germanica* var. *prostrata* (Hook.f. & Thomson) Dyer heterotypic: *Myricaria hedinii* Paulsen	dwarf prostrate shrubs or subshrubs; much branched 5–14 cm tall	primarily in the subalpine or subarctic biome low- and high-shrub tundra; northern-central China, Qinghai, Tibet, western Himalayas, Xinjiang; 4000–5200 m amsl	sandy places at lakesides and in river valleys in high mountains, rocky mountain slopes, streamsides, billabongs of hillside; probably a facultative rheophyte	[1,4,14,28,29]
*Myricaria pulcherrima* Batalin	not mentioned	shrubs or subshrubs; rarely much branched; 1–1.5 m tall	primarily in the temperate biome; Xinjiang	sandy riverbanks, lowlands among mountains	[1,14,28]
*Myricaria rosea* W.W.Sm.	not mentioned	prostrate shrubs or subshrubs with densely leafy, numerous ascending branches; ca. 1 m tall	primarily in the subalpine or subarctic biome; native to south-central China, eastern Himalayas, Nepal, Tibet; 2600–4600 m amsl	stream sides in high mountains, rocky mountain slopes; probably a facultative rheophyte	[1,4,14,16,23,28]
*Myricaria squamosa* Desv.	homotypic: *Myricaria dahurica* subsp. *squamosa* (Desv.) P.Fourn. *M. germanica* var. *squamosa* (Desv.) Maxim. heterotypic: *Myricaria armena* Boiss. *M. davurica* var. *microphylla* Bunge *M. hoffmeisteri* Klotzsch	shrubs; ca 2 m high	primarily in the temperate biome; in lower subalpine to upper alpine zones; Central and East Asia from the Altai mountains to the Himalayas; 2400–4600 m amsl	riparian sand, river valleys, sandy and pebbly banks of mountain streams and rivers	[1,19,23,28,29,30]
*Myricaria wardii* C.Marquand	not mentioned	erect shrubs 1–2 m tall	primarily in the subalpine or subarctic biome; Nepal, Tibet; 3000–4000 m amsl	sandy places at riversides	[1,14]

^1^ an accepted name and possibly synonyms for the plant species according to the POWO [1].

### 3.2. Distribution and Habitats

*Myricaria* species primarily grow in temperate biomes [1]. They are distributed in Asia (including the Caucasus, the Central Asian mountain ranges, Siberia, central and northern China, and the Qinghai–Tibetan Plateau) and Europe (the Alps, Scandinavia, and the Balkans) (Table 1) [1,27,31]. Ten species are native to China [14,32]. The distribution ranges of *Myricaria* species are shown in Figure 1.

Most *Myricaria* species occur at high altitudes, with some growing at elevations of 4500–5000 m above sea level. The only exception is *M. laxiflora*, which grows at lower altitudes, 70–160 m a.s.l. (Table 1). They represent pioneer plants that grow almost exclusively on sandy or pebble soils along rivers and streams, and prefer calcareous soils [3,33]. These species are well adapted to short, sudden, and rapid floods [34,35]. Some of them are considered suitable for urban plantings, not only because of their ornamental value, but also due to their winter resistance and high growth rate [33].

The natural habitats of *Myricaria* species are increasingly subject to degradation, primarily due to natural succession processes and anthropogenic pressures such as river regulation and associated hydrological alterations. These factors contribute to the reduction in suitable habitats and may threaten the long-term persistence of local populations. In Europe, a systematic reduction in the number of habitats of *M. germanica* is observed [36,37]. Within the European Union, typical habitat 3230—“Alpine rivers and their ligneous vegetation with *Myricaria germanica*” and others hosting this species (type 3210—“Fennoscandian natural rivers”, 3220—“Alpine rivers and the herbaceous vegetation along their banks”, type 3240—“Alpine rivers and their ligneous vegetation with *Salix elaeagnos*”, and type 3250—“Constantly flowing Mediterranean rivers with *Glaucium flavum*”) can be partially protected [38]. Additionally, the population sizes of Asian species *M. paniculata*, *M. platyphylla*, and *M. pulcherrima* have decreased due to the loss or destruction of natural habitats. Similarly, *M. laxiflora* is considered threatened as a result of river regulation in the Three Gorges Dam project [9,19].

### 3.3. Morphology

Plants of the genus *Myricaria* are deciduous shrubs, rarely subshrubs, with erect or prostrate shoots, and some species can reach up to about 2–3 m in height (Table 1) [1,6,14]. The leaves are small, simple, alternate (helically arranged), sessile, with entire margins, and usually densely arranged along the young branches of the current year. The flowers are bisexual, short-petiolate, and are grouped in terminal or lateral racemes or panicles on spike-like inflorescences. Petals are pink, white, or purplish-red. The fruit is a capsule with three valves (3-septicidal) that contains numerous seeds with white villous awns. Seeds are spread by the wind (Figure 2) [6,14]. *Myricaria* plants produce salt glands, which are characteristic of the family Tamaricaceae, but with calcium/magnesium carbonate/sulfate crystals [3].

#### Features Determining Ornamental Uses of *Myricaria* spp.

*Myricaria* species, which in appearance are very similar to the well-known, popular, and widely cultivated tamarisks (*Tamarix* spp.), are also used as ornamental plants. They include species with upright shoots and decorative flowers, which appear over a long flowering period, combined with attractive foliage coloration (Figure 3). These are shrubs with reddish or yellowish-brown shoots and distinctive, scale-like, glaucous-green leaves that completely cover the shoots. The most popular species used in landscaping are *M. bracteata*, with its terminal inflorescence, and *M. davurica*, with inflorescences located on lateral branches. In nature habitats and cultivation, *M. bracteata* blooms for an extended period (50–60 days) in two phases: sequential flowering of lateral inflorescences (from early June to mid-July) followed by apical inflorescences (from mid-July to early August) [21,39]. The number of simultaneously flowering inflorescences (“brushes”) per shoot ranges from 35 to 40, occasionally reaching up to 60 [21].

Due to their low habitat requirements, *Myricaria* species can be successfully used in gravel gardens, on slopes, and as natural flowering hedges. It can be propagated easily from both cuttings and seeds (however, the germination rate is reduced over time) [40]. They prefer sunny locations and grow well in poor, calcareous, dry, and stony soils. They also tolerate slight soil salinity, periodic flooding, and withstand severe frost without the need for winter protection. However, to achieve a lush and attractive shrub, *Myricaria* is best cultivated in fertile, well-maintained soil.

## 4. Phytochemistry

The available literature indicates that species belonging to the genus *Myricaria* Desv. are a valuable source of phenolic compounds, typical for the Tamaricaceae family [41,42]. Preliminary phytochemical investigations revealed that flavonoids and other phenolic compounds are the major constituents, whereas coumarins, anthraquinones, and alkaloids were not detected [43,44]. More recently, however, a coumarin compound was identified [45].

The first phytochemical studies reporting the isolation of phenolic compounds from *Myricaria* spp. were conducted on *M. bracteata* (described under the synonymic name *M. alopecuroides*) during the late 1960s and early 1970s [46,47,48,49,50,51]. Further studies on various *Myricaria* species led to the isolation of successive flavonoids [52,53,54,55,56,57,58,59,60], phenolic compounds and tannins [53,57,61,62,63], sterols [46,57,58,59,64,65], triterpenoids [64,65,66,67], long-chain fatty alcohols [46,68], and feruloyl-amids [54,62].

Not all species have been studied to the same extent; most reports concern *M. bracteata*, *M. germanica*, and *M. longifolia* (Figure 4). Research has primarily involved aerial plant parts, which are those most commonly used in traditional medicine, although diverse classes of natural products have been confirmed in all vegetative organs. In some studies, the research does not extend to the isolation stage and detailed structural determination based on full spectral analysis. A summary of compounds identified in the genus *Myricaria* Desv. is presented in Table 2. Data concerning the quantitative analysis of phytochemical constituents in plants of the genus *Myricaria* are presented in Table S1 (Supplementary Materials).

### 4.1. Flavonoids

Flavonoids are generally considered one of the most ubiquitous groups of plant secondary metabolites. The main class of flavonoids in Tamaricaceae, as well as in genus *Myricaria*, is flavonols (Figure 5) [82]. Numerous glycosides of quercetin (isoquercetin, quercitrin, hyperoside, rutin) and kaempferol (astragalin, afzelin) have been reported in most species. There are also methyl derivatives of quercetin (like isorhamnetin, rhamnazin, rhamnetin, tamarixetin, dillenetin) and kaempferol (like rhamnocitrin or kaempferide). Myricetin had not previously been detected in Tamaricaceae, and its absence was considered characteristic of the family [72], but it was recently identified in *M. bracteata* [55].

Common flavonols kaempferol and quercetin, and their methylated derivatives, occur as free aglycones and/or glycosides and are present in almost all examined *Myricaria* species (Table 2). Some of them may form glucuronides or sulfates. The latter group is especially interesting because they are uncommon in plants [72,83,84,85]. In fact, they seem to be a characteristic feature of the tamarisk family; in some taxa, 3- or 7-sulfated compounds are most abundant [72,82]. The physiological role of this group is mostly unknown, but the accumulation of sulfate conjugates may be related, e.g., to growing in saline or humid habitats, tolerating alkaline conditions, reactive oxygen stress, and also the regulation of plant growth [54,84].

Sulfated flavonols identified in the genus *Myricaria* include kaempferol 3-sulfate, kaempferol 7-sulfate, quercetin 3-sulfate, quercetin 7-sulfate, kaempferide 3-sodium sulfate, isorhamnetin 3-sulfate, tamarixetin 3-sodium sulfate, and kaempferide 3,7-disodium sulfate (Table 2) [54,72,78,80].

Flavones are less abundant; chrysoeriol and methyl ethers of apigenin and luteolin were found in *M. bracteata* and *M. wardii*, as well as in *M. germanica* auct. non. Linn. Desv. [13,45,55,56,57]. Interestingly, in later species, flavanone C-glycosides (isovitexin and homoorientin) and isoflavone (calycosin-7-*O*-β-D-glucoside) have also been identified for the first time recently [13].

Flavanones, dihydroflavonols, chalcones, or biflavonoids have been rarely isolated and identified in this genus, only in *M. bracteata*, *M. longifolia*, *M. paniculata*, and *M. wardii* [45,55,56,57,58].

The studies focused mostly on the overground plant parts used in traditional medicine. Interestingly, kempferol-3-*O*-rhamnoside and rhamnetin were also identified in the root bark of *M. bracteata* and *M. wardii* [53].

Studies on the quantitative content of phenolic compounds and flavonoids confirm that these are the dominant groups in plants of the genus *Myricaria*. In the flowered green branches of *Myricaria bracteata* collected from the Gobi-Altai aimag (Mongolia), the total phenolic content measured using the Folin–Ciocalteu reagent at pH < 10 was 15.14 ± 1.48% (expressed as gallic acid equivalent from the calibration curve). Total flavonoids determined by spectrophotometry were 2.09 ± 0.03% and 0.61 ± 0.02%, expressed as rutin and quercetin equivalents, respectively. Moreover, isorhamnetin, followed by ethyl gallate and the tannin tellimagrandin II, seems to be characteristic of the branches of this species [69].

In another study, Chernonosov et al. [55] determined the content of phenolic compounds in samples of *M. bracteata* leaves collected from two distant populations (the Altai Republic and the Republic of Tajikistan). In both groups of hydrolyzed aqueous ethanol extracts, the same seventeen constituents were identified, mainly methyl ethers of quercetin and kaempferol. However, significant differences in the levels of quercetin, kaempferol, isorhamnetin, and luteolin were observed. Isorhamnetin dominated in the leaves of plants from Tajikistan, whereas kaempferide and rhamnazin contributed significantly to the total phenolic content in leaves from the Altai [55]. The aerial parts of the same species collected in Kazakhstan (Almaty region) contained 4.12% flavonoids [70].

A comparison of the two Siberian species, *M. bracteata* and *M. longifolia*, in terms of phenolic derivatives with particular emphasis on flavonoids, was conducted by Karpova et al. [20,56,77]. Free compounds and aglycones after hydrolysis were determined in both species, and *M. longifolia* exceeded *M. bracteata* in the concentrations of most identified components, as well as in total flavonoid content (13.44 ± 1.23 and 4.21 ± 0.77 mg/g dw, respectively). Aglycon compositions were rather similar, in the hydrolyzed extracts of the leaves, quercetin, kaempferol, rhamnocitrin, isorhamnetin, rhamnetin, rhamnazin, kaempferide, naringenin, apigenin, luteolin, and chrysoeriol were detected [56]. Furthermore, the authors compared the qualitative and quantitative composition of flavonoids in the salt glands and leaf tissues of both species with their histochemical localization, which demonstrated secretion of these compounds to the leaf surface via the salt glands [20]. The flavonoids exuded by the leaf salt glands were characterized for the first time and showed similarities between the species. The phenolic profiles revealed similarly high levels of total flavonoids and ellagic acid. Astragalin and hyperoside were the predominant flavonoid compounds in the leaves, whereas isorhamnetin was the main aglycone in hydrolyzed samples of both species [20].

### 4.2. Tannins

In addition to flavonoids and their derivatives, the presence of tannins is characteristic of the Tamaricaceae family. Tannins may be responsible for the significant impact on the biological activities of hydrophilic, water-based extracts prepared from *Myricaria* plants. This group of polyphenols exhibits antioxidant, anti-inflammatory, antimicrobial, and antiviral activity [61,86]. Aerial parts of *M. bracteata* from Kazakhstan (Almaty region) contain 7.84% tannins [70].

In the epigeal part of *M. bracteata*, a combined ellagitanin was described which, upon hydrolysis, yielded dehydrodigallic and dehydrotrigallic acids. The same authors later identified simple tannin substances, named myrynin (1,2,3-dehydrotrigaloyl-α-D-glucose), which hydrolyses to free glucose, gallic acid, dehydrodigallic acid, and dehydrotrigallic acid [50,51]. This species also contains a more complex ellagotannin, myrilagin (1,2,3-dehydrotrigalloyl-4,6-hexahydroxydiphenoyl-α-D-glucose), which can decompose into myrynin [87].

Similarly, Liu et al. studied branches of *M*. *bracteata* and identified twelve hydrolyzable tannins. This group included monomers such as nilotin M4 and 1,3-di-*O*-galloyl-4,6-*O*-(*aS*)-hexahydroxydiphenoyl-β-D-glucose. The dimers were represented by bracteatinins D_1_ and D_2_, tamarixinin A, nilotinin D8, hirtellins A, B, and E, and isohirtellin C. The trimers included hirtellin T_3_ and bracteanin T_3_. Three compounds, bracteatinins D_1_, D_2_, and T_1_, were described for the first time and presented a hellinoyl-type structure. The tamarixinin A is a dominating compound in the tannin fractions, at a level 0.14% of dried plant [61].

In the European species *M. germanica*, Nawwar et al. (2013) identified digaloyl-glucose derivatives (2,3-digalloyl-(α/β)-glucose, 1,3-di-*O*-galloyl-β-glucose, 2,4-di-*O*-galloyl-(α/β)-glucopyranose, 2,6-di-*O-* galloyl-(α/β)-glucose) [54].

Tannins identified in false tamarisk species are presented in Figure 6 and Table 2.

### 4.3. Phenolic Acids and Their Derivatives

Phenolic acids and their derivatives have been described in all investigated species of the genus *Myricaria* (Figure 7, Table 2). The main compounds of this group are benzoic acid derivatives such as gallic and ellagic acid, along with their esters. In some species, such as *M. bracteata*, ellagic acid has been identified as the second most abundant phenolic compound in the leaves, reaching up to 5.72 ± 0.22 mg/g dw [55].

Among cinnamic acids and their derivatives, the most widespread are isoferulic acid (*M. bracteata*, *M. longifolia*, *M. paniculata*, *M. wardii*), caffeic acid (*M. bracteata*, *M. germanica*, *M. longifolia*, *M. wardii*), and ferulic acid (*M. bracteata*, *M. germanica*, *M. longifolia*). However, some rare compounds, such as docosyl-3,4-dihydroxy-*trans*-cinnamate and *trans*-ferulic acid 22-hydroxydocosanoic acid ester, were identified in *M. bracteata* [66].

A quantitative study of the phenolic profiles of aqueous ethanol extract of *M. bracteata* and *M. longifolia* revealed differences between populations of both species. Free ferulic acid showed a higher level in *M. bracteata*. Free gallic acid was the main phenolic acid in both species, with concentrations of 7.04 ± 2.33 mg/g dw in *M. bracteata* and 22.70 ± 7.39 mg/g dw in *M. longifolia*. The total phenolic content was also higher in *M. longifolia* than *M. bracteata* (93.34 ± 0.46 and 29.30 ± 0.28 mg/g dw, respectively). The authors used morphological parameters together with phenolic compounds as an indicator of biological diversity of these plants [20,56].

### 4.4. Feruloyl-Amides

A new group of metabolites in *Myricaria germanica* was described by Nawwar et al., including a cytotoxic compound, tamgermanitin, which was identified as an isoferulic acid amide (*N*-*trans*-isoferuloyltyramine) [54]. Additional feruloyltyramine derivatives were identified in *M. bracteata* [62], and quite recently, further feruloyl-amides were described in ethanolic extracts of branches and leafy twigs of *M. wardii* (Figure 8) [45].

### 4.5. Other Phenolics

Various phenolic aldehydes, such as protocatechualdehyde, vanillin, sinapaldehyde, syringaldehyde, rhododendrol, coniferyl aldehyde, and coniferyl alcohol, have been identified in *Myricaria bracteata*, *M. germanica*, and *M. wardii* (Figure 9a) [45,66,74]. In *M. bracteata* and *M. germanica*, lignans have also been identified (Figure 9b) [62,74].

### 4.6. Triterpenoids

Triterpenes have been reported in the aerial parts of several *Myricaria* species, as well as in other representatives of the Tamaricaceae family [88,89,90]. Friedooleanane derivatives were identified in *M. paniculata* and *M. squamosa*. Myricarins A and B are hydroxycinnamate esters substituted at C-3 of the aglycone [64,67]. Other compounds described in these species include myricarin C and 3α-hydroxy-D-friedoolean-14-en-28-oic acid (in *M. squamosa*), as well as myriconal, 28-hydroxy-14-taraxeren-3-one, 28-aldehyde-taraxerenone, and *epi*-friedelanol (in *M. paniculata*), and 25,28-dihydroxy-D-friedoolean-14-en-3-on (in *M. laxiflora*) [58,64,65,67]. In *M. wardii*, ursolic acid has also been identified, together with corosolic acid, whereas quillaic acid has been reported from *M. germanica* (Figure 10) [13,45].

### 4.7. Sterols

β-Sitosterol, the most common plant sterol, has been identified in flowering green branches of *Myricaria bracteata* [46,57,69], in whole plants of *M. laxiflora* [65], and in the aerial parts of *M. longifolia* [79]. In *M. bracteata*, it is accompanied by β-sitosteryl glucopyranose [69]. The stems of *M. paniculata* contain both β-sitosterol and 4-methyl stigmast-7-en-3-ol [58,64]. Furthermore, daucosterol has been identified in *M*. *bracteata* (Figure 11) [57,59].

### 4.8. Long-Chain Fatty Alcohols (Alkanols and Alkanediols)

In the epigeal parts of *Myricaria bracteata* extracted with petroleum ether, Troschenko and Povolotskaya isolated the secondary aliphatic alcohol 12-hentriacontanol [46]. The same compound, together with 1-triacontanol, was identified later in the stem of *M. paniculata* [64].

A comprehensive analysis of lipophilic long-chain alkanediols in the leaf surface waxes of *M. germanica* was performed by Jetter (2000). All groups were characterized by both their proportional contribution to the total wax fraction and their absolute content per unit leaf area. The study revealed four major series: (a) hentriacontanediol isomers (C_31_) with a hydroxyl group in the 12-position, with the second hydroxyl group occurring at positions ranging from 2 to 18; (b) alkanediols (C_30_–C_34_), containing one hydroxyl group on a primary and the other on a secondary carbon atom; (c) homologous series of β-diols (C_25_–C_43_) with predominant 8,10- and 10,12-functionalities; (d) homologous series of γ-diols (C_39_–C_43_) mainly with 8,11- and 10,13-isomers. Selected representative compounds are presented in Figure 12. Among these groups, the series of hentriacontanediol isomers and the homologous β-alkanediols were dominant, reaching 3.5 and 0.6 mg per cm^2^ of leaf surface area, respectively. These values corresponded to 9% and 2% of the total wax mixture [68].

### 4.9. Other Compounds

Other phytochemicals (Figure 13) reported from various *Myricaria* species include coumarins. Among them, only aesculetin has been tentatively identified in the branches and leafy twigs of *M. wardii* [45]. In the aerial parts of *M. germanica* growing in northern Pakistan, the compound (±)-2-pentacosylcyclohexanol, an agent with interesting biological activity, was described (see Section 5.2.9 ) [73].

Essential oil obtained by 8-h steam distillation from the leaves of *M. germanica* was characterized with an extraction yield of 0.183%. The authors identified a total of 90 constituents. The major groups of compounds were fat and aromatic hydrocarbons and their esters. The highest relative contents were noted for octadecane (7.69%), 1,6,7-trimethylnaphthalene (5.43%), 1-tetradecene (4.61%), *m*-xylene (4.17%), and benzyl benzoate (4.1%). However, the authors emphasized the relevance of bioactive constituents present in smaller amounts, including linalool (0.17%), eugenol (0.35%), cedrol (0.36%), and 3,5-di-*tert*-butyl-4-hydroxybenzaldehyde (0.21%) [91,92]. The list of identified compounds and their relative content in the essential oil fraction is presented in the Supplementary File (Table S2).

Organic acids were among the major groups of metabolites identified in the leaves of *M. bracteata* and *M. longifolia*, with total organic acid content (calculated as ascorbic acid equivalent) ranging from 7.25 ± 1.71 to 7.30 ± 2.44, respectively [56]. In *M. bracteata* leaves, citric and tartaric acids were reported [55,56]. The presence of these acids, along with the synthesis and accumulation of sulfate conjugates of phenylpropanoids, flavonols, and other phenolics, suggests that these features are characteristic of alkali-tolerant plants [54,93].

The aerial parts of *M. bracteata* contain 6.77% carbohydrates [70]. Some research groups have investigated the activity of a modified polysaccharide, previously isolated from *M. germanica*, but did not report any structure details [75,94]. This compound may be of particular interest, since flavonoid-substituted polysaccharides with anticomplementary activity have recently been isolated from another species of the Tamaricaceae family, *Tamarix chinensis* [95,96,97].

Both saturated and unsaturated fatty acids have been reported from the aerial parts of *Myricaria* species. Palmitic and stearic acids, along with stearic acid ethyl ester and 1-monopalmitin, were detected in *M. bracteata* [59,69]. The compound 6,7,10-trihydroxy-8-octadecenoic acid represents an unsaturated hydroxy fatty acid derivative in this species [59]. In a study conducted on plants collected in Kazakhstan, unsaturated fatty acids predominated, including linoleic acid (47.8%) and oleic acid (33.4%), whereas palmitic acid (10.6%) represented the predominant saturated fatty acid [70]. In the branches and leafy twigs of *M. wardii*, Zhang et al. identified 9,12,13-trihydroxy-10,15-octadecadienoic acid, 8,11,12-trihydroxy-9-octadecenoic acid, and linoleic acid. Myristic acid, however, was detected only in branches, whereas hexadecenoic acid was found only in leafy twigs [45].

The historical literature reports that the leaves of *M. bracteata* contain vitamin C (83.3 mg%) [98], and a later study confirmed its presence at 10.1 mg/100 g [69]. In the aerial parts of this species, the content of vitamin E was also determined (3.5 mg/100 g) [70].

For the aerial parts of *M. bracteata*, the quality of raw plant material originating from Kazakhstan was assessed. Total ash was 5.78%, and sulfate ash reached 10.85%. Among macro and microelements, the highest levels were observed for iron (3.38%) and potassium (0.35%) [70].

## 5. Traditional Uses and Current Pharmacological Investigations

### 5.1. Traditional Uses

Before their potential as ornamental plants was recognized, species of the genus *Myricaria* were primarily used and valued in traditional medicine, particularly in the regions where they naturally occur. The medicinal properties of the bark of European false tamarisk have been known since at least the 16th century [2,15]. Asian species growing in specific mountain habitats have been a part of traditional Tibetan and Mongolian medicine. The remaining data, including ethnopharmacological studies focusing on the folk medicinal use of these plants, refer primarily to Siberian regions and, to a lesser extent, to ethnic minorities in Himalayan areas (Table 3).

Most of the data presented in this section are derived from secondary sources, primarily review articles, including regional monographs on medicinal plants. Only limited information originates from primary studies, particularly ethnobotanical and ethnopharmacological research, documenting the traditional medicinal use of these plants among selected tribal communities or ethnic minorities. Such information concerns four species (*M. bracteata*, *M. squamosa*, *M. rosea*, and *M. germanica*), mainly from the Himalayan region. These data are indicated in Table 3 wherever a reference to a specific region is provided.

#### 5.1.1. Siberia, Central Asia, and Mongolia

In southern Siberia, there are strong influences of Tibetan medicine, including Traditional Mongolian Medicine [104], which is practiced also in regions neighboring Mongolia, such as Buryatia and Inner Mongolia.

*Myricaria longifolia*, which is the most typical Siberian *Myricaria* species, is well known in folk medicine. Traditionally, plant shoots are used in rheumatism, in diseases of the urinary and reproductive system in women, and as an anti-inflammatory, diuretic, and anticonvulsant agent [52]. *M*. *longifolia* is used as part of multicomponent traditional Mongolian compositions to treat fever, liver and biliary tract disease, to improve skin tone, wound healing, dietary errors, and poisoning [20,52,103]. The species is described as “sour and sweet”, and the potency is “blunt and cool” [103]. Their shoots were also an ingredient of the complex drug “taban-arshan”, prepared by lamas or amchis (traditional doctors or healers) for diseases of the skin, kidney, blood, joints, bones, and women’s ailments [52].

Other species occurring in this area are used in a similar manner in traditional practices, including Mongolian medicine (Table 3). The shoots of *M. bracteata* are used in the treatment of fever and for counteracting toxicity, as part of multicomponent formulations similar to those described for the previous species [99]. Another traditional formulation for spleen disorders from the Inner Mongolia region includes this species under the name “Myricariae Ramulus” (see also Section 5.1.2) [134]. Other traditional uses are presented in Table 3.

A third species occurring in this area, *M. davurica*, is used mainly for respiratory and biliary diseases [101,102].

#### 5.1.2. Himalayas and Tibetan Plateau

*Myricaria bracteata*, a widespread species, is used in the treatment of rheumatism and arthritis, as well as a blood purifier [61,107]. Ethnopharmacological studies conducted in the state of Uttaranchal, India, confirm the use of this plant in rheumatic diseases [106].

On the Tibetan Plateau, the branches and leafy twigs of local *Myricaria* species under the name “Myricariae Ramulus” have been used as a heat-clearing and detoxifying agent in infections, sore throat, scalds, joint pain, arthritis, and other inflammatory conditions. Other traditionally used *Myricaria* species include *M. paniculata*, *M. squamosa*, and *M. wardii*, as well as *M. bracteata* and a closely related species, *Myrtama elegans* [45,134]. *Myricaria squamosa* is used to treat blood fever, exterior syndrome, poisoning, and as an antitussive and febrifuge agent [67,122]. In traditional Tibetan medicine, *M. prostrata* is used to neutralize meat poison, compounded poison, and bile fevers [110].

Another important *Myricaria* species used in Tibetan medicine is *M. rosea*. Its classification is like that of other species: the taste is described as “sweet and astringent”, and its potency as “cool”. Data from primary sources on different areas of Nepal show that leaves and flowers collected by *amchis* primarily at the time of flowering are used for fever, headache, stomachache, and uterine bleeding. The plant is also used in herbal baths and as an antidote to food and meat poisoning. It is considered non-toxic and is commonly used in combination with other herbs [112]. Furthermore, a decoction prepared from the leaves, stems, and flowers is taken orally for lung diseases and asthma [114,117]. Also in Bhutan, aerial parts of *M. rosea* are used as a febrifuge and resolvent, to eliminate dropsy and cough, and are included in complex formulations [113].

Chinese authors have investigated the overview of ethnic medicine therapies of rheumatoid arthritis and summarized the uses of *Myricaria* species in dried overground parts of Tibetan and Mongolian medicine in medicinal bath therapy (*M. germanica*, *M. paniculata*, and* Myricaria* sp.) [135,136]. These species are used in medicinal bath therapies in complex preparations (Wuwei Ganlu), especially with *Ephedra* sp., *Juniperus* sp., *Artemisia sieversiana* Ehrh., and *Rhododendron anthopogonoides* Maxim, or as a part of the complex extract in medicinal plasters (such as CheeZheng Pain Relieving Plaster) [136].

Several studies have also aimed to clarify the mechanisms of anti-inflammatory action of traditional complex remedies or formulas that include *Myricaria* species. Numerous investigations, conducted primarily in China, examined the traditional pain-relieving plaster (PRP), a topical Tibetan medicinal preparation composed mainly of *Phlomoides rotata* (syn. *Lamiophlomis rotata*) and *Curcuma longa*, along with other herbal components such as *Oxytropis falcata* and *Myricaria bracteata*. PRP exerts anti-inflammatory and circulation-improving effects, relieves pain, and reduces stasis [137,138]. Its anti-inflammatory activity in macrophage models appears to derive from the suppression of key inflammatory mediators, including TNF-α, IL-1β, and the inducible cyclooxygenase enzyme (COX-2), as well as its downstream metabolite PGE_2_, primarily through modulation of the NF-κB signaling pathway. Furthermore, PRP markedly reduced LTB_4_ production, indicating inhibition of the lipoxygenase (5-LOX) pathway, which likely represents an additional mechanism contributing to its anti-inflammatory effects [137]. Collectively, these findings indicate that *M. bracteata* in PRP may contribute to the dual inhibition of the COX-2 and 5-LOX pathways and suppression of NF-κB–mediated signaling, supporting its traditional use in inflammatory conditions.

A systematic analysis of clinical studies of the CheeZheng PRP—which contains *Phlomoides rotata* (Du Yi Wei), *Curcuma longa* (Jiang Huang), *Oxytropis falcata* (Ji Dou), *Myricaria germanica* (Shui Bai Zhi), as well as *Zanthoxylum bungeanum* (Chuan Jiao), *Carthamus tinctorius* (Hong Hua), and camphor (3%)—indicates promising clinical effects in the treatment of musculoskeletal pain with minimal adverse reactions. However, further rigorously designed, placebo-controlled studies are needed to determine the efficacy and safety of this product for pain relief in different patient groups, as well as an unambiguous identification of the active ingredients of this complex product [139].

It is important to emphasize, however, that the presented studies concern complex products, often with insufficiently characterized phytochemical composition of plant raw materials, so it is difficult to unambiguously assign individual activities or mechanisms to individual plants, extracts, or compounds. There are some reports and patents on the uses of *M. germanica* extracts in other complex medical or cosmetic formulas, such as preparations for medicated baths, moisturizing soap, or medical toothpaste [43,115].

#### 5.1.3. Western Himalayas

The western Himalayas are the regions in which *Myricaria germanica* subsp. *pakistanica* is found. In this area, the traditional use of *M. germanica* under the names “Umbo/Umboo” or “Ombo” remains common. Ethnopharmacological studies have focused on northern India (Ladakh, Kashmir, Himachal Pradesh, Uttaranchal) and Pakistan (Gilgit-Baltistan) [125,127,128,129,130]. In Ladakh, an infusion of dried leaves of this species is still used as an analgesic, for the management of chronic bronchitis, and as a blood purifier [127,128,132]. In the Lahaul-Spiti region, juice extracted from fresh young shoots and leaves is administered orally for rheumatism to relieve joint pain and swelling [129,130]. Further ethnopharmacological reports from this region are presented in Table 3.

#### 5.1.4. Europe

In the early centuries, members of the Tamaricaceae family were used in the treatment of many ailments, and it is difficult to identify whether these data refer to *Tamarix* or *Myricaria* species. The astringent properties of the bark of these plants were described by Matthioli (1501–1578), who noted its astringent nature [2,15]. German tamarisk (*Myricaria germanica*) was used especially for spleen disease. In *Pharmacopoea universalis* (1835), the actual use of the bark of the two *Tamarix*/*Myricaria* species (*Cortex Tamarisci gallici* and *Cortex Tamarisci germanici*) was described as “obsolete” [140]. Over time, its use in Europe was systematically discontinued.

Despite this, there is some information about occasional use of *M. germanica* in Europe, e.g., in the treatment of jaundice, as an analgesic, or as a substitute for hops (as a bitter ingredient) [2,40,132,141]. In areas of South Tyrol (Italy), residents formerly collected branches of *M. germanica* to obtain tamarisk oil, used for inhalation and rubbing, similarly to mountain pine oil [131]. An infusion of dried leaves of this species is still used as an analgesic or to control chronic bronchitis [128,132]. The juice extracted from fresh young shoots and leaves is used orally in rheumatism to relieve joint pain and swelling (Table 3) [129,130].

### 5.2. Pharmacological Studies

The history of research on the pharmacological activity of species belonging to the genus *Myricaria* is relatively short, essentially covering the last two decades. The limited scientific interest in this genus is likely related to its restricted geographical distribution—endemic in some cases or confined to remote high mountain regions—as well as its modest use in local traditional medicine (primarily Mongolian and Tibetan). In recent years, however, an increasing number of publications have reported various directions of biological activity of extracts and isolated compounds, as well as their potential applications. Nevertheless, to date, more comprehensive investigations have been conducted only for *Myricaria bracteata* and *M. germanica* (Figure 14).

As phytochemical analyses of the genus *Myricaria* have demonstrated a high content of polyphenols, a substantial proportion of pharmacological studies focus on compounds from the groups of flavonoids, tannins, and phenolic acid derivatives, including assessments of antioxidant and anti-inflammatory properties, as well as the inhibition of various enzymes. The results of in vitro and in vivo studies on extracts and individual compounds isolated from different *Myricaria* species are summarized in Table 4. It should be noted that the majority of the experimental evidence is derived from in vitro experiments (approximately twice as many as in vivo studies). In recent years (2024–2025), a limited number of in silico studies based on molecular docking have also been published (Figure 14). It is noteworthy that no human studies have been conducted to date.

#### 5.2.1. Antioxidant Activity

Due to the high content of phenolic compounds in plants of the genus *Myricaria*, among which flavonoid derivatives, tannins, and phenylcarboxylic acids predominate, several species have demonstrated in vitro antioxidant activity. The studies were conducted both on extracts and on isolated compounds.

##### Extract-Level Evidence

Studies on the antioxidant activity of *M. bracteata* have been conducted primarily on polyphenol-rich ethyl acetate (EA) and *n*-butanol (BuOH) fractions of alcoholic extracts. The antioxidant activity of the flowering green branches of *M. bracteata* collected from the Gobi-Altai aimag in Mongolia was determined by Gendaram [69]. The DPPH scavenging activity of the EA and BuOH fractions was comparable but lower than the positive control, rutin (IC_50_ 22.66 ± 0.29 µg/mL), and was determined as 27.11 ± 0.58 µg/mL and 26.14 ± 0.31 µg/mL, respectively. The ethanol extract of *M. bracteata* (31.93 ± 0.48 µg/mL) was better than the activity of the related water extract (52.35 ± 0.85 µg/mL). Among the fractions differing in polarity, the polar EA fraction exhibited the highest antioxidant activity, whereas the nonpolar DCM fraction was not active [69].

The branches and leafy twigs of *M. wardii* collected from different regions of Tibet were evaluated for antioxidant activity by Zhang et al. [45]. In the ABTS assay, the radical scavenging activity of the ethyl acetate fractions IC_50_ value ranged from 117.00 to 249.70 µg/mL for branches and from 35.79 to 78.02 µg/mL for leafy twigs, indicating weaker activity compared to Trolox, used as a positive control (IC_50_ 47.48 µg/mL). Only one sample—leafy twigs—showed better activity than control (IC_50_ 35.79 µg/mL).

In the study by Bao et al., the antioxidant activities of Tibetan herbs classified as “cold-nature” and “hot-nature” were investigated. The activities of cold-nature herb *M. paniculata* in ABTS^•+^ assay, superoxide anion (O_2_^•−^) scavenging assay, and FRAP test were 145.93 ± 6.08, 316.18 ± 12.16, and 112.3 ± 7.25, respectively. The water extract also inhibited lipid peroxidation in rat liver mitochondria exposed to ferrous chloride, hydrogen peroxide, and ascorbate, as evidenced by inhibition of TBARS formation at a concentration of 1 mg/mL. *M. paniculata*, as well as other cold-nature tested herbs, exhibited higher activities than hot-nature ones [108].

All these findings were corroborated with detailed phytochemical analysis showing that *Myricaria* species contain considerable amounts of different classes of polyphenols, which are known for their capacity to neutralize free radicals and protect against oxidative damage.

##### Compound-Level Evidence

In a study by Liu et al. [61], tannins isolated from the twigs of *Myricaria bracteata* originating from China showed moderate to high antioxidant activity. The highest hydroxyl radical scavenging rates, compared to gallic acid (15.66 μM), were exhibited by the dimeric ellagitannins bracteatinin 1 and hirtellin E (IC_50_ values 15.8 and 16.3 μM, respectively). The twelve examined compounds displayed DPPH radical scavenging activity higher than that of BHT, Trolox, or gallic acid. The tannins also suppressed the content of malondialdehyde (MDA), a mitochondrial lipoperoxidation product induced by Fe^2+^-cysteine [61]. In another study, among the compounds isolated from *M. bracteata* (Table 4), the ellagitannin tellimagrandin II displayed particularly potent antioxidant properties in the DPPH assay (IC_50_ 5.97 µM), better than rutin (IC_50_ 38.7 µM) [69].

Four isolated potential quality markers of *M. wardii* branches and leafy twigs—phenolic compounds (methyl 3,4-dihydroxy-5-methoxybenzoate, protocatechualdehyde, and protocatechuic acid) and a triterpene myricarin A—exhibited antioxidant activities, as shown in Table 4 [45].

Several compounds isolated from *M. germanica* leaves collected in Pakistan exhibited significant β-cell protection, reducing H_2_O_2_-induced oxidative stress. Between three phenolic acid derivatives (methyl gallate and syringic acid) and aliphatic acid butanedioic acid, only methyl gallate shows significant activity by inhibition of oxidative stress-mediated apoptosis in MIN6 cells in different concentrations (50–400 μM), with the highest activity at 50 and 100 μM [145].

In addition to polyphenolic compounds, Myricaria triterpenes were also examined for their antioxidant activity. Four triterpenoids isolated from the aerial parts of *M. squamosa* were evaluated for their free radical-scavenging properties using rutin as a positive control. In the DPPH assay, the EC_50_ values of myricarin A and myricarin C were 40.9 and 42.22 μg/mL, respectively, whereas myricarin B exhibited weaker activity, and 3-α-hydroxy-D-friedoolean-14-en-28-oic acid was inactive. The activity levels were significantly lower than those of rutin (2.7 μg/mL). The antioxidant capacity of myricarin A and myricarin C is attributed to the presence of contiguous phenolic hydroxyl groups in the caffeic acid moiety substituted at C-3 of the pentacyclic triterpenoid skeleton [67]. Myricarin A isolated from the aerial twigs and branches of *M. wardii* shows antioxidant activity comparable to Trolox in the ABTS assay and also displays anti-complementary activity (see Section 5.2.12). Interestingly, both the activity assays and the chemical profiles revealed marked differences between the plant parts (branches and leafy twigs), with the branches containing substantially higher levels of myricarin A, which can be important for plant use practices [45].

#### 5.2.2. Anti-Inflammatory Activity

Anti-inflammatory activity is among the best-documented properties of the *Myricaria* genus. The conducted experiments demonstrate the preclinical efficacy of both the extracts and the isolated compounds. This evidence is mostly derived from in vivo studies.

##### Extract-Level Evidence

Zhang et al. verified the anti-inflammatory potential of leafy twigs and branches of a herbal drug, known under the common name “Myricariae Ramulus”, which is traditionally used to treat inflammatory diseases [45]. In this study, the 95% ethanol extract and its PE, EA, BuOH, and water fractions were administered to male mice at a dose of 100 mg/kg three times (once per day), followed by injection of lipopolysaccharide (LPS, 10 mg/kg). The anti-inflammatory activity was assessed by measuring reductions in IL-6 levels in serum and lung tissue, as well as inhibition of myeloperoxidase (MPO) activity in lung tissue. The crude 95% ethanol extract, EA fraction, and water fraction demonstrated better effects than the lipophilic fractions. Histopathological examination confirmed that the extracts alleviated LPS-induced lung tissue damage [45]. The conclusion from the study was that the most popular and safe solvents, ethanol and water, as well as ethyl acetate, were considered the most effective. Further analysis of other samples of “Myricariae Ramulus” of different plant origins and medicinal parts (identified as branches of *Myricaria squamosa* and *Myrtama elegans* or leafy twigs of *Myricaria wardii* and *Myrtama elegans*) shows the differences in their anti-inflammatory activity and chemical composition (see Table 2). Spectrum-effect relationship analysis identified several constituents as potential quality markers, including methyl 3,4-dihydroxy-5-methoxybenzoate, myricarin A, protocatechualdehyde, N-feruloyl normetanephrine (for branches), and protocatechuic acid (for leafy twigs), with a notably high abundance of myricarin A in branches. These findings highlight the need for clear differentiation of plant parts in clinical use and quality control. Importantly, the identified marker compounds were shown to possess both antioxidant and anti-complementary activities (see Section 5.2.1 and Section 5.2.12), supporting their relevance to the bioactivity of the extracts.

In a recent study, the authors combined network pharmacology, molecular docking, and experimental validation to assess the inhibitory effects of *M. germanica* essential oil on UVB-induced inflammation in HaCaT keratinocytes. In vitro experiments demonstrated that essential oil obtained from plants growing in the Qinghai–Tibet Plateau effectively reduced TNF-α, IL-6, and caspase-3 levels, decreased intracellular reactive oxygen species (ROS) and malondialdehyde content, and enhanced superoxide dismutase (SOD) activity, thereby inhibiting UVB-induced skin inflammation. Network pharmacology and docking approaches identified potential molecular targets and predicted mechanisms of action, indicating benzyl benzoate, di-*tert*-butyl-*p*-cresol, and vanillin as the principal active constituents [146].

##### Compound-Level Evidence

Liu et al. evaluated the in vivo anti-inflammatory activity of *Myricaria bracteata* and identified the dimeric hydrolyzable tannin tamarixinin A as the major active and quantitative dominating in dried plant constituents among twelve compounds isolated from twigs [61]. Tamarixinin A exhibited dose-dependent reduction of croton oil-induced ear edema in mice by 69.8% at 200 mg/kg and moderate suppression of collagen-induced arthritis by 46.0% at 20 mg/kg on day 57. However, cell-based assays showed only marginal inhibition of nitric oxide (NO), TNF-α, and IL-6 production in LPS-stimulated murine macrophages (vs. dexamethasone). However, the viability rates of macrophages increased significantly, so the mechanism of anti-inflammatory activity is still not clear. Nevertheless, the compound demonstrated strong free radical-scavenging properties, suggesting that its anti-inflammatory effect may, at least partly, be linked to antioxidant mechanisms. Notably, O-methylation of tamarixinin A, which eliminated free phenolic hydroxyl groups, abolished both antioxidant and anti-inflammatory activities, underscoring the structural importance of these groups [61].

In another study, the antiarthritic potential of tamarixinin A was further demonstrated in a comprehensive study by Zhuang et al. using two complementary animal models—collagen-induced arthritis (CIA) in DBA/1 mice and adjuvant-induced arthritis (AIA) in Wistar rats [142]. Tamarixinin A markedly alleviates arthritic symptoms, including paw swelling, joint inflammation, and cartilage/bone destruction, significantly lowering clinical arthritis scores, suppressing inflammatory cell infiltration, and improving histopathological indicators of synovial hyperplasia, pannus formation, and joint erosion. Interestingly, in this study, tamarixinin A reduced serum concentrations of pro-inflammatory cytokines TNF-α and IL-1β in CIA mice, while in AIA rats, it lowered IL-6 and IL-1β levels in joint exudates, indicating a consistent anti-inflammatory effect across models. In vitro, in LPS-stimulated murine macrophages, tamarixinin A inhibited NO, TNF-α, and IL-6 production, suppressed inducible nitric oxide synthase (iNOS), and downregulated phosphorylation of key MAPK pathway components (ERK, JNK, p38) as well as NF-κB, p65, additionally blocking p38 nuclear translocation. Collectively, these data show that tamarixinin A mitigates joint destruction and inflammation through coordinated inhibition of MAPK and NF-κB pathways, positioning it as a promising natural therapeutic candidate for rheumatoid arthritis [142].

##### In Silico Evidence

Inflammation accompanied by the production of reactive oxygen species contributes to oxidative stress, which in turn further intensifies the inflammatory response. In addition, excessive complement system activation can initiate acute inflammatory reactions, speed up disease progression, and lead to systemic inflammatory disorders. Consequently, anti-complementary and antioxidant properties are considered two key mechanisms underlying the anti-inflammatory effects of traditional herbal medicines [45].

The molecular mechanism underlying the antirheumatoid arthritis activity of *Myricaria* plants was elucidated using network pharmacology and molecular docking analyses. Based on the identification of active compounds present in the branches and leaves of *Myricaria germanica* auct. non Linn. Desv. collected in Qinghai (China), their potential molecular targets and mechanism of action were determined (the study predicted their potential molecular targets and pathways). The key active substances contributing to anti-RA activity were the flavonoids apigenin and isorhamnetin, and the triterpenoid quillaic acid. The main predicted molecular targets included MMP-9, PTGS2, and TNF, while the most significantly enriched signaling pathways were IL-17, relaxin, and TNF [13].

#### 5.2.3. Antimicrobial and Antifungal Activity

As shown in Table 4, the vast majority of data on antimicrobial and antifungal activity of *Myricaria* species is preliminary in nature and comes from in vitro studies on extracts.

##### Extract-Level Evidence

The whole herb of *M. bracteata* extract and fractions demonstrated particularly potent antibacterial activity. The inhibition zones produced by the crude 80% ethanol extract against *Staphylococcus aureus*, *Micrococcus luteus*, and *Enterococcus faecalis* were 11.7, 13.7, and 12.2 mm, respectively. Among the examined fractions, the EA fraction showed the highest activity, yielding inhibition zones of 14.6 mm against both *S. aureus* and *M. luteus*. The same fraction was active against *Pseudomonas aeruginosa*, while the crude extract was inactive. The minimal inhibition concentration (MIC) for the crude extract was 2 mg/disc against *S. aureus* and *M. luteus* [143].

In another study, antibacterial and antifungal activity was evaluated using in situ exposure of microorganisms to the volatile fraction released by living *M. bracteata* plants. The highest relative reduction in colony number compared with the control was observed for *Staphylococcus epidermidis* (66 ± 2%), followed by a moderate effect against *Candida albicans* (36 ± 3%). No relevant activity was detected against the Gram-negative bacterium *Escherichia coli* [21].

Additionally, the water extract of *M. germanica* collected in Turkey demonstrated in vitro antimicrobial activity, as inhibition of the growth of *Bacillus megaterium*, *Klebsiella pneumoniae*, *Candida glabrata*, and *Candida tropicalis* (inhibition zone ranging from 8 to 18 mm, with the highest activity against *C. tropicalis*), has been observed; however, with no activity against *E. coli*, *S. aureus*, *Proteus vulgaris*, or *C. albicans* [132]. In another investigation, the antimicrobial potential of methanolic extracts of *M. germanica* collected in India was assessed using a panel of Gram-positive and Gram-negative bacteria as well as fungi. The extract exhibited notable inhibitory effects, particularly against *S. aureus*, *Bacillus subtilis*, and *S. epidermidis* (20–27 mm). Moderate inhibition was observed for *Pseudomonas aeruginosa* and *C. albicans* (17–18 mm), while *P*. *vulgaris* and *E. coli* were not inhibited. Although the extract was less effective than the reference antibiotic kanamycin (30 mm), the results indicate a broad-spectrum antibacterial profile, especially against Gram-positive strains [44].

Studies on *M. germanica* from China have likewise demonstrated notable antibacterial properties of its aqueous and alcoholic extracts. Using an in vitro filter paper dispersion method, the minimum inhibitory concentration against *E. coli* was not significant (70% for the aqueous extract and 50% for the alcoholic extract). However, for eight other bacterial strains (*B. subtilis*, *S. aureus*, *Clostridium* sp., *P. vulgaris*, *Shigella dysenteriae*, *B. megaterium*, *Sarcina maxima*, and *Tetracoccus* sp.), the MIC values were below 5%, indicating strong bacteriostatic activity [149].

##### Compound-Level Evidence

A gallic acid derivative, gallicin (methyl 3,4-*O*-dimethylgallate), isolated from *M. laxiflora*, showed antimicrobial activities against *S. aureus* (MIC = 5 mg/mL), *E. coli* (MIC = 10 mg/mL), and *Rhizopus* sp. (MIC = 5 mg/mL) [63].

#### 5.2.4. Cytotoxic Activity

A small number of studies were devoted to evaluating the effects of *Myricaria* extracts and isolated compounds on various aspects of cancer cell function.

##### Extract-Level Evidence

The crude ethanolic extract of *M. germanica* collected in Austria was tested in three tumor cell lines over a concentration range of 0.01–100 μg/mL and with doxorubicin as a positive control. The crude extract exhibited significant cytotoxic activity against prostate (PC-3), human liver (Huh-7), and breast (MCF-7) cancer cell lines, with IC_50_ values of 6.5, 2.85, and 0.2 μg/mL, respectively [54].

Methanolic extracts from aerial parts and roots of *M. germanica* collected in the Himalayan region of India were also evaluated for their inhibitory effects on human cancer cell lines. Using the sulphorhodamine B (SRB) assay, cultured THP1 (leukemia), A549 (lung), HCT15 (colon), HeLa (cervix), and PC3 (prostate) cell lines were exposed to 100 μg/mL of the extracts for 48 h. Both extracts reduced cell viability, with the most pronounced activity observed against leukemia (83%) and lung (40%) cancer cell lines for aerial parts, and against colon cancer cells (68%) for root extracts [44].

The *M. longifolia* collected in Mongolia also demonstrated notable cytotoxic potential. Aqueous extracts inhibited the proliferation of breast cancer MCF7 cells and elicited a mild inhibitory effect on liver carcinoma HepG2 cells. This cytotoxicity was further supported by perfusion experiments, in which the aqueous extract induced structural damage to isolated rat liver tissue [79]. In another study, aqueous extracts of *M. longifolia* and their fractions were analyzed for potential genotoxic or cytotoxic properties using a primary rat hepatocyte assay with or without proliferative stimulation by epidermal growth factor (EGF). The extract exhibited marked cytotoxicity, with necrosis becoming significant at concentrations ≥ 10 μg/mL and exceeding 30% cell death at 100 μg/mL. In the absence of EGF, apoptosis increased alongside necrosis, suggesting a pro-apoptotic effect that may be masked by growth factor stimulation. Fractionation revealed that cytotoxicity correlated with methanol content, with the 80% methanol fraction inducing the highest necrosis (~90%) [148].

##### Compound-Level Evidence

Tamarixellagic acid (a tannin) and tamgermanetin (a feruloyl-amide), isolated from cytotoxically active ethanolic extract from *M. germanica* collected in Austria, affected cell-cycle distribution in Huh-7 and MCF-7 cells after 24 h and decreased the non-proliferating G/G1 cell fraction from 65% to 57% [54]. Both compounds increased caspase-3 activity in Huh-7 cells by 154.5 and 175%, respectively. Furthermore, tamarixellagic acid and tamgermanetin reduced PARP enzyme activity by 63.4 and 67.9%. These findings indicate that both compounds not only promote apoptosis in tumor cells but also enhance their sensitivity to DNA-damaging agents [54]. Two pentacyclic triterpenoids, myricarin A and B, isolated from the stems of *M. paniculata* from China, did not show cytotoxic activities (IC_50_ > 10 μm/mL) against all tested human cell lines (Bel-7402, BGC823, HCT-8, A549, MCF-7) [64].

#### 5.2.5. AChE Inhibition

The inhibition of acetylcholinoesterase (AChE) by the 80% ethanolic extract, DCM, EA, and BuOH fractions, as well as isolated compounds from the branches of *M. bracteata*, was investigated by Gendaram [69]. The Ellman spectrophotometric method was used, with DTNB [5,5′-dithiobis-(2-nitrobenzoic acid)] serving as the chromogenic reagent. At a concentration of 1 mg/mL, the EA fraction inhibited AChE activity by more than 60%, whereas the positive control, eserine, exhibited 100% inhibition at 0.1 mg/mL. The nonpolar DCM fraction showed no inhibitory effect. The ethanolic extract displayed stronger activity than the aqueous residue of the methanolic extract, indicating that the active constituents are primarily located in the more polar EA and BuOH fractions [69]. Among the isolated constituents screened for AChE inhibition, only quercetin glycosides (quercetin-3-*O*-β-D-glucuronide, quercetin-3-*O*-β-D-glucopyranoside, and quercetin-3-*O*-α-L-rhamnopyranoside) demonstrated notable activity, each achieving > 50% inhibition at 0.5 mg/mL [69]. Their AChE inhibitory IC_50_ values are shown in Table 4.

It should be noted, however, that studies based solely on the use of Ellman’s reagent may be prone to bias, as this test often yields false-positive results. Thus, the presented findings should be verified in the future using other methods, such as isothermal titration calorimetry [150].

#### 5.2.6. Immunomodulatory Effects

First evidence for the immunomodulatory properties of *M. germanica* comes from a study by Zeng et al. [147], which evaluated the effects of its water-soluble components in mice. The extract significantly increased the thymus and spleen indices (*p* < 0.05), enhanced the phagocytic activity of mononuclear macrophages, and overall improved immune function [144].

Subsequent studies were performed on isolated compounds, especially on a polysaccharide of *M. germanica* (MGP) isolated from plants grown in China, which has been reported as safe and non-toxic [75,94]. In the study by Wang et al., significant immunomodulatory activity of isolated MGP incorporated into chitosan–gold nanoparticle complexes (CS–Au–MGP NPs) was demonstrated. In vivo experiments in mice co-immunized marked enhancement of both humoral and cellular immune responses. The nanoparticles significantly increased thymus, spleen, and liver indices; elevated serum immunoglobulins IgG1 and IgG2a; and promoted cytokine release (IFN-γ and IL-6). In addition, CS–Au–MGP NPs stimulated splenic T and B lymphocyte proliferation, increased CD3^+^, CD4^+^, and CD8^+^ T-cell populations, and improved the CD4^+^/CD8^+^ ratio. Mechanistic analysis confirmed activation of the TLR2/IRAK4 signaling pathway, indicating stimulation of both innate and adaptive immunity. Histopathological examinations further showed that CS–Au–MGP NPs enhanced immune function across all tested doses without inducing detectable tissue damage [75].

Another study evaluated selenide-modified *M. germanica* polysaccharides (sMGP) encapsulated in poly (lactic-coglycolic-acid) (PLGA) nanoparticles as potential adjuvants for a Newcastle disease vaccine. sMGP-PLGA nanoparticles significantly enhanced phagocytic activity of RAW264.7 macrophages, upregulated CD40 and CD86 expression, and increased production of IFN-γ and IL-4, particularly within the concentration range of 3.125–25 µg/mL (*p* < 0.05). In vivo, the formulation stimulated immune activity in the spleen, thymus, and bursa of Fabricius in chickens, improving immune organ indices [94].

In a separate investigation, the immunoregulatory effects of total flavonoids from *M. germanica* auct. non Linn. Desv. administered intragastrically for two weeks at 1.0–1.5 g/kg were assessed in the FCA-induced arthritic rat model. The treatment reduced thymus index and TNF-α levels and improved lymphocyte transformation in the spleen, functional responses of peritoneal macrophages, and IL-2 content, suggesting immunomodulatory and anti-inflammatory potential [12].

#### 5.2.7. Choleretic Activity

The choleretic properties of extracts from several plants commonly used in Tibetan prescriptions for hepatobiliary disorders were evaluated, including the water extract of aerial parts of *Myricaria davurica*. Choleretic activity was assessed in vivo based on bile secretion rate (mg/min per 100 g body weight), total bile output over 4–5 h, and concentrations of major bile components (bile acids, cholesterol, and bilirubin). Decoctions were administered at doses of 0.01–1.0 g/kg (calculated from air-dried plant material). The intensity of bile secretion in rats of the experimental groups was evaluated by comparison with the data in the corresponding control group when the animals received an equivalent volume of purified water. In rats treated with *M. davurica* decoction at 0.05 g/kg, bile secretion increased by 18%, with only moderate enhancement in subsequent hours, and other tested parameters were compared to the control levels. According to the authors, the widespread use of moderately active plants in Tibetan formulations for liver and biliary tract diseases may be attributed to the complementary properties of their extracts rather than high individual potency [144].

#### 5.2.8. Pancreatic Lipase Inhibitory Activity

The lipid-lowering activity of extracts, fractions, and isolated compounds from the branches of *Myricaria bracteata* was studied by Gendaram [69]. Pancreatic lipase inhibitory activity was measured using a spectrophotometric assay, and orlistat served as a positive control. Among all isolated compounds tested, only tellimagrandin, an ellagitannin derivative, showed significant inhibitory activity, with an IC_50_ value of 0.051 ± 0.0001 mM, compared with 0.109 mM for orlistat. This indicates that tellimagrandin exhibits stronger pancreatic lipase inhibition than the reference drug [69].

#### 5.2.9. Prolyl Endopeptidase Inhibitory Activity

In a recent study, Ullah et al. reported a new class of prolyl endopeptidase inhibitors (PREPi) derived from the leaves of *Myricaria germanica *[73]. PREP is a serine protease responsible for the hydrolysis of regulatory polypeptides, including neuropeptides and substance P, and plays an important role in the progression of liver steatosis, contributing to metabolic dysfunction-associated fatty liver disease. Through combined isolation, bioactivity screening, computational analysis (molecular docking and simulations), and in vitro assays, the authors identified (±)-2-pentacosylcyclohexanol (PREPi) as a potent competitive inhibitor, with an IC_50_ value of 20.05 ± 1.6 µM, whereas the other isolated compounds (methyl gallate, syringic acid, and butanedioic acid) showed weaker activity. PREPi demonstrated pronounced hepatoprotective effects. In HepG2 cells exposed to palmitic acid-induced lipotoxicity, PREPi reduced oxidative stress, triglyceride accumulation, and expression of lipogenic genes. Furthermore, in a high-fat diet mouse model, PREPi markedly improved liver histology, lowered serum transaminase levels, and enhanced glucose and lipid metabolism. These findings indicate that *M. germanica* is a valuable source of bioactive metabolites with significant anti-inflammatory, antioxidant, and metabolic regulatory properties relevant to liver disease therapy [74].

#### 5.2.10. Glucose Absorption Inhibition

In a study evaluating the effect of Mongolian plant extracts on glucose absorption, *Myricaria bracteata* demonstrated inhibitory activity. Oral administration of hot water extract to rats significantly decreased peak blood glucose levels during the oral glucose tolerance test, as well as the net incremental area under the curve (net AUC). The authors suggested that this glucose-modulating activity may be associated with the high content of flavonols, flavanones, and tannins present in the active plants, as these compound classes are known to inhibit αglucosidase and aldose reductase [100].

#### 5.2.11. UVB-Protective Effect

Recent studies on *Myricaria paniculata* have demonstrated its protective effects against UVB-induced damage in HaCaT keratinocytes. Seven isolated compounds (kaempferol, rhamnazin, rhamnocitrin, quercetin-3-*O*-β-D-glucuronic acid, gallic acid, caffeic acid, ferulic acid) improved cell viability, inhibited apoptosis, and modulated oxidative stress markers by reducing ROS and MDA levels while enhancing SOD activity. Furthermore, these compounds significantly reduced pro-inflammatory cytokines (IL-6 and TNF-α) and caspase-3 expression, indicating both anti-inflammatory and anti-apoptotic properties. Network pharmacology and molecular docking analyses revealed interactions with key targets, including TNF, PTGS2, EGFR, and MMP9, suggesting that *M. paniculata* exerts its effects through multi-target and multi-pathway mechanisms, primarily involving antioxidant and inflammatory signaling pathways [92].

#### 5.2.12. Anti-Complementary Activity

The anti-complementary activity of different extracts and fractions of *Myricaria wardii* collected in the Tibetan region of China was assessed using hemolysis inhibition assays. The half-inhibit hemolysis concentration (CH_50_) values ranged from 5.86 ± 0.12 μg/mL to 21.57 ± 0.29 μg/mL, with EA and PA fractions displaying activity most comparable to heparin [45].

#### 5.2.13. Toxicity

Acute toxicity testing demonstrated a broad safety margin for water-soluble components of *Myricaria germanica* in mice, with an oral maximum tolerance of 120 g/kg and an intraperitoneal LD_50_ of 4.45 ± 0.03 g/kg (confidence limit: 3.83–5.16 g/kg). These findings confirm that *M. germanica* exhibits low toxicity [147]. On the other hand, the cytotoxic effect of the aqueous extract of *M. longifolia* (aerial parts) induced structural damage in isolated rat liver tissue during perfusion experiments [79,104].

### 5.3. Non-Therapeutic Use

Plants of the genus *Myricaria* spp. are used not only in traditional medicine or as ornamental plants (see Section 5.1 and Section 3.3, respectively), but also for utilitarian purposes. Several species were collected as fuelwood, for example, *M. germanica* in Europe and the Indian mountains, *M. bracteata* in the western Himalayas, *M. rosea* in Nepal, and *M. squamosa* in Afghanistan [122,149,150]. In some regions, *M. rosea* has also been used as an incense [151,152].

The bark (including the root bark) and other plant parts of the false tamarisk species provide black dye. The high tannin content also enabled its use in leather tanning [7,98].

Due to their flexible branches, some *Myricaria* species (such as *M. laxiflora*) are used for fencing and weaving [153]. The branches of *M. germanica* were used in basketry in the Balkans [133], while those of *M. squamosa* served as material for yurt screen doors among communities in northeast Afghanistan [123]. The uses of *Myricaria* species also extend beyond their decorative appeal. These plants can also be used as pioneer plants on gravel pits or industrial waste heaps, stabilizing the soil and forming attractive thickets. Efforts are also being made to employ them in areas contaminated with heavy metals or affected by salinization [154].

## 6. Conclusions and Future Perspectives

*Myricaria* Desv. is a small genus of the Tamaricaceae family with significant potential not only in terms of its ornamental value. Its pink, white, or purplish-red flowers, arranged on spike-like inflorescences, are very decorative and could definitely be considered as an alternative to the more widely known *Tamarix* species. Other benefits include their winter resistance, high growth rate, and relatively low cultivation requirements. Many of *Myricaria* spp. represent pioneer plants that grow on sandy or pebble soils in places where no other or few plant organisms are able to survive.

Independent of their decorative value, the *Myricaria* species represent an interesting example of plants with a rich and unique phytochemical profile. Based on the available data, it can be stated that, generally, the aerial parts of *Myricaria* species are rich in biologically active compounds, including flavonoids, phenolic acids, and oligomeric catechin derivatives. Plants from this genus, as well as other Tamaricaeae plants, also contain triterpenes, including the characteristic myricarins. However, little is known about the phytochemical profile of the underground parts of *Myricaria* species. The most intriguing are the sulfated flavonoid derivatives (mono- and disulfates), while other, more rare phytochemical constituents include feruloyl-amides. These compounds are as compelling as they are under-researched in terms of their pharmacological properties, and their contribution to the activity of the examined extracts has not yet been determined.

Any in-depth metabolomic comparison is challenging due to gaps in the data describing the majority of the *Myricaria* species. Several taxa remain poorly investigated, particularly the narrow endemics occurring in China, some of which have not been examined phytochemically or pharmacologically at all. In contrast, species with a broad geographic distribution—most notably the only European representative *M. germanica*, as well as *M. longifolia*, and especially *M. bracteata* from southern Siberia and western to southern Asia—have been the subject of considerably more extensive research. The last two hold additional ethnopharmacological relevance, as they are also used in Mongolian and Tibetan traditional medicine.

Antioxidant, anti-inflammatory, analgesic, cytotoxic, and antibacterial activity have been confirmed in scientific studies on individual *Myricaria* species. To date, anti-inflammatory activity has been the best documented. This may be explained by the presence in *Myricaria* species of a relatively large pool of polyphenolic compounds, including flavonoids. The effect was demonstrated in vivo, supported by mechanistic evidence showing reduced levels of pro-inflammatory cytokines and suppressed inflammatory pathways. Nevertheless, due to the still relatively limited number of studies on the pharmacological properties of species from the genus *Myricaria*, it is not possible to clearly assess the medicinal potential of these plants. The results of existing studies are difficult to compare, as extracts have often been prepared from different parts of the plants (e.g., leaves, twigs, whole plant) or using different methods (e.g., solvent, temperature, time of extraction) and have not been characterized in terms of their quantitative phytochemical profiles. Moreover, many studies cited in this review exhibit methodological shortcomings, such as the absence of reference standards. As a result, drawing unequivocal conclusions or conducting meaningful cross-species comparisons is not currently possible.

What is more, several studies included in this review report the activity of isolated compounds. However, these are primarily structures that are very common in the plant kingdom, with only a few examples of rare metabolites characteristic of the genus *Myricaria* or, more broadly, the family Tamaricaceae (e.g., myricarins, tamarixinin A, tamarixetin). Furthermore, due to the lack of available quantitative studies on the content of various metabolites in individual plants and extracts, it can only be assumed that the effective doses observed in experimental studies for isolated compounds would not be achievable through the use of whole mixtures (extracts or fractions). Nevertheless, investigating potential synergistic effects among the components of these mixtures would be a promising research direction. However, this would require studies that are properly designed from both phytochemical and pharmacological perspectives.

Another difficulty in making any definitive statements is the fact that the individual *Myricaria* species are polymorphic and morphologically similar, which makes them difficult to distinguish. Interpreting the available phytochemical and ethnopharmacological data is further complicated by the still unresolved taxonomy of the genus. Therefore, future studies should place special emphasis on the accurate identification of plant material. In this context, an integrative taxonomic approach is strongly recommended, combining classical morphological analyses with complementary methods such as micromorphological characterization, molecular techniques, and biochemical or chemotaxonomic studies, including secondary metabolite profiling and metabolomics. The application of such multidisciplinary approaches can significantly improve the discrimination of closely related taxa and enhance the reliability and reproducibility of phytochemical and pharmacological investigations.

Figure 15 provides an integrative schematic linking species, key metabolite classes, traditional uses, and major reported bioactivities, summarizing the key findings in the review.

## Figures and Tables

**Table 2:** Chemical compounds identified in *Myricaria* species.

Species	Plant Parts	Compounds	Method of Identification	References
*M. bracteata*	aerial parts ^1^	*Flavonoids*		[47,49,57,59,62,69]
		Kaempferol	UV, NMR; n.g. ^2^	
		Kaempferide	UV	
		Rhamnocitrin	n.g. ^2^	
		Afzelin	UV, NMR	
		Kaempferol-3-*O*-β-D-glucuronide	“	
		Kaempferol-3-*O*-β-D-glucuronic acid methylester	n.g. ^2^	
		Quercetin	UV, IR; NMR	
		Isorhamnetin	“	
		Rhamnetin	UV, IR	
		Rhamnazin	“	
		Isoquercetin	UV, NMR	
		Quercitrin	“	
		Tamarixetin	UV	
		Quercetin-3-*O*-β-D-glucuronide	UV, NMR	
		Quercetin-3-*O*-β-D-glucuronic acid methylester	“	
		Tamarixetin-6-desoxyhexose	UV	
		Chrysoeriol	n.g. ^2^	
		*Tannins*		[50,51,61,69]
		combined ellagotannins: dehydrodigallic acid, dehydrotrigallic acid	IR, NMR	
		Myrinin (1,2,3-dehydrotrigalloyl-α-D-glucose)	“	
		Nilotinin M4	NMR	
		1,3-di-*O*-Galloyl-4,6-*O*-(*aS*)-hexahydroxydiphenoyl-β-D-glucose	“	
		Tellimagrandin II	“	
		Bracteatinin D_1_ (dimer)	“	
		Bracteatinin D_2_ (dimer)	“	
		Tamarixinin A (dimer)	“	
		Nilotinin D8 (dimer)	“	
		Hirtellin A (dimer)	“	
		Hirtellin B (dimer)	“	
		Hirtellin E (dimer)	“	
		Isohirtellin C (dimer)	“	
		Bracteatinin T_1_ (trimer)	“	
		Hirtellin T_3_ (trimer)	“	
		*Phenolic acids and their derivatives*		[48,57,59,62,69]
		Gallic acid	UV, NMR	
		Ethyl gallate	UV, NMR	
		Methyl 3,4-*O*-dimethylgallate (gallicin)	n.g. ^2^	
		Methyl 3,4-dihydroxy-5-methoxybenzoate	IR, elemen. anal.	
		3, 5-Dihydroxy-4-methoxybenzoic acid	n.g. ^2^	
		Ellagic acid 3,3′,4-trimethylether	n.g. ^2^	
		Ellagic acid 3,3′-dimethylether	n.g. ^2^	
		Ferulic acid	UV, NMR, n.g. ^2^	
		Caffeic acid	n.g. ^2^	
		Syringaresinol	n.g. ^2^	
		(-)-Lyoniresinol	n.g. ^2^	
		(-)-Isolariciresinol	n.g. ^2^	
		*Feruloyl-amides*		[62]
		N-*trans*-Feruloyltyramine	n.g. ^2^	
		N-*trans*-Feruloyl-3-methoxytyramine	n.g. ^2^	
		N-*trans*-Feruloyl-2′-methoxytyramine	n.g. ^2^	
		*Sterols*		[57,59,69]
		β-Sitosterol	UV, NMR; n.g. ^2^	
		β-Sitosterylglucopyranose	“	
		Daucosterol	n.g. ^2^	
		*Long-chain fatty alcohols*		[46]
		12-Hentriacontanol	TLC, IR	
		*Other compounds*		[59,69,70]
		Palmitic acid	GLC	
		Stearic acid	UV, NMR	
		Stearic acid ethylate	“	
		Oleic acid	GLC	
		Linoleic acid	“	
		6,7,10-Trihydroxy-8-octadecenoic acid	n.g. ^2^	
		Palmitic acid	n.g. ^2^	
		Hexadecanoic acid 2,3-dihydroxypropyl ester	n.g. ^2^	
		Vitamin C	titrimetic method	
		Vitamin A, E	fluorimetric method	
		Fe, K	AAS	
	leaves	*Flavonoids*		[55,56]
		Kaempferol	LC-MS/MS	
		Kaempferide	“	
		Rhamnocitrin	“	
		Astragalin	“	
		Quercetin	“	
		Isorhamnetin	“	
		Rhamnazin	“	
		Isoquercetin	“	
		Hyperoside	“	
		Narcissin	“	
		Myricetin	“	
		Apigenin	“	
		Luteolin	“	
		Naringenin	“	
		Chrysoeriol	“	
		*Phenolic acids and their derivatives*		
		Gallic acid	LC-MS/MS	
		Methyl gallate	“	
		Ethyl gallate	“	
		Ellagic acid	“	
		Ferulic acid	“	
		*Other compounds*		
		Citric acid	LC-MS/MS	
	root bark	*Flavonoids*		[53]
		Afzelin	UV (CZE)	
		*Phenolic acids and their derivatives*		[53]
		Gallic acid	UV (CZE)	
		3,4-Dimethoxygallic acid	“	
		Gallic acetate	“	
	n.i.	*Flavonoids*		[66]
		Rhamnazin	n.g. ^2^	
		Dillenetin	n.g. ^2^	
		Rhamnocitrin	n.g. ^2^	
		*Phenolic acids and their derivatives*		[66]
		Methyl 3,5-dihydroxy-4-methoxybenzoate	n.g. ^2^	
		Methyl *p*-hydroxybenzoate	n.g. ^2^	
		3,3′,4′-Trimethoxyellagic acid	n.g. ^2^	
		Isoferulic acid	n.g. ^2^	
		*trans*-Ferulic acid 22-hydroxydocosanoic acid ester	n.g. ^2^	
		Docosyl-3,4-dihydroxy-*trans*-cinnamate	n.g. ^2^	
		*Other phenolics*		[66]
		Sinapaldehyde	n.g. ^2^	
		Vanillin	n.g. ^2^	
		Syringaldehyde	n.g. ^2^	
		*Triterpenoids*		[66]
		Myricarin	n.g. ^2^	
		Myricarin B	n.g. ^2^	
		3-α-Hydroxytaraxer-14-en-28-oic acid	n.g. ^2^	
		Myricadiol	n.g. ^2^	
*M. davurica*	n.i.	*Phenolic acids and their derivatives*		[71]
		Ellagic acid	n.g.	
*M. germanica*	leaves	*Flavonoids*		[43,54,72]
		Kaempferol	UV, FTMS, NMR, FAAS	
		Afzelin	“	
		Kaempferide	“	
		Kaempferol 3-*O*-β-D-glucuronide	“	
		Kaempferol 3-sulfate	n.g. ^2^	
		Kaempferol 7-sulfate	n.g. ^2^	
		Kaempferide 3,7-disodium sulfate	n.g. ^2^	
		Quercetin	UV, FTMS, NMR, FAAS	
		Quercitrin	“	
		Quercetin 3-*O*-β-D-glucuronide	“	
		Quercetin 3-sulfate	“	
		Quercetin 7-sulfate	n.g. ^2^	
		Tamarixetin	n.g. ^2^	
		Tamarixetin 3-*O*-β-glucuronide	UV, FTMS, NMR, FAAS	
		Tamarixetin 3-sodium sulfate	“	
		*Tannins*		[43,54]
		1,3-Di-*O*-galloyl-β-glucose	UV, FTMS, NMR, FAAS	
		2,3-Di-*O*-galloyl-(α/β)-glucose	“	
		2,4-Di-*O*-galloyl-(α/β)-glucose	“	
		2,6-Di-*O*-galloyl-(α/β)-glucose	“	
		Tamarixellagic acid	“	
		*Phenolic acids and their derivatives*		[43,54]
		Gallic acid	UV, FTMS, NMR, FAAS	
		3-*O*-Methylgallic acid	“	
		3-*O*-Methylgallic 5-sodium sulfate	“	
		Methyl gallate	GC-MS, NMR, IR	
		Syringic acid	“	
		*Feruloyl-amides*		[43,54]
		Tamgermanetin (N-*trans*-3-hydroxy-4-methoxycinnamoyltyramine)	UV, FTMS, NMR, FAAS	
		*Other compounds*		[73]
		Butanedioic acid	GC-MS, NMR	
		(±)-2-Pentacosylcyclohexanol	“	
	twigs	*Flavonoids*		[60]
		Kaempferol	n.g. ^2^	
		Rhamnocitrin	n.g. ^2^	
		Kaempferide	n.g. ^2^	
		Afzelin	n.g. ^2^	
		Kaempferol 3-*O*-β-D-glucuronide	n.g. ^2^	
		Rhamnazin	n.g. ^2^	
		Tamarixetin	n.g. ^2^	
		Quercitrin	n.g. ^2^	
		Quercetin 3-*O*-β-D-glucuronide	n.g. ^2^	
		Isoquercetin	n.g. ^2^	
		*Phenolic acids and their derivatives*		[74]
		Gallic acid	n.g. ^2^	
		4-*O*-Methylgallic acid	n.g. ^2^	
		Ferulic acid	n.g. ^2^	
		Caffeic acid	n.g. 2	
		*p*-Coumaric acid	n.g. ^2^	
		3,4,5-Trihydroxycinnamic acid	n.g. ^2^	
		Vanillic acid	n.g. ^2^	
		Feruloyl glucose	n.g. ^2^	
		*Other phenolics*		[74]
		Rhododendrol	n.g. ^2^	
		Coniferyl alcohol	n.g ^2^	
		(-)-Isolariciresinol	n.g. ^2^	
	leaf wax	*Long-chain fatty alcohols*		[68]
		Pentacosane-8,10-diol	GC-FID, GC-MS	
		Heptacosane-6,8-diol	“	
		Heptacosane-8,10-diol	“	
		Heptacosane-10,12-diol	“	
		Nonacosane-3,10-diol	“	
		Nonacosane-8,10-diol	“	
		Nonacosane-10,12-diol	“	
		Hentriacontane-2,12-diol	“	
		Hentriacontane-3,12-diol	“	
		Hentriacontane-4,12-diol	“	
		Hentriacontane-5,12-diol	“	
		Hentriacontane-6,12-diol	“	
		Hentriacontane-7,12-diol	“	
		Hentriacontane-8,12-diol	“	
		Hentriacontane-10,12-diol	“	
		Hentriacontane-12,13-diols	“	
		Hentriacontane-12,14-diol	“	
		Hentriacontane-12,15-diol	“	
		Hentriacontane-12,16-diol	“	
		Hentriacontane-12,17-diol	“	
		Hentriacontane-12,18-diol	“	
		Dotriacontane-1,9-diol	“	
		Dotriacontane-1,11-diol	“	
		Dotriacontane-1,13-diol	“	
		Tritriacontane-6,12-diol	“	
		Tritriacontane-8,10-diol	“	
		Tritriacontane-8,14-diol	“	
		Tritriacontane-10,12-diol	“	
		Tritriacontane-10,14-diol	“	
		Tritriacontane-12,16-diol	“	
		Tritriacontane-14,18-diol	“	
		Tetratriacontane-1,11-diol	“	
		Pentatriacontane-8,10-diol	“	
		Pentatriacontane-10,12-diol	“	
		Hexatriacontane-8,10-diol	“	
		Hexatriacontane-9,11-diol	“	
		Heptatriacontane-8,10-diol	“	
		Heptatriacontane-10,12-diol	“	
		Octatriacontane-8,10-diol	“	
		Octatriacontane-9,11-diol	“	
		Octatriacontane-10,12-diol	“	
		Nonatriacontane-8,10-diol	“	
		Nonatriacontane-8,11-diol	“	
		Nonatriacontane-10,12-diol	“	
		Tetracontane-8,10-diol	“	
		Tetracontane-9,11-diol	“	
		Tetracontane-10,12-diol	“	
		Hentetracontane-8,10-diol	“	
		Hentetracontane-8,11-diol	“	
		Hentetracontane-10,12-diol	“	
		Hentetracontane-10,13-diol	“	
		Tritetracontane-8,11-diol	“	
		Tritetracontane-10,13-diol	“	
	n.i.	*Other compounds*		[75]
		Polysaccharide	n.g.	
*M. germanica* auct. non Linn. Desv.	leaves/branches	*Flavonoids*		[13]
		Kaempferol	UPLC-Q-TOF MS/MS	
		Kaempferol-3-*O*-rutinoside	“	
		Isorhamnetin	“	
		Isoquercetin	“	
		Quercitrin	“	
		Rutoside	“	
		Quercetin 3-*O*-β-D-glucuronide	“	
		Apigenin	“	
		Acacetin	“	
		Genkwanin	“	
		Isovitexin	“	
		Eupafolin	“	
		Diosmetin	“	
		Homoorientin	“	
		Luteolin 7-*O*-glucuronide	“	
		Calycosin-7-*O*-β-D-glucoside	“	
		Jaceosidin	“	
		Eriodictyol	“	
		(+)-Catechin	“	
		*Tannins*		[13]
		Corilagin	UPLC-Q-TOF MS/MS	
		*Phenolic acids and their derivatives*		[13]
		4-Hydroxybenzoic acid	UPLC-Q-TOF MS/MS	
		5-Acetylsalicylic acid	"	
		4-Methoxysalicylic acid	“	
		Isovanillic acid	“	
		Gallic acid	“	
		Methyl gallate	“	
		Propyl gallate	“	
		Brevifolincarboxylic acid	“	
		Caffeic acid	“	
		Isoferulic acid	“	
		Ferulic acid	“	
		Phenethyl caffeate	“	
		Ethyl ferulate	“	
		1,3-Dicaffeoylquinic acid	“	
		*Other phenolics*		[13]
		Pyrogallol	UPLC-Q-TOF MS/MS	
		Vanillin	“	
		3,5-Dimethoxy-4-hydroxybenzaldehyde	“	
		(+)-Pinoresinol	“	
		Isoeugenol acetate	“	
		*Triterpenoids*		[13]
		Quillaic acid	UPLC-Q-TOF MS/MS	
		Corosolic acid	“	
		*Other compounds*		[13]
		Azelaic acid	UPLC-Q-TOF MS/MS	
		Aristolone	“	
		Abscisic acid	“	
		Germacrone	“	
		Isoalantolactone	“	
		Citric acid	“	
*M. laxiflora*	whole plant	*Phenolic acids and their derivatives*		[65]
		Methyl 3-*O*-methylgallate	ESI-MS, NMR	
		*Triterpenoids*		[65]
		Erythrodiol	ESI-MS, NMR	
		Myricadiol	“	
		Isomyricadiol	“	
		(5R, 8R, 9R, 10R, 13S, 17S, 18S) 25, 28-Dihydroxy-D-friedoolean-14-en-3-one	“	
		28-Hydroxy-D-friedoolean-14-en-3-one	“	
		3-α-Hydroxy-D-friedoolean-14-en-28-oic acid	“	
		3-α-[4″-Hydroxy-trans-cinnamoyloxy]-D-friedoolean-14-en-28-oic acid	“	
		*Sterols*		[65]
		β-Sitosterol	ESI-MS, NMR	
	n.i.	*Phenolic acids and their derivatives*		[63]
		Methyl 3,4-*O*-dimethylgallate (gallicin)	n.g.	
*M. longifolia*	leaves	*Flavonoids*		[20,56,76,77,78]
		Kaempferol	HPLC-DAD	
		Astragalin	HPLC-DAD, LC-MS	
		Quercetin	“	
		Rhamnetin	HPLC-UV-DAD, LC-MS	
		Isoquercetin	HPLC-DAD, LC-MS	
		Hyperoside	“	
		Avicularin	“	
		Quercetin 3-*O*-β-D-glucuronide	HPLC-DAD, UV, GC, NMR	
		Quercetin 3-sulfate	“	
		Narcissin	HPLC-DAD	
		Isorhamnetin 3-sulfate	HPLC-UV-DAD, LC-MS	
		Apigenin	HPLC-DAD, LC-MS	
		Luteolin	“	
		Naringenin	“	
		*Phenolic acids and their derivatives*		[20,56,76,77]
		Gallic acid	HPLC-DAD, LC-MS	
		Ellagic acid	“	
		Ferulic acid	“	
		Citric acid	“	
	twigs/overground parts	*Flavonoids*		[52,77]
		Kaempferol	HPLC-DAD	
		Quercetin	UV, HPLC-DAD	
		Isoquercetin	UV	
		Rhamnetin	“	
		Tamarixetin	“	
		Astragalin	HPLC-DAD	
		Hyperoside	“	
		Narcissin	“	
		Naringenin	“	
		*Phenolic acids and their derivatives*		[77]
		Gallic acid	HPLC-DAD	
		Ellagic acid	“	
		Ferulic acid	“	
	n.i.	*Flavonoids*		[79,80]
		Quercetin	HPLC-UV-DAD, LC-MS	
		Rhamnetin	“	
		Rutin	“	
		*Phenolic acids and their derivatives*		[79,80]
		Gallic acid	HPLC-UV-DAD, LC-MS	
		Ellagic acid	“	
		Isoferulic acid	“	
		Caffeic acid	“	
		Syringic acid	"	
		*Sterols*		[79]
		β-Sitosterol	HPLC-UV-DAD, LC-MS	
*M. paniculata*	stem	*Triterpenoids*		[64]
		*epi*-Friedelanol	HREIMS, IR	
		28-Hydroxy-D-friedoolean-14-en-3-one	“	
		Myriconal	“	
		Myricarin A	“	
		Myricarin B	“	
		*Sterols*		[64]
		β-Sitosterol	HREIMS, IR	
		4-Methylstigmast-7-en-3-ol	“	
		*Long-chain fatty alcohols*		[64]
		Triacontanol	HREIMS, IR	
		Hentriacontan-12-ol	“	
	n.i.	*Flavonoids*		[58]
		Morelloflavone	n.g. ^2^	
		*Phenolic acids and their derivatives*		[58]
		Methyl 4-*O*-methylgallate	n.g. ^2^	
		Isoferulic acid	n.g. ^2^	
		*Triterpenoids*		[58]
		*epi*-Friedelanol	n.g. ^2^	
		28-Hydroxy-D-friedoolean-14-en-3-one	n.g. ^2^	
		28-Aldehyde-taraxerenone	n.g. ^2^	
		*Sterols*		[58]
		4-Methylstigmast-7-en-3-ol	n.g. ^2^	
*M. squamosa*	overground parts	*Triterpenoids*		[67]
		3-α-Hydroxy-D-friedoolean-14-en-28-oic acid	IR, NMR, HR-ESI-MS	
		Myricarin A	“	
		Myricarin B	“	
		Myricarin C	“	
	n.i.	*Flavonoids*		[81]
		Rhamnazin	n.g. ^2^	
		Isoquercetin	n.g. ^2^	
		Afzelin	n.g. ^2^	
		*Phenolic acids and their derivatives*		[81]
		2,3,8-Tri-*O*-methylellagic acid	n.g. ^2^“	
		*Other phenolics*		[81]
		Syringenin	n.g. ^2^	
		*Triterpenoids*		[81]
		Isomyricadiol	n.g. ^2^	
		*Other compounds*		[81]
		Methyl linolenate	n.g. ^2^	
*M. wardii*	leafy twigs/branches ^3^	*Flavonoids*		[45]
		Quercetin	UPLC-Q-TOF-MS/MS	
		Afzelin	“	
		Isoquercetin	“	
		Quercitrin	“	
		Hyperoside	“	
		Apigenin	“	
		Luteolin	“	
		Tricin	“	
		Taxifolin	“	
		Phlorizin	“	
		*Phenolic acids and their derivatives*		[45]
		Protocatechuic acid	UPLC-Q-TOF-MS/MS	
		Vanillic acid	“	
		Methyl vanillate	“	
		Gallic acid	“	
		3-*O*-Methylgallic acid	“	
		3,4-*O*-Dimethylgallic acid	“	
		Syringic acid	“	
		Methyl 3-*O*-methylgallate	“	
		Ellagic acid	“	
		Isoferulic acid	“	
		Caffeic acid	“	
		3,4-Dimethoxycinnamic acid	“	
		*Feruloyl-amides*		[45]
		N-Feruloyl tyramine	UPLC-Q-TOF-MS/MS	
		Isomer-N-feruloyl tyramine	“	
		3-(4-Hydroxy-3-methoxyphenyl)-N-(2-(4-hydroxyphenyl)-2-methoxyethyl)-acrylamide	“	
		*Other phenolics*		[45]
		Protocatechualdehyde	UPLC-Q-TOF-MS/MS	
		Coniferylaldehyde	“	
		*Triterpenoids*		[45]
		Myricarin A	UPLC-Q-TOF-MS/MS	
		*Other compounds*		[45]
		8,11,12-Trihydroxy-9-octadecenoic acid	UPLC-Q-TOF-MS/MS	
		Linoleic acid	“	
		Aesculetin	“	
		Effusol	“	
	leafy twigs ^3^	*Flavonoids*		[45]
		Kaempferol	UPLC-Q-TOF-MS/MS	
		*Phenolic acids and their derivatives*		[45]
		Methyl 4-*O*-methylgallate	UPLC-Q-TOF-MS/MS	
		*p*-Coumaric acid	“	
		*Feruloyl-amides*		[45]
		N-Feruloyl-3-methyldopamine	UPLC-Q-TOF-MS/MS	
		*Other compounds*		[45]
		Hexadecenoic acid	UPLC-Q-TOF-MS/MS	
		9,12,13-Trihydroxy-10,15-octadecadienoic acid	“	
	branches ^3^	*Flavonoids*		[45]
		Diosmetin	UPLC-Q-TOF-MS/MS	
		Dihydrokaempferol	“	
		*Phenolic acids and their derivatives*		[45]
		Methyl 3,4-*O*-dimethylgallate (gallicin)	UPLC-Q-TOF-MS/MS	
		Vanillin	“	
		Terrestriamide	“	
		*Triterpenoids*		[45]
		Ursolic acid	UPLC-Q-TOF-MS/MS	
		Corosolic acid	“	
		Myristic acid	“	
	root bark	*Flavonoids*		[53]
		Rhamnetin	UV (CZE)	
		Afzelin	“	
		*Phenolic acids and their derivatives*		[53]
		Gallic acid	UV (CZE)	
		3,4-Dimethylgallic acid	“	

n.i.—not indicated; n.g.—not given; “—same method as above. ^1^ Described by authors as: epigeal parts, aerial parts, herb, stems, twigs, branches, leaves and branches. ^2^ Chromatographic isolation and identification by spectroscopic method. No details were given in the abstract. ^3^ The authors difference the compounds’ content in leafy twigs, branches, and both leafy twigs and branches. Abbreviations: AAS—Atomic Absorption Spectrometry; elemen. anal—Elementary Analysis; CZE—Capillary Zone Electrophoresis; ESI-MS—Electrospray Ionisation Mass Spectrometry; FAAS—Flame Atomic Absorption Spectroscopy; GC—Gas Chromatography; GC-FID—Gas Chromatography with Flame Ionization Detector; GLC—Gas–Liquid Chromatography; GC-MS—Gas Chromatography-Mass Spectrometry; HPLC—High Performance Liquid Chromatography; HPLC-UV-DAD—High-Performance Liquid Chromatography with UV-Diode Array Detector; HREIMS—High-Resolution Electron Ionization Mass Spectrometry; HR-ESI-MS—High-Resolution Electrospray Ionization Mass Spectrometry; IR—Infrared spectroscopy; LC-MS—Liquid Chromatography-Mass Spectrometry; LC-MS/MS—Liquid Chromatography Tandem Mass Spectrometry; NMR—Nuclear Magnetic Resonance; FTMS—Fourier Transform Mass Spectrometry; TLC—Thin-Layer Chromatography; UPLC-Q-TOF-MS/MS—Ultra-Performance Liquid Chromatography combined with Quadrupole Time-of-Flight Tandem Mass Spectrometry; UV—Ultraviolet Spectroscopy.

**Table 3:** Traditional uses of *Myricaria* species.

Species	Traditional Names	Traditional Uses	References
SIBERIA, CENTRAL ASIA, AND MONGOLIA			
*Myricaria bracteata*	Mirikariya lisokhvostnaya (Russian) Tsetsgiin dagavart balgana (Mongolian)	-Shoots: rheumatism, fever, phthisis, measles, chronic ulcers, rashes, boils, spasm, and atrophy; -Cortex: fever, diabetes, cytotoxic; -Aerial parts as ingredients of the traditional Mongolian formulas: Braivu-3, Gagol-18, Gontog-7, Hachgurum-25, Degd-13, and Debao-9 cure fever, counteract toxicity; -*Myricariae Ramulus* ingredient of the traditional Mongolian formula Ga Gu La-19 Powder: strengthens the spleen, curing spleen cold and spleen heat.	[69,99,100]
*M. davurica*	Mirikariya daurskaya (Russian) Om-bu (Tibetan)	-Respiratory infections; -Leaves: astringent-flavored; air, phlegm, and bile disease.	[101,102]
*M. longifolia*	Mirikariya dlinnolistnaya (Russian) Urt navchit balgana (Mongolian) Ombu (Tibetan)	-Shoots: the taste is sour and sweet, astringent, the potency is blunt and cool; treats fever and poisoning, counteracts toxicity; -Ingredient of the traditional Mongolian formulas: Braivu-3, Gagol-18, Gontog-7, Khach gurgum-25, Degd-13, and Dedbo-10, Debao-9, Hachgurum-25; -Ornamental, useful plant.	[99,103,104]
HIMALAYAS, TIBETAN PLATEAU			
*M. albiflora*		Blood purifier	[105]
*M. bracteata*	Wenbu, Om-bu (Tibetan) Hambu, Hombuk (India) Kuan bao shui bai zhi (Chinese)	-Aerial parts decoction: blood purifier, neutralizes poison (meat poisoning), rheumatism, arthritis; -Whole plant in rheumatism (Uttaranchal, India); -Fodder, fuel, and small timber.	[61,66,106,107]
*M. laxiflora*	Shu hua shui bai zhi (Chinese)	-Aerial parts/dry young branch: the taste is spicy and sweet; scald, fistula, scabies, alopecia areata, typhoid, arthritis, sprains, women’s leucorrhea, thromboangiitis obliterans.	[14,63]
*M. paniculata*	Shui bai zhi (Chinese)	-Twigs: traditional Tibetan herb, the taste is sour and sweet, cold in nature, clears heat and toxic material, dispels mild wind and relieves exterior syndrome, promotes eruption and relieves coughs; -Also to cure rheumatism and arthritis.	[64,108]
*M. platyphylla*		-Aerial parts as ingredients of traditional medicinal bath therapies (WuWei GanLu).	[109]
*M. prostrata*	Hom.bu, Chhu.shing.hom.bu, Ong bu (Tibetan)	-Aerial parts: neutralize poison (compounded or meat poison), bile fevers, pneumonia, also in veterinary; -External application for sores; -Whole plant as firewood/fuel.	[110,111]
*M. pulcherrima*		No data	
*M. rosea*	Umbu (India) Chu-sching-om-bu (chushing-om-bu), Wombur (Bhutan) Angmeo, Wonbu, Hanmbu, Humpu, Yumbu (Nepal) Wo sheng shui bai zhi (Chinese)	-Potency: sweet (ngar) and astringent (ka)/cool (sil); -Aerial parts: fever associated with poisoning (dug-tshad), meat poisoning (sha-dug), blood infection (khrag-tshad), diarrhea, stomachache, uterine bleeding, fever, dropsy, wounds, chicken pox; -Leaves, stems, and flowers in wated decoction or plant paste: orally in respiratory/lung diseases, asthma, cough, cold, headache, diarrhea;	[107,109,112,113,114,115,116,117]
		-Externally: relieves backache (plant paste), medicinal herbal bath; -Leaves, stems, and flowers in a water decoction for respiratory disease, asthma, bronchitis, breathing difficulty, 2–3 times a day (Manang and Mustang districts, Nepal); -Decoction for livestock in respiratory diseases (Manang district, Nepal); -Fuelwood and incense.	
*M. squamosa*	Onbu (Pakistan) Wombu (Nepal) ‘Om-bu, umbo (Tibetan) Tark, bölghön (Wakhi, Kyrgyz; Afghanistan)	-Whole plant: traditional Tibetan herb, has astringent taste and a cooling potency; blood fever, exterior syndrome, and aconitum poisoning; -Febrifuge, poison plant (Ladakh, India); -Flower and leaves: fever, headache, antidote to food and meat poisoning (Dolpo, Nepal); -Flower infusion: antitussive, febrifuge (Gilgit-Baltistan, Pakistan); -Neutralize the poison (meat poisoning); -Powder of flowers and leaves dusted on wounds, injuries (also for livestock) (Gilgit-Baltistan, Pakistan); -Branches: passed quickly through fire, the oil exuded is applied to ‘white skin’ (Wakhi people, Afghanistan); -Useful plant: for screen-door of yurts (Wakhi people, Afghanistan); fuelwood (Kirgiz nomads, Afghanistan).	[67,112,118,119,120,121,122,123]
*M. wardii*		-Shoots: Tibetan heat-clearing and detoxifying agent.	[45,124]
Myricariae ramulus (leafy twigs and branches of *Myricaria wardii*, *M. squamosa*, *M. paniculata*, *M. bracteata*, and *Myrtama elegans*)		-Twigs and branches: Tibetan heat-clearing and detoxifying agent; infections, sore throat, scalds, joint pain, rheumatic arthritis.	[45]
WESTERN HIMALAYA			
*M. germanica* (*M. germanica* subsp. *pakistanica*)	Hombug, humbu umbu, umbo, um-boo (India) Wengbu	Leaves: jaundice, chronic bronchitis, analgesic; Juice from fresh tender shoots with leaves: ingre-dient in the medicines to cure joint pains, swell-ings (Lahaul-Spiti region, India); Branches and leaves: cold, asthma, measles, scor-pion poison, limiting the effects of poison, rheu-matism; Bark decoction: jaundice, inflammation, sore throat; Leaves, stems, shoots or whole plant paste/decoction: applied to bruises and swollen joints, topical/oral toothache; Leaves: to treat jaundice, swollen joints, as aperi-ent, emollient (Uttaranchal); controls bronchitis, decoction once a day as a blood purifier (Ladakh, India); Fuel/fodder.	[106,107,115,125,126,127,128,129,130]
EUROPE			
*M. germanica*	Židoviník německý (Czech), Piskeris (Danish), German false tamarisk (English), Pensaskanevra (Finnish), Myricaire, tamarin, Tamarin d’Allemagne (French), Deutsche Tamariske, Rispelstrauch (Deutsch), Tamerici alpino (Italian), Klåved (Norvegian), Września (Polish), Klådris (Swedish), Myrikovka nemecká (Slovak); Herbakotu (Turkish)	-Infusion of leaves as analgesic; -Rubbing oil; -Useful plant: basketry; -Fuel/fodder.	[131,132,133]

**Table 4:** The results of pharmacological studies on *Myricaria* species.

*Myricaria* Species (Plant Part, Origin)	Tested Activity	Type of Extract or Tested Compounds	Results	Assay/Experimental Model	References
*M. bracteata*					
(twigs; collected in Qinghai, China)	antioxidant	isolated tannins: nilotinin M4, 1,3-di-*O*-galloyl-4,6-*O*-(*aS*)-hexahydroxydiphenoyl-β-D-glucose, bracteatinin D_1_, D_2,_ hirtellin A, B, E, isohirtellin C, tamarixinin A, nilotinin D8, bracteatynin T_4_, hirtellin T_3_	lipid peroxidation inhibition (↓MDA) IC_50_ [μg/mL]): - hirtellin A (15.6), hirtellin B (48.8), tamarixinin A (18.4)	in vitro: lipid peroxidation test; rat liver microsomes	[61]
			hydroxyl radical-scavenging activity (IC_50_ [μM]): - nilotinin M4 (37.96), 1,3-di-*O*-galloyl-4,6-*O*-(*aS*)-hexahydroxydiphenoyl-β-D-glucose (34.62), bracteatinin D_1_ (15.8), bracteatinin D_2_ (36.81), hirtellin A (38.49), hirtellin B (39.72), hirtellin E (16.27), isohirtellin C (42.91), tamarixinin A (41.52), nilotinin D8 (32.55), hirtellin T_3_ (55.1), bracteatinin D_1_ (15.8) - positive controls: BHT (22.63), Trolox (76.73), gallic acid (15.66)	hydroxyl radical-scavenging rate (modified deoxyribose degradation method)	
			DPPH radical-scavenging activity (IC_50_ [μM]): - all tested compounds (2.4 to 5.89) - positive controls: BHT (7.08), Trolox (8.0), gallic acid (7.23)	DPPH assay	
(branches; collected in Gobi-Altay, Mongolia)		EtOH, EA, BuOH fractions; isolated compounds: quercetin, quercetin 3-*O*-β-D-glucuronide, quercetin-3-*O*-β-D-glucopyranoside, kaempferol, isorhamnetin, gallic acid, ethyl gallate, tellimagrandin II	free-radical scavenging activity - extract/fractions IC_50_ [mg/mL]): EtOH (31.93 ± 0.48), EA (27.11 ± 0.58), BuOH (26.14 ± 0.31) - positive control: rutin (22.66 ± 0.29 mg/mL) - compounds (IC_50_ [μM]): quercetin (41.36 ± 0.89), quercetin 3-*O*-β-D-glucuronide (24.3 ± 0.27), quercetin-3-*O*-β-D-glucopyranoside (13.4 ± 1.04), kaempferol 86.7 ± 1.13), isorhamnetin (68.42 ± 0.02), tellimagrandin II (5.97 ± 0.52), gallic acid (30.9 ± 1.21), ethyl gallate (53.94 ± 1.2) - positive control: rutin (38.7 μM)	in vitro: DPPH assay	[69]
(twigs; collected in Qinghai, China)	anti-inflammatory	tamarixinin A	↑ viability rates; positive control: methotrexate	in vitro: viability rates (MTT assay); LPS-induced murine macrophages from C57BL/6J male mice	[61]
			% of inhibition rates [in sc mg/kg doses]: - ear edema (34.4 at dose 50.0 to 69.8 at dose 200)	in vivo: Croton oil-induced ear edema; ICR male mice	
			- paw edema 25.0 at dose 50	carrageenan-induced paw edema, ICR male mice	
			CIA in 56 days: 46.0 at dose 20.0	collagen-induced arthritis (CIA); DBA/1 mice	
			dose-dependent ↓ TNF-α, ↓ IL-6, ↓ NO; ↓ iNOS expression, ↓ MAPK and ↓NF-κB signal activation	in vitro: ELISA, Gres reagent, Western blot; peritoneal macrophages isolated from C57BL/6 mice	[142]
			effective dose: 12.5 mg/kg; ↓ paw swelling, ↓ body weight loss; ↓ IL-6, ↓ IL-1β	in vivo: CIA model; DBA/1 mice	
			effective dose: 6.25 mg/kg; ↓ paw swelling, ↓ erythrema; ↓ TNF-α, ↓ IL-1β; ↓ expression p38, p65, ↓phosphorylation p38; positive control: methotrexate	AIA model; Wistar rats	
(herb; collected in Gobi-Altay, Mongolia)	antimicrobial	crude 80% EtOH extract; DCM, EA, BuOH fractions	inhibition zone [mm] for dose 100 mg/mL: - EtOH: *S. aureus* (11.7), *E. faecalis* (12.2), *M. luteus* (13.7) - DCM: *S. aureus* (10.0), *M. luteus* (11.6) - EA: *P. aeruginosa* (10.5), *S. aureus* (14.6), *M. luteus* (14.6) - BuOH: *S. aureus* (12.7), *M. luteus* (11.8)	in vitro: disc diffusion method; bacteria: *P. aeruginosa*, *S. aureus*, *M. luteus*, *E. faecalis*, *E. coli*	[143]
(herb; collected in Novosibirsk, Russia)		volatile emission	relative decrease of the number of microorganism colonies [%]: *S. epidermis* (60–70), *C. albicans* (30–40), *E. coli* (0)	in vitro: bacteria: *S. epidermis*, *E. coli*, yeast: *C. albicans*	[21]
(branches; collected in Gobi-Altay, Mongolia)	AChE inhibition	crude 80% MeOH extract; DCM, EA, BuOH fractions, isolated compounds: quercetin-3-*O*-β-D-glucuronide, quercetin-3-*O*-β-D-glucopyranoside, quercetin-3-*O*-α-L-rhamnopyranoside	enzyme inhibition [IC_50_ [mM]): - in conc. 1 mg/mL: EA fraction (over 60%), other (40–59%) - positive control in conc. 0.1 mg/mL: physostigmine (0.000083) - quercetin-3-*O*-β-D-glucuronide (0.077 ± 0.002), quercetin-3-*O*-β-D-glucopyranoside (0.041 ± 0.001), quercetin-3-*O*-α-L-rhamnopyranoside (0.073 ± 0.005)	in vitro: Ellmann method (DTNB)	[69]
(branches; collected in Gobi-Altay, Mongolia)	pancreatic lipase inhibition	isolated compound: tallimagrandin II	enzyme inhibition (IC_50_ [mM]): - tellimagrandin II (0.051 ± 0.0001) - positive control: orlistat (0.109)	in vitro: DTNB assay	[69]
(cortex; n.i.)	glucose absorption inhibition	H_2_O extract	dose 500 mg/kg: ↓ intestinal glucose (2 g/kg b.w.) absorption	in vivo: intestinal glucose absorption; male Wistar rats	[100]
*M. davurica*					
(aerial parts; n.i.)	choleretic	decoction	at dose 0.05 g/kg: bile secretion rate in 5 h ↑18%	in vivo: Wistar rats	[144]
*M. germanica*					
(leaves; collected in Gilgit, Pakistan)	antioxidant	isolated compound: methyl gallate	viability [%]: - at dose 50 μM 95.5% ± 16.0 vs. 57.6% ± 1.1 (blank) - at dose 100 μM 85.5% ± 7.0 vs. 57.6% ± 1.1 (blank)	in vitro: protection of H_2_O_2_-induced oxidative stress apoptosis in β-cells; MTT assay; MIN6 cells	[145]
(aerial parts; collected in Quinghai, China)	anti-inflammatory	essential oil	skin inflammation ↓ TNF-α, ↓ IL-6, ↓ Caspase-3, ↓ ROS, ↓ MDA	in vitro: CCK-8 assay; HaCaT cells	[146]
			identified corresponding targets and signaling pathways	in silico: molecular docking and network pharmacology	
(leaves; collected in Turkey)	antimicrobial	infusion	inhibition zone [mm]: - *B. megaterium* (8), *K. pneumoniae* (8), *C. glabrata* (8), *C. tropicalis* (18) - other tested strains—no activity - positive controls: streptomycin sulfate (9–13), nystatin (11–18)	in vitro: disc-diffusion method; bacteria: *B. megaterium*, *P. aeruginosa*, *E. coli*, *K. pneumoniae*, *P. vulgaris*, *S. aureus*, yeasts: *C. albicans*, *C. glabrata*, *C. tropicalis*	[132]
(roots, aerial parts; collected in Jammu & Kashmir, India)		MeOH extract	inhibition zone [mm] in conc. 100 μg: *- P. aeruginosa* (17), *S. aureus* (25), *B. subtilis* (27), *S. epidermis* (20), *C. albicans* (18) - *P. vulgaris* and *E. coli* no activity - positive control: kanamycin (30)	in vitro: agar well diffusion method; bacteria: *P. aeruginosa*, *P. vulgaris*, *S. aureus*, *E. coli*, *B. subtilis*, *S. epidermis*, yeasts: *C. albicans*	[44]
(aerial parts, roots; collected in Jammu & Kashmir, India)	cytotoxic	MeOH extract	↓ viability [%] in dose 100 μg: aerial parts: THP-1 (83), A549 (40), HCT-15 (30), HeLa (22), PC-3 (24) - roots: HCT-15 (68), (other 3–16) THP-1 (16), PC-3 (15), A549 (13), HeLa (-3 - positive controls (in dose 1 × 10^−6^ μg) paclitaxel: THP-1 (13), A549 (61), HCT-15 (17), Hela (6) PC-3 (7); mitomycin-C: THP-1 (23), A549 (43); HCT-15 (21), Hela (4) PC-3 (67)	in vitro: SRB assay; human cancer cell lines: THP-1, A-549, HCT-15, HeLa, PC-3	[44]
(leaves; collected in Germany)		EtOH-H_2_O extract, isolated fractions with identified compounds	IC_50_ [μg/mL]: - crude extract: PC-3 (6.5), Huh-7 (2.85), MCF-7 (0.2) - active (better than control) fractions against PC-3: tamarixellagic acid (0.13), fraction contain quercetin 3-*O*-β-glucuronide, kaempferol 3-*O*-β-glucuronide, tamarixetin 3-*O*-β-glucuronide (0.22), 2,3-di-*O*-galloyl-(α/β)-glucose (0.3), 2,6-di-*O*-galloyl-(α/β)-glucose (0.4), fraction contain 1,3-di-*O*-galloyl-β-glucose, 2,4-di-*O*-galloyl-(α/β)-glucose (0.61) Huh-7: all fractions except fraction contain kaempferide 3-OSO_3_Na and tamarixetin 3-OSO_3_Na MCF-7: fraction contains gallic acid and 3-*O*-methylgallic acid (0.13), tamarixellagic acid (0.16) positive control: doxorubicin PC-3 (0.63), Huh-7 (1.5), MCF-7 (0.13) 2,6-di-*O*-galloyl-(α/β)-glucose (015) - fractions (tamarixellagic acid, tamgermanitin) act in cell cycle distribution at G_0_/G_1_, S, G_2_/M and pre-G-phase; ↑ caspase-3, ↓ cell-free PARP enzyme	in vitro: SRB assay, caspase-3 activity (Huh-7; Quantikine immunoassay kit), PARP enzyme activity assay	[54]
(n.i.; collected in Tibet, China)		nano-drug delivery system based on *M. germanica* polysaccharide nanoparticles complex	CS-Au-MGP nanoparticles: ↑ IgG1, ↑ IgG2, ↑ IFN-γ, ↑ IL-6 (+)thymus, spleen, and liver indices	in vivo: ELISA; ICR mice	[75]
(n.i.; collected in Tibet, China)		selenated polysaccharide isolated from leaves, PLGA encapsulated	↑ phagocytic ability ↑ IFN-γ, ↑ IL-4 ↑ organ index, (+)immune functions of spleen, thymus, and bursa of Fabricius	in vitro: RAW264.7 macrophages in vivo: MTT, ELISA; chicken	[94]
(leaves; collected in Gilgit-Baltistan, Pakistan)	prolyl endopeptidase (PREP) inhibition in model steatohepatitis	MeOH extract; EA, PE fractions; isolated compounds: (±)-2-pentacosylcyclohexanol, methyl gallate, syringic acid, butanedioic acid	PREP inhibition [%], (IC_50_ [μM]): - in conc. 1 mg/mL: EA (97.8%), - in conc. 500 mg/mL: (±)-2-pentacosylcyclohexanol (88%, 20.05 ± 1.6), methyl gallate (59.0%); syringic acid (75.4%, 155.13 ± 1.8), butanedioic acid (64.7%); ↑ cell viability	in vitro: ROS assay (fluorescence microscopy), TG assay kit, PCR; HepG2 cells	[73]
			protective effect (measured: TC, TG, LDL, HDL, ALT, AST); standard: inhibitor Kyp-2047	in vivo: cell viability assay, steatohepatitis model; male C57BL/6 mice	
			enzyme binding mechanism for (±)-2-pentacosylcyclohexanol	in silico: molecular docking	
(n.i.; n.i.)	toxicity	H_2_O extract	tolerance for dose 120 g/kg: LD_50_ 4.4482 ± 0.0329 g/kg (i.p.)	in vivo: mice	[147]
*Myricaria germanica* auct. non Linn. Desv.					
(branches and leaves; collected in Qinghai, China)	anti-inflammatory	MeOH extract, identified compounds	identified probably important targets and anti-RA activity pathways, proposed important active compounds: apigenin, isorhamnetin, quillaic acid	in silico: molecular docking and network pharmacology for determined compounds	[13]
(young branches; collected in Qinghai, China)	anti-inflammatory	flavonoid fraction	intragastric dose 1–1.5 g/kg: ↓ secondary inflammation from 24th day (paw swelling), ↓ thymus and spleen index, ↑ lymphocyte proliferation, ↑ phagocytosis of peritoneal macrophages, ↑ IL-2, ↑ TNF-α; positive control: indomethacin	in vivo: AA rats	[12]
*M. laxiflora*					
(n.i.; collected in China)	antimicrobial	isolated compound: gallicin	MIC [mg/mL]: - *S. aureus* (5), *E. coli* (10), *Rhizopus* sp. (5)	in vitro: bacteria: *S. aureus*, *E. coli*; fungus: *Rhysopus* sp.	[63]
*M. longifolia*					
(overground; collected in Mongolia)	hepatototoxic	H_2_O extract	dose-dependent effect: in concentration 100 μg/mL (more than 30% necrotic cells), no genotoxic effect	in vitro: primary rat hepatocyte assay with stimulation by EGF	[76,148]
(overground; collected in Mongolia)	anti-choleretic	H_2_O extract	bile flow [% of the basal value]: - in conc. 200 mg/L (−13) - in conc. 1000 mg/L (−51)	ex vivo: liver perfusion test; isolated rat liver	[76]
*M. paniculata*					
(twigs; n.i.)	antioxidant	H_2_O extract	scavenging activities [mg trolox/g dw]: - ABTS^•+^ (145.93 ± 6.08) - O_2_^•−^ (316.18 ± 12.16) - FRAP (112.3 ± 7.25)	in vitro: ABTS^•+^-, O_2_^•−^-scavenging power, FRAP test	[108]
(stems; collected in Qinghai, China)	cytotoxicity	isolated compounds: myricarin A and B	myricarin A and B: no cytotoxicity (IC_50_ > 10 μg/mL for all cell lines)	in vitro: MTT method; human cancer cell lines: Bel-7402; HCT-8, BGC823, A549 and MCF-7	[64]
(n.i., collected in Qinghai, China)	UVB-protective	pure compounds: kaempferol, rhamnazin, rhamnocitrin, quercetin-3-*O-*β-D-glucuronic acid, ferulic acid, caffeic acid, gallic acid	↑cell viability after 6 h in dose 3 μmol/L; identified most active compounds’ impact on cell viability, inhibit early cell apoptosis or delay cell apoptosis - ↑ SOD activity: kaempferol, gallic acid - ↓ MDA: kaempferol, gallic acid - ↓ IL-6: kaempferol, rhamnocitrin, quercetin-3-*O*-β-D-glucuronic acid, ferulic acid - ↓ TNF-α: ferulic acid, caffeic acid, gallic acid - ↓ Caspase-3: kaempferol, quercetin-3-*O*-β-D-glucuronic acid	in vitro: CCK-8 assay, flow cytometry HaCaT cells after UVB irradiation	[92]
			proposed core targets related to skin inflammation and mechanisms of action	in silico: molecular docking and network pharmacology for selected compounds	
*M. squamosa*					
(overground parts; collected in Qinghai, China)	antioxidant	isolated compounds: myricarin A, C	EC_50_ [μg/mL]: - myricarin A (40.9), myricarin C (42.22) - positive control: rutin (5.17)	in vitro: DPPH test	[67]
*M. wardii*					
(leafy twigs, branches; collected in China)	antioxidant	isolated compounds: methyl 3,4-dihydroxy-5-methoxybenzoate, protocatechualdehyde, protocatechuic acid, myricarin A	IC_50_ [mM]: - methyl 3,4-dihydroxy-5-methoxybenzoate (0.26 ± 0.02), myricarin A (0.31 ± 0.1), protocatechualdehyde (2.43 ± 0.26), protocatechuic acid (0.42 ± 0.01) - positive control: Trolox (0.28 ± 0.01)	in vitro: ABTS test	[45]
	anti-inflammatory	95% EtOH extract; PE, EA, BuOH, H_2_O fractions	dose 100 mg/kg: - ↓IL-6, ↓ MPO activity in serum or lung tissue; alleviated the damages of lung tissue - EtOH, EA, H_2_O: ↓ MPO, ↓ IL-6 (serum, lung) - PE: ↓ MPO, ↓ IL-6 (serum) but no significant effect in lung - BuOH: ↓ MPO, ↓ IL-6 (lung), no effect on IL-6 in serum	in vivo: MPO activity in lung tissue IL-6 level in serum and lung tissue; LPS-infected male BALB/c mice	[45]
	anti-complementary	95% EtOH extract; PE, EA, BuOH, H_2_O fractions; isolated compounds: methyl 3.4-dihydroxy-5-methoxybenzoate, protocatechualdehyde, protocatechuic acid, myricarin A	half-inhibit hemolysis concentration (CH_50_ [mM]): - extract and fractions: (5.86 ± 0.12 to 21.57 ± 0.29 μg/mL) - methyl 3.4-dihydroxy-5-methoxybenzoate (0.92 ± 0.38); protocatechualdehyde (0.2 ± 0.03), protocatechuic acid (1.02 ± 0.16), myricarin A (0.34 ± 0.08) - positive control: heparin (20.21 ± 2.0 μg/mL)	in vitro: hemolysis test	[45]

↑ increase level, activation, up-regulation, stimulation; ↓ decrease level, inhibition, down-regulation, reduction; n.i. not indicated. Abbreviations: A549—human lung cancer cell line; AA—adjuvant arthritis; ABTS—2,2′-azinobis-(3-ethylbenzothiazoline-6-sulphonate); AChE—acetylcholinesterase; AIA—adjuvant-induced arthritis; ALT—alanine aminotransferase; AST—aspartate aminotransferase; Bel-7402—human liver cancer cell line; BGC823—human gastric cancer cell line; BHT—butylhydroxytoluene; BuOH—n-butanol; CCK-8—Cell Counting Kit-8; CH_50_—haemolytic complement; CIA—collagen-induced arthritis; CS-Au-MGP—*Myricaria germanica* polysaccharide chitosan–gold complex; DCM—dichloromethane; DPPH—2,2-diphenyl-1-picrylhydrazyl; DTNB—5,5-dithio-bis-(2-nitrobenzoic acid), Ellman’s Reagent; EA—ethyl acetate; EC_50_—half maximal effective concentration; EGF—epidermal growth factor; ELISA—enzyme linked immunosorbent assay; EtOH—ethanol; FRAP—ferric ion reducing antioxidant power; HaCaT—normal keratinocyte cell line; HCT-8—human large intestine adenocarcinoma; HCT-15—colon cancer cell line; HDL—high-density lipoprotein cholesterol; HeLa—cervix cancer cell line; HepG2—liver carcinoma cell line; Huh-7—liver cancer cell line (hepatocyte-derived carcinoma cell line); H_2_O—water; IC_50_—half maximal inhibitory concentration; IFN-γ—interferon gamma; IgG1—immunoglobulin G1; IgG2—immunoglobulin G2; IL-1β—interleukin-1β; IL-2—interleukin 2; IL-4—interleukin 4; IL-6—interleukin-6; LDL—low-density lipoprotein cholesterol; iNOS—inducible nitric oxide synthase; LD_50_—median lethal dose; LPS—lipopolysaccharide; MAPK—mitogen-activated protein kinases; MCF-7—human breast cancer cell line; MDA—malondialdehyde; MeOH—methanol; MIC—minimal inhibitory concentration; MIN6—mouse insulinoma cell line; MPO—myeloperoxidase; MTT—(3-(4,5-dimethylthiazol-2-yl)-2,5-diphenyltetrazolium bromide) assay; NF-κBnuclear factor kappa B; NO—nitric oxide; O_2_^•−^—superoxide anion; PARP—poly (ADP-ribose) polymerase; p38—38 kDa polypeptide; p65—65 kDa polypeptide; PC-3—prostate cancer cell line; PCR—polymerase chain reaction; PE—petroleum ether; PLGA—poly(lactic-coglycolic-acid); PREP—prolyl endopeptidase; RAW 264.7—a macrophage-like, Abelson leukemia virus-transformed cell line derived from BALB/c mice; ROS—reactive oxygen species; SOD—superoxide dismutase; SRB—sulphorhodamine B; RA—rheumatoid arthritis; TC—total cholesterol; TG—triglyceride; THP-1—leukemia cell line; TNF-α—tumor necrosis factor α; UVB—ultraviolet B.

## Data Availability

Not applicable.

## Supplementary Materials

The following supporting information can be downloaded at https://www.mdpi.com/article/10.3390/life16050832/s1, Table S1: Summary of quantitative studies on the chemistry of *Myricaria* species; Table S2: Chemical constituents and relative contents of the essential oil from *M. germanica*.

## Abbreviations

The following abbreviations are used in this manuscript:

5-LOX	lipoxygenase-5
A549	human lung cancer cell line
AA	adjuvant arthritis
AAS	Atomic Absorption Spectrometry
ABTS	2,2′-azinobis-(3-ethylbenzothiazoline-6-sulphonate)
AChE	acetylcholinesterase
AIA	adjuvant-induced arthritis
ALT	alanine aminotransferase
APG	The Angiosperm Phylogeny Group
AST	aspartate aminotransferase
Bel-7402	human liver cancer cell line
BGC823	human gastric cancer cell line
BHT	butylhydroxytoluene
BuOH	*n*-butanol
CCK-8	Cell Counting Kit-8
CH_50_	haemolytic complement
CIA	collagen-induced arthritis
CS-Au-MGP	*Myricaria germanica* polysaccharide chitosan–gold complex
CS–Au–MGP NPs	*Myricaria germanica* polysaccharide chitosan–gold nanoparticle complex
COX-2	cyclooxygenase-2 enzyme
CZE	Capillary Zone Electrophoresis
DCM	dichloromethane
DNA	deoxyribonucleic acid
DPPH	2,2-diphenyl-1-picrylhydrazyl
DTNB	5,5-dithio-bis-(2-nitrobenzoic acid), Ellman’s Reagent
EA	ethyl acetate
EC_50_	half maximal effective concentration
EGF	epidermal growth factor
EGFR	epidermal growth factor receptor
elemen. anal.	elementary analysis
ELISA	enzyme-linked immunosorbent assay
ERK	extracellular signal-regulated kinase
ESI-MS	Electrospray Ionization Mass Spectrometry
EtOH	ethanol
FAAS	Flame Atomic Absorption Spectroscopy
FRAP	ferric ion reducing antioxidant power assay
FTMS	Fourier Transform Mass Spectrometry
GC	Gas Chromatography
GC-FID	Gas Chromatography with Flame Ionization Detector
GC-MS	Gas chromatography–mass spectrometry
GLC	Gas–Liquid Chromatography
HaCaT	normal keratinocyte cell line
HCT-8	human large intestine adenocarcinoma
HCT-15	colon cancer cell line
HDL	high-density lipoprotein cholesterol
HeLa	cervix cancer cell line
HepG2	liver carcinoma cell line
Huh-7	liver cancer cell line (hepatocyte-derived carcinoma cell line)
HPLC	High Performance Liquid Chromatography
HPLC-UV-DAD	High-Performance Liquid Chromatography with UV-Diode Array Detector
HREIMS	High-Resolution Electron Ionization Mass Spectrometry
HR-ESI-MS	High-Resolution Electrospray Ionization Mass Spectrometry
IC_50_	half maximal inhibitory concentration
IFN-γ	interferon gamma
H_2_O	water
IgG1	immunoglobulin G1
IgG2	immunoglobulin G2
IgG2a	immunoglobulin G2a
IL-1β	interleukin 1β
IL-2	interleukin
IL-4	interleukin 4
IL-6	interleukin 6
IL-17	interleukin 17
IR	Infrared spectroscopy
iNOS	inducible nitric oxide synthase;
JNK	c-Jun N-terminal kinase (MAPK family)
LC-MS	Liquid Chromatography-Mass Spectrometry
LC-MS/MS	Liquid Chromatography-Mass Spectrometry tandem Mass Spectrometry
LD_50_	median lethal dose
LDL	low-density lipoprotein cholesterol
LPS	lipopolysaccharide
LTB_4_	leukotriene B_4_
MAPK	mitogen-activated protein kinases
MDA	malondialdehyde
MeOH	methanol
MCF-7	human breast cancer cell line
MGP	*Myricaria germanica* polysaccharide
MIC	minimal inhibitory concentration
MIN6	mouse insulinoma cell line
MMP-9	matrix metalloproteinase-9
MPO	myeloperoxidase
MTT	3-(4,5-dimethylthiazol-2-yl)-2,5-diphenyltetrazolium bromide assay
NF-κB	nuclear factor kappa B
NMR	Nuclear Magnetic Resonance
NO	nitric oxide
O_2_^•−^	superoxide anion
p38	38 kDa polypeptide
p65	65 kDa polypeptide
PARP	poly (ADP-ribose) polymerase
PC-3	prostate cancer cell line
PCR	polymerase chain reaction
PE	petroleum ether
PLGA	poly(lactic-co-glycolic acid)
POWO	The Plants of the World Online database
PREP	prolyl endopeptidase
PREPi	prolyl endopeptidase inhibitor
PTGS2	prostaglandin-endoperoxidase synthase 2
RA	rheumatoid arthritis
RAW 264.7	a macrophage-like, Abelson leukemia virus-transformed cell line derived from BALB/c mice
ROS	reactive oxygen species
SOD	superoxide dismutase
SRB	sulphorhodamine B
TBARS	thiobarbituric acid reactive species assay
TC	total cholesterol
TG	triglyceride
THP-1	leukemia cell line
TLC	Thin-Layer Chromatography
TNF-α	tumor necrosis factor α
UPLC-Q-TOF MS/MS	Ultra-Performance Liquid Chromatography combined with Quadrupole Time-of-Flight Tandem Mass Spectrometry
UV	Ultraviolet Spectroscopy
UVB	ultraviolet B

## References

[1] POWO, The Plants of the World Online Database. Facilitated by the Royal Botanic Gardens, Kew. https://powo.science.kew.org/.

[2] Sitzia T., Kudrnovsky H., Müller N., Michielon B. (2021). Biological Flora of Central Europe: *Myricaria germanica* (L.) Desv.. Perspect. Plant Ecol. Evol. Syst..

[3] Dörken V.M., Parsons R.F., Marshall A.T. (2017). Studies on the Foliage of *Myricaria germanica* (Tamaricaceae) and Their Evolutionary and Ecological Implications. Trees.

[4] Costa L.M.S., Goetze M., Rodrigues A.V., dos Santos Seger G.D., Bered F. (2020). Global Rheophytes Data Set: Angiosperms and Gymnosperms. Ecology.

[5] Chase M.W., Christenhusz M.J.M., Fay M.F., Byng J.W., Judd W.S., Soltis D.E., Mabberley D.J., Sennikov A.N., Soltis P.S., The Angiosperm Phylogeny Group (2016). An Update of the Angiosperm Phylogeny Group Classification for the Orders and Families of Flowering Plants: APG IV. Bot. J. Linn. Soc..

[6] The World Flora Online. http://www.worldfloraonline.org.

[7] Gaskin J.F. (2003). Tamaricaceae. The Families and Genera of Vascular Plants; Flowering Plants, Dicotyledons.

[8] Zhang D., Zhang Y., Gaskin J.F., Chen Z. (2006). Systematic Position of *Myrtama* Ovcz. & Kinz. Based on Morphological and nrDNA ITS Sequence Evidence. Chin. Sci. Bull..

[9] Wang Y., Liu Y., Liu S., Huang H. (2009). Molecular Phylogeny of *Myricaria* (Tamaricaceae): Implications for Taxonomy and Conservation in China. Bot. Stud..

[10] Qaiser M., Ali S.I. (1978). Tamaricaria, a New Genus of Tamaricaceae. Blumea.

[11] Zhang D.Y., Chen Z.D., Sun H.Y., Yin L.K., Pan B.R. (2000). Systematic studies on some questions of Tamaricaceae based on ITS sequence. Acta Bot. Boreali-Occident. Sin..

[12] Zeng Y., Bao M., Ma J., Chen H., Zhang G., Zhang H. (2011). Immunoregulatory Mechanisms of the Total Flavonoids of Tibetan Herb *Myricaria germanica* auct. non Linn. Desv. in Adjuvant Arthritic Rat. Chin. Pharmacol. Bull..

[13] Liu L., Yuan W., Liu Z., Zuo W., Zhang R., Wang Z., Li J. (2023). Elucidating the Material Basis and Potential Mechanisms of *Myricaria germanica* Acting on Rheumatoid Arthritis by UPLC-Q-TOF-MS /MS and Network Pharmacology. Phytomed. Plus.

[14] Yang Q., Gaskin J., Wu Z.Y., Raven P.H., Hong D.Y. (2007). Tamaricaceae. Flora of China.

[15] Don G., Miller P. (1832). A General System of Gardening and Botany. Founded upon Miller’s Gardener’s Dictionary, and Arranged According to the Natural System.

[16] Grierson A.J.C., Long D.G. (1991). Flora of Bhutan; Illustrations by Mary Bates and Glenn Rodrigues.

[17] Grierson A.J.C., Long D.G. (1982). Notes Relating to the Flora of Bhutan: V. New and Noteworthy Plants Collected in Bhutan in 1979. Notes R. Bot. Gard. Edinb..

[18] Astashenkov A., Lyakh E. (2020). Ontogenesis and Architectural Analysis of *Myricaria bracteata* Royal in East Kazakhstan. BIO Web Conf..

[19] Wang Y., Liu Y.F., Liu S.B., Huang H.W. (2006). Geographic Distribution and Current Status and Conservation Strategy of the Genus Myricaria in China. J. Wuhan Bot. Res..

[20] Karpova E.A., Krasnikov A.A., Lyakh E.M., Chernonosov A.A. (2022). Leaf Surface Secretion of Flavonoids in *Myricaria bracteata* and *M. longifolia* (Tamaricaceae). Taiwania.

[21] Lyakh E.M., Tsybuloya N.V. (2009). To the study of antibacterial and antifungal activity of volatile emissions of *Myricaria bracteata* (Tamaricaceae). Rastitielnye Resur..

[22] Qaiser M. (1982). Flora of Pakistan.

[23] Efloraofindia (2007 Onwards) Database of Plants of the Indian Subcontinent. https://efloraofindia.com/.

[24] Liu Y., Wang Y., Huang H. (2006). High Interpopulation Genetic Differentiation and Unidirectional Linear Migration Patterns in *Myricaria laxiflora* (Tamaricaceae), an Endemic Riparian Plant in the Three Gorges Valley of the Yangtze River. Am. J. Bot..

[25] Huang G.Y., Zhang H.L., Qiu L.W., Li W., Li H., Lei H., Guo Y., Hu H., Zhang D.Y., Song Z.J. (2023). Population and Community Characteristics of Remnant Populations of Endangered *Myricaria laxiflora*. Plant Sci. J..

[26] University of Greifswald, Institute of Botany and Landscape Ecology, Institute of Geography and Geology, Computer Centre (2010). FloraGREIF—Virtual Flora of Mongolia.

[27] Hu H., Wang Q., Hao G., Zhou R., Luo D., Cao K., Yan Z., Wang X. (2023). Insights into the Phylogenetic Relationships and Species Boundaries of the *Myricaria squamosa* Complex (Tamaricaceae) Based on the Complete Chloroplast Genome. PeerJ.

[28] Fang J., Wang Z., Tang Z. (2011). Atlas of Woody Plants in China.

[29] Ni J., Herzschuh U. (2011). Simulating Biome Distribution on the Tibetan Plateau Using a Modified Global Vegetation Model. Arct. Antarct. Alp. Res..

[30] Shrestha M.R., Rokaya M.B., Ghimire S.K. (2005). Vegetation Pattern of Trans-Himalayan Zone in the North-West Nepal. Nepal J. Plant Sci..

[31] Sun K., Werth S. (2024). Journey to the West: Migration Patterns of the Riparian Montane Genus *Myricaria*. Flora.

[32] Liu Y., Wang Y., Huang H. (2009). Species-level Phylogeographical History of *Myricaria* Plants in the Mountain Ranges of Western China and the Origin of *M. laxiflora* in the Three Gorges Mountain Region. Mol. Ecol..

[33] Lyakh E.M. (2013). Overview of the Study of the Genus *Myricaria* Desv. in Siberia. Eurasian J. For. Res..

[34] Lyakh E.M., Astashenkov A.Y. (2019). Coenopopulations of *Myricaria bracteata* Royal at the Territory of the Republic of Kazakhstan and Their Ontogenetic Structure. BIO Web Conf..

[35] Lyakh E.M., Astashenkov A.Y. (2021). Features of the Population Organization of *Myricaria bracteata* in the Riverbed of the Ursul River (Gorny Altai). BIO Web Conf..

[36] Sitzia T., Michielon B., Iacopino S., Kotze J. (2016). Population dynamics of the endangered shrub *Myricaria germanica* in a regulated Alpine river is influenced by active channel width and distance to check dams. Ecol. Eng..

[37] Danube Pollution Reduction Programme Evaluation of Wetlands and Floodplain Areas in the Danube River Basin Final Report 1999. Programme Coordination Unit UNDP/GEF Assistance prepared by WWF Dan-ube-Carpathian-Programme and WWF-Auen-Institut (Germany). https://www.icpdr.org/sites/default/files/EVALUATIONWETLANDSFLOODPLAINAREAS.pdf.

[38] The Council of the European Communities (1992). Council Directive 92/43/EEC of 21 May 1992 on the Conservation of Natural Habitats and of Wild Fauna and Flora 1992. Off. J. Eur. Union.

[39] Lyakh E. (2019). Biological Aspects of the Genus *Myricaria* Desv. (Tamaricaceae), Especially of the Siberian Species. BIO Web Conf..

[40] Michielon B. (2019). Conservation of the Endangered *Myricaria germanica* (L.) Desv.: A Keystone Species of Riverine Habitats.

[41] Hegnauer R. (1973). Tamaricaceae. Chemotaxonomie der Pflanzen; Lehrbücher und Monographien aus dem Gebiete der Exakten Wissenschaften.

[42] Ntie-Kang F., Njume L.E., Malange Y.I., Günther S., Sippl W., Yong J.N. (2016). The Chemistry and Biological Activities of Natural Products from Northern African Plant Families: From Taccaceae to Zygophyllaceae. Nat. Prod. Bioprospect..

[43] Swilam N. (2014). Chemistry and Biology of Phenolics Isolated from *Myricaria germanica* (L.) Desv. (Tamaricaceae).

[44] Mubashir S. (2011). Phytochemical Screening of Major Constituents of Various Folklore Medicinal Plants of Kashmir Valley.

[45] Zhang M., Deji, Chen D., Lu Y. (2025). Chemical Recognition and Spectrum-Effect Relationship of UPLC-MS Chromatograms with Anti-Complementary and Antioxidant Activities of Myricariae Ramulus. Phytochem. Anal..

[46] Troshchenko A.T., Povolotskaya N.N. (1967). 12-Hentriacontanol from *Myricaria alopecuroides*. Chem. Nat. Compd..

[47] Bandyukova V.A., Dzhumyrko S.F. (1970). Flavonol Glycosides of Some Plants of the Teberda Reserve. I. Chem. Nat. Compd..

[48] Chumbalov T.K., Bikbulatova T.N., Il’yasova M.I. (1974). Polyphenols of *Myricaria alopecuroides*. I. Chem. Nat. Compd..

[49] Chumbalov T.K., Bikbulatova T.N., Il’yasova M.I., Mukhamedieva R.M. (1975). Polyphenols of *Myricaria alopecuroides* II. Flavonoid Aglycones. Chem. Nat. Compd..

[50] Chumbalov T.K., Bukbulatova T.N., Il’yasova M.I. (1976). Polyphenols of *Myricaria alopecuroides*. III. Hydrolyzable Tanning Substances. Chem. Nat. Compd..

[51] Chumbalov T.K., Bikbulatova T.N., Il’yasova M.I. (1979). Polyphenols of *Myricaria alopecuroides*. IV. Hydrolysable Tanning Substances. Chem. Nat. Compd..

[52] Semenova L.S. (1993). Flavonoid composition of shoots of *Myricaria longifolia* (Willd.) Ehrenb. Rastit. Resur. Plant Resour..

[53] Zhao D.B., Liu X.H., Cui S.Y., Wang T., Wang H.Q. (2005). Separation and Determination of Six Active Components in Two *Myricaria* Plants by Capillary Chromatography. Chromatographia.

[54] Nawwar M.A., Swilam N.F., Hashim A.N., Al-Abd A., Abdel-Naim A.B., Lindequist U. (2013). Cytotoxic Isoferulic Acidamide from *Myricaria germanica* (Tamaricaceae). Plant Signal. Behav..

[55] Chernonosov A.A., Karpova E.A., Lyakh E.M. (2017). Identification of Phenolic Compounds in *Myricaria bracteata* Leaves by High-Performance Liquid Chromatography with a Diode Array Detector and Liquid Chromatography with Tandem Mass Spectrometry. Rev. Bras. Farmacogn..

[56] Karpova E., Davlatov S.K., Chernonosov A. (2021). Phenolic Compounds in Taxonomy of *Myricaria longifolia* and *Myricaria bracteata* (Tamaricaceae). BIO Web Conf..

[57] Zhou R., Wang T., Du X.Z. (2006). Studies on chemical constituents in herb of *Myricaria bracteata*. China J. Chin. Mater. Med..

[58] Li S., Chen R.Y., Yu D.Q. (2007). Study on Chemical Constituents of *Myricaria paniculata* I. China J. Chin. Mater. Med..

[59] Li Z., Xue P., Xie P., Li X., Xie M. (2010). Chemical constituents from *Myricaria alopecuroides*. China J. Chin. Mater. Med..

[60] La X.Q., Zeng Y., Xu M., Zhang Y.J. (2011). Flavonoids from the Twigs of the Tibetian Medicine *Myricaria germanica*. Nat. Prod. Res. Dev..

[61] Liu J.-B., Ding Y.-S., Zhang Y., Chen J.-B., Cui B.-S., Bai J.-Y., Lin M.-B., Hou Q., Zhang P.-C., Li S. (2015). Anti-Inflammatory Hydrolyzable Tannins from *Myricaria bracteata*. J. Nat. Prod..

[62] Liu J.-B., Zhang Y., Cui B.S., Shuai L. (2013). Chemical constituents from twigs of *Myricaria bracteata*. Chin. Tradit. Herb. Drugs.

[63] Tian W., Zheng Y., Xue Y., Zou K., Liu S. (2012). Identification of Gallicin from *Myricaria laxiflora* and Its Antibiotic & Antioxidant Activity. Proceedings of the 2012 International Symposium on Information Technologies in Medicine and Education, Hokodate, Japan.

[64] Li S., Dai S.-J., Chen R.-Y., Yu D.-Q. (2005). Triterpenoids from the Stems of *Myricaria paniculata*. J. Asian Nat. Prod. Res..

[65] Zhang K., Han M.-J., Wang L., Liu S., Zhao X.-F., Shen W.-Y., Mao H.-Q., Qin D., Dong J.-Y. (2016). A New Oleanane Type Triterpenoid and Other Constituents from the Waterlogging Tolerant Plant *Myricaria laxiflora*. Biochem. Syst. Ecol..

[66] Zhang Y., Yuan Y., Cu B., Li S. (2011). Study on chemical constituents from ethyl acetate extract of *Myricaria bracteata*. China J. Chin. Mater. Med..

[67] Xu H., Yuan Z.-Z., Ma X., Wang C., Suo Y.-R., Wang H.-L., Wang X.-Y., Bai B. (2018). Triterpenoids with Antioxidant Activities from *Myricaria squamosa*. J. Asian Nat. Prod. Res..

[68] Jetter R. (2000). Long-Chain Alkanediols from *Myricaria germanica* Leaf Cuticular Waxes. Phytochemistry.

[69] Gendaram O. (2016). Anti-Oxidative, Acetylcholinesterase and Pancreatic Lipase Inhibitory Activities of Compounds from *Dasiphora fruticosa*, *Myricaria alopecuroides* and *Sedum hybridum*. Mong. J. Chem..

[70] Huan M., Umbetova A.K., Jenis J., Sagdullaev S.S., Gemedzhieva N.G. Chemical Research of the Genus Myricaria (I), Book of Abstract. Proceedings of the International Scientific Conference on Actual Problems of the Chemistry of Natural Compounds.

[71] Shults E.E., Raldugin V.A., Volcho K.P., Salakhutdinov N.F., Tolstikov G.A. (2007). Plant Metabolites of the Siberian Flora. Chemical Transformations and the Scope of Practical Application. Russ. Chem. Rev..

[72] Harborne J.B. (1975). Flavonoid Bisulphates and Their Co-Occurences with Ellegic Acid in the Bixaceae, Frankeniaceae and Related Families. Phytochemistry.

[73] Ullah K., Azam T., Sammad A., Ahmed T., Wadood A., Ali M.F., Mushraf G.M., Shaheen Siddiqui B., Li Y. (2025). Novel Prolyl Endopeptidase Inhibitor from *Myricaria germanica* Alleviates Steatohepatitis. RSC Adv..

[74] Zeng Y., La X.Q., Xu M., Zhang Y.J. (2012). Phenolic Constituents from Twigs of Tibetan Medicine *Myricaria germanica*. Nat. Prod. Res. Dev..

[75] Wang Y., Qiu F., Zheng Q., Hong A., Wang T., Zhang J., Lin L., Ren Z., Qin T. (2024). Preparation, Characterization and Immune Response of Chitosan-gold Loaded *Myricaria germanica* Polysaccharide. Int. J. Biol. Macromol..

[76] Mraz B.A. (2010). Beiträge zur Phytochemischen Analyse der Mongolischen Heilpflanze Myricaria longifolia Ehrenb..

[77] Karpova E.A., Buglova L.V., Lyakh E.M., Shaldaeva T.M. (2025). Ecological Aspects of the Composition and Tissue Distribution of Phenolic Compounds in *Myricaria bracteata* and *Myricaria longifolia* Twigs. Contemp. Probl. Ecol..

[78] Kubasa B. (2011). Analyse und Isolierung Sulfatierter und Glykosylierter Flavonoide aus der Mongolischen Arzneipflanze Myricaria longifolia Ehrenb..

[79] Donath O., Glasl S., Kletter C., Narantuya S. (2006). Phytochemical Investigation of *Myricaria longifolia* Ehrenb.—A Plant Used in Traditional Mongolian Medicine. Sci. Pharm..

[80] Obmann A., Mraz B., Kubasa B., Zehl M., Kletter C., Glasl S. (2010). Phytochemical Profiling of the Mongolian Medicinal Plant *Myricaria longifolia* Ehrenb. Planta Med..

[81] Pan J.J., Zhang X.L., Yang N., Fan Y. (2018). Study on the chemical constituents of *Myricaria squamosa*. J. Pharm. Res..

[82] Iwashina T. (2013). Flavonoid Properties of Five Families Newly Ncorporated into the Order Caryophyllales (Review). Bull. Natl. Mus. Nat. Sci. Ser. B.

[83] Harborne J.B. (1975). Flavonoid Sulphates: A New Class of Sulphur Compounds in Higher Plants. Phytochemistry.

[84] Mohammed H.A., Ragab E.A., Dutta A., Othman A., Shaheen U., Jaremko M., Badshah S.L., Dhahri M., Al-Younis I., Al-Rimawi F. (2025). Sulfated Flavonoids: An Updated and Comprehensive Review of Their Chemistry and Bioactivities. Phytochem. Rev..

[85] Teles Y., Souza M., Souza M. (2018). Sulphated Flavonoids: Biosynthesis, Structures, and Biological Activities. Molecules.

[86] Chung K.-T., Wong T.Y., Wei C.-I., Huang Y.-W., Lin Y. (1998). Tannins and Human Health: A Review. Crit. Rev. Food Sci. Nutr..

[87] Mavlyanov S.M., Islambekov S.Y., Ismailov A.I., Dalimov D.N., Abdulladzhanova N.G. (2001). Vegetable Tanning Agents. Chem. Nat. Compd..

[88] Ahmad M., Ahmad W., Khan S., Zeeshan M., Obaidullah, Nisar M., Shaheen F., Ahmad M. (2008). New Antibacterial Pentacyclic Triterpenes from *Myricaria elegans* Royle. (Tamariscineae). J. Enzyme Inhib. Med. Chem..

[89] Khan S., Nisar M., Khan R., Ahmad W., Nasir F. (2010). Evaluation of Chemical Constituents and Antinociceptive Properties of *Myricaria elegans* Royle. Chem. Biodivers..

[90] Umbetova A.K., Choudhary M.I., Sultanova N.A., Burasheva G.S., Abilov Z.A. (2006). Triterpenoids from the Genus *Tamarix*. Chem. Nat. Compd..

[91] Zeng Y., Chen R., Ma X., Lei Y. (2014). Analysis of Chemical Components of Volatile Oil from *Myricaria germanica* by GC-MS. Nat. Prod. Res. Dev..

[92] Liu L., Ma J., Chen W., Zhang J., Zuo W., Wang M., Li J. (2025). UVB-Induced HaCat Cell Damage and *Myricaria paniculata*’s Molecular Effects. Sci. Rep..

[93] Sun Y.-L., Hong S.-K. (2011). Effects of Citric Acid as an Important Component of the Responses to Saline and Alkaline Stress in the Halophyte *Leymus chinensis* (Trin.). Plant Growth Regul..

[94] Chen C., Fan G., Lin Y., Zheng Z., Zhang X., Zhang Y., Qin T., Ren Z. (2026). Selenium *Myricaria germanica* Polysaccharides-based Nanoparticles to Improve Immune Activity. Chem. Biodivers..

[95] Jiao Y., Yang Y., Zhou L., Chen D., Lu Y. (2022). Two Natural Flavonoid Substituted Polysaccharides from *Tamarix chinensis*: Structural Characterization and Anticomplement Activities. Molecules.

[96] Jiao Y., Zhou L., Huo J., Li H., Zhu H., Chen D., Lu Y. (2024). Flavonoid Substituted Polysaccharides from *Tamarix chinensis* Lour. Alleviate H1N1-Induced Acute Lung Injury via Inhibiting Complement System. J. Ethnopharmacol..

[97] Jiao Y., Zhou L., Li H., Zhu H., Chen D., Lu Y. (2024). A Novel Flavonol-Polysaccharide from *Tamarix chinensis* Alleviates Influenza A Virus-Induced Acute Lung Injury. Evidences for Its Mechanism of Action. Phytomedicine.

[98] Shyshkin B.K., Bobrov E.G. (1949). Flora CCCP.

[99] World Health Organisation (WHO) (2007). Report of Marked Research on Mongolian Traditional Medicinal Drugs.

[100] Khookhor O., Sato Y. (2009). Mongolian Plant Extracts with Potential Glucose Absorption Inhibiting Effects in Rats. J. Trad. Med..

[101] Popov P.L. (2008). Plant species, using against virous infections of man and animals: Regularities of the distribution in the phylogenetic classification system. J. Stress Physiol. Biochem..

[102] Rinpoche R. (1976). Tibetan Medicine.

[103] World Health Organization (2013). Medicinal Plants in Mongolia.

[104] Kletter C., Glasl S., Thalhammer T., Narantuya S. (2008). Traditional Mongolian Medicine—A Potential for Drug Discovery. Sci. Pharm..

[105] Sharma L., Samant S.S., Kumar A., Negi D., Devi K., Lal M., Tewari L.M. (2017). Diversity, Distribution Pattern and Indigenous Uses of Medicinal Plants of Cold Desert Biosphere Reserve in Trans Himalaya. J. Ethnobiol. Tradit. Med..

[106] Adhikari B.S., Babu M.M., Saklani P.L., Rawat G.S. (2007). Distribution, Use Pattern and Prospects for Conservation of Medicinal Shrubs in Uttaranchal State, India. J. Mt. Sci..

[107] Quattrocchi U. (2012). CRC World Dictionary of Medicinal and Poisonous Plants.

[108] Bao Y.-F., Li J.-Y., Zheng L.-F., Li H.-Y. (2015). Antioxidant Activities of Cold-Nature Tibetan Herbs Are Signifcantly Greater than Hot-Nature Ones and Are Associated with Their Levels of Total Phenolic Components. Chin. J. Nat. Med..

[109] Wangchuk P., Keller P.A., Pyne S.G., Taweechotipatr M., Tonsomboon A., Rattanajak R., Kamchonwongpaisan S. (2011). Evaluation of an Ethnopharmacologically Selected Bhutanese Medicinal Plants for Their Major Classes of Phytochemicals and Biological Activities. J. Ethnopharmacol..

[110] Lama D. (1995). The Quintessence Tantras of Tibetan Medicine.

[111] Ding X., Guo C., Zhang X., Li J., Jiao Y., Feng H., Wang Y. (2022). Wild Plants Used by Tibetans in Burang Town, Characterized by Alpine Desert Meadow, in Southwestern Tibet, China. Agronomy.

[112] Lama Y.C., Ghimire S.K., Aumeeruddy-Thomas Y. (2001). Medicinal Plants of Dolpo: Amchis’ Knowledge and Conservation.

[113] Ministry of Health of Bhutan (2009). Monograph on Medicinal Plants of Bhutan.

[114] Bhattarai S., Chaudhary R.P., Quave C.L., Taylor R.S. (2010). The Use of Medicinal Plants in the Trans-Himalayan Arid Zone of Mustang District, Nepal. J. Ethnobiol. Ethnomed..

[115] Gewali M.B. (2008). Aspects of Traditional Medicine in Nepal.

[116] Dorji K., Tobgay S., Yangdon N. (2017). The Ethno-Botanical Studies of Medicinal and Aromatic Plants in Sakteng Wildlife Sanctuary, Trashigang, Bhutan. Int. J. Curr. Res. Biosci. Plant Biol..

[117] Bhattarai S., Chaudhary R.P., Taylor R.S. (2006). Ethnomedicinal Plants Used by the People of Manang District, Central Nepal. J. Ethnobiol. Ethnomed..

[118] Bano A., Ahmad M., Zafar M., Sultana S., Rashid S., Khan M.A. (2014). Ethnomedicinal Knowledge of the Most Commonly Used Plants from Deosai Plateau, Western Himalayas, Gilgit Baltistan, Pakistan. J. Ethnopharmacol..

[119] Witt C.M., Berling N.E.J., Rinpoche N.T., Cuomo M., Willich S.N. (2009). Evaluation of Medicinal Plants as Part of Tibetan Medicine Prospective Observational Study in Sikkim and Nepal. J. Altern. Complement. Med..

[120] Khan B., Abdukadir A., Qureshi R., Mustafa G. (2011). Medicinal Uses of Plants by the Inhabitants of Khunjerab National Park, Gilgit, Pakistan. Pak. J. Bot..

[121] Chaurasia O.P., Khatoon N., Singh S.B. (2008). Fields Guide Floral Diversity of Ladakh.

[122] Tsarong T.J. (1994). Tibetan Medicinal Plants.

[123] Soelberg J., Jäger A.K. (2016). Comparative Ethnobotany of the Wakhi Agropastoralist and the Kyrgyz Nomads of Afghanistan. J. Ethnobiol. Ethnomed..

[124] Liu H., Wagutu G.K., Chen Y., Li X., Fan X. (2024). The Complete Chloroplast Genome Sequence of *Myricaria wardii* Marquand 1929 (Tamaricaceae): A Shrub Species Endemic to the Tibet Plateau. Mitochondrial DNA Part B.

[125] Batool Z., Singh K., Gairola S. (2023). Medicinal Plants Traditionally Used in the Health Care Practices by the Indigenous Communities of the Trans-Himalayan Region of Ladakh, India. J. Ethnopharmacol..

[126] Rinchen T., Pant S. (2014). Ethnopharmacological Uses of Plants among Inhabitants Surrounding Suru and Zanskar Valleys of Cold Desert, Ladakh. Int. J. Pharma Bio Sci..

[127] Buth G.M., Navchoo I.A. (1988). Ethnobotany of Ladakh (India) Plants Used in Health Care. J. Ethnobiol..

[128] Kumar G.P., Gupta S., Murugan M.P., Bala Singh S. (2009). Ethnobotanical Studies of Nubra Valley—A Cold Arid Zone of Himalaya. Ethnobot. Leafl..

[129] Singh K.N. (2012). Traditional Knowledge on Ethnobotanical Uses of Plant Biodiversity: A Detailed Study from the Indian Western Himalaya. Biodivers. Res. Conserv..

[130] Singh K.N., Lal B. (2008). Ethnomedicines Used against Four Common Ailments by the Tribal Communities of Lahaul-Spiti in Western Himalaya. J. Ethnopharmacol..

[131] Kiem J. (1992). Ein Tamariskenvorkommen Im Sarntal. Berichte Bayer. Bot. Ges..

[132] Kirbağ S., Zengin F., Kursat M. (2009). Antimicrobial Activities of Extracts of Some Plants. Pak. J. Bot..

[133] Dogan Y., Nedelcheva A.M., Obratov-Petković D., Padure I.M. (2008). Plants Used in Traditional Handicrafts in Several Balkan Countries. IJTK.

[134] Zhang L., Cui Z., Mu Y., Wei K., Li Z., Zhu H., Yang D., Wang Y., Long P., Zhang C. (2015). Ethnopharmacological Investigation and Rapid Authentication of Mongolian Patent Medicines Digeda. Chin. Herb. Med..

[135] Lei X.-J., Sun H.-M., Qu Y.-X., Xu Q.-Z., Zhou Y., Xiao H., Li L.-J., Yang Z.-B. (2025). Research Overview of Ethnic Medicines for the Treatment of Rheumatoid Arthritis. Front. Pharmacol..

[136] Chen H., Shoumura S., Emura S., Isono H. (2009). Tibetan Medicated-Bath Therapy May Improve Adjuvant Arthritis in Rat. Evid. Based Complement. Altern. Med..

[137] Peng S.-Y., Liu Y., Bao X., Wang L., Zhang F., Wang F., Wang W. (2011). Inhibition of 5-Lipoxygenase and Cyclooxygenase-2 Pathways by Pain-Relieving Plaster in Macrophages. Pharm. Biol..

[138] Wang Y.-Z., Guo C.-Y., Zhong H.-G., Zhang W.-N., Wang D.-L., Wang X., Dong F.-H. (2008). In Vivo Effects of Pain Relieving Plaster on Closed Soft Tissue Injury in Rabbit Ears. BMC Complement. Altern. Med..

[139] Chen C., Li S.Q., Bao T., Zhang L., Seluzicki C., Mao J.J. (2020). A Systematic Review of CheeZheng Pain Relieving Plaster for Musculoskeletal Pain: Implications for Oncology Research and Practice. Integr. Cancer Ther..

[140] Geiger P.L. (1845). Pharmacopoea Universalis.

[141] Kraemer H. (1902). A Text-Book of Botany and Pharmacognosy.

[142] Zhuang Y., Liu J., Ma P., Bai J., Ding Y., Yang H., Fan Y., Lin M., Li S., Hou Q. (2017). Tamarixinin A Alleviates Joint Destruction of Rheumatoid Arthritis by Blockade of MAPK and NF-κB Activation. Front. Pharmacol..

[143] Gonching E., Erdenebat S., Togtoo O., Bataa S., Gendaram O., Kim Y.S., Ryu S.Y. (2008). Antimicrobial Activity of Mongolian Medicinal Plants. Nat. Prod. Sci..

[144] Zandanov A.O., Sambueva Z.G., Ubeeva I.P., Nikolaev S.M. (2009). The perspective choleretic species of the plant raw materials of Siberia. Vestn. Buryatskogo Gos. Univ. Filos. Bull. Buryat State Univ. Philos..

[145] Azam T., Noreen F., Siddiqui B.S., Hafizur R.M., Begum S. (2022). Suppression of β-Cell Apoptosis from H2O2-Induced Oxidative Stress in MIN6 Cells Using Methyl Gallate. J. Chem. Soc. Pak..

[146] Liu L., Wang M., Zhang J., Chen W., Zuo W., Zeng Y., Li J. (2024). The Inhibitory Effect and Mechanism of Action of *Myricaria germanica* Essential Oils on Skin Inflammation Based on Network Pharmacology and Molecular Docking. Lett. Drug Des. Discov..

[147] Zeng Y., Chen Z., Zhong L., Li H. (2005). Study on the Effects That Caused by the Tradition Tibetan Herb *Myricaria germanica* (L.) Desv. on the Immune Function of Cells and the Toxicity Tests. J. Qinghai Norm. Univ..

[148] Vogl C., Bresgen N., Eckl P.M., Glasl S., Kletter C. (2009). In Vitro Evaluation of the Hepatotoxic Potential of Aqueous and Methanolic Extracts of *Myricaria longifolia*. Sci. Pharm..

[149] Bao M., Zeng Y., Mi Q., Chen Z. (2006). Bacteriostasis study of the different extracts from the Traditional Tibetan Herb *Myricaria germanica* (L.) Desv in vitro. LiShiZhen Med. Mater. Med. Res..

[150] Draczkowski P., Tomaszuk A., Halczuk T., Strzemski M., Matosiuk D., Jozwiak K. (2016). Determination of Affinity and Efficacy of Acetylcholinesterase Inhibitors Using Isothermal Titration Calorimetry. Biochim. Biophys. Acta (BBA)—Gen. Subj..

[151] Bhattacharyya A. (1991). Ethnobotanical Observations in the Ladakh Region of Northern Jammu and Kashmir State, India. Econ. Bot..

[152] Himalayan Forest Research Institute (HFRI) (2010). HFRI Initiatives and Achievements in the Cold Deserts of Himachal Pradesh & Jammu & Kashmir 2010.

[153] Abbas Z., Bussmann R.W., Khan S.M., Umair M., Alam J., Salma, Hussain M., Ullah Z. (2021). Ethnobotany of Karakorum, Pakistan. Ethnobiology of Mountain Communities in Asia.

[154] Huang N., Tang L., Zhu F., Wu C., Hartley W., Zhou J.J., Xue S.G. (2019). Salt Ions Accumulation and Distribution Characteristics of Pioneer Plant Species at a Bauxite Residue Disposal Area. China J. Cent. S. Univ..

