# Supplementary material for: Genus Myricaria, the Smaller Sister of Tamarisks—Ornamental Value, Phytochemistry, Biological Activities and Traditional Uses
Source: Life (Basel). 2026 May 19;16(5):832. doi: 10.3390/life16050832 (PMC13208939; doi:10.3390/life16050832)
Supplement: Supplementary file 1 [file life-16-00832-s001.zip › life-4263097-supplementary.pdf]

Supplementary Material

Genus *Myricaria*, the smaller sister of tamarisks – ornamental value, phytochemistry, biological activities and traditional uses.

Justyna Makowska-Wąs [1], Danuta Sobolewska [1], Karolina Grabowska [1], Dagmara Wróbel-Biedrawa [1], and Irma Podolak [1,*]

[1] Chair of Pharmacognosy, Jagiellonian University Medical College, Kraków

* Correspondence: irma.podolak@uj.edu.pl

Table S1. Summary of quantitative studies on the chemistry of *Myricaria* species.

**Identified compound**	**Plant species / plant part**	**Content**	**Method of determination**	**References**
Total phenolics	*M. bracteata*	15.14 ± 1.48 % (gallic acid equivalent)	spectrophotometry, Folin Ciocalteu reagent	[69]
		33.86 ± 5.97 mg/g dw (leaves)	HPLC-DAD	[56]
		29.30 ± 0.28 mg/g dw	HPLC-DAD	[20]
	M. longifolia	57.54 ± 7.13 mg/g dw (leaves)	HPLC-DAD	[56]
		93.34 ± 0.46 mg/g dw	HPLC-DAD	[20]
Total flavonoids	*M. bracteata*	2.09 ± 0.03 % (rutin equivalent); 0.61 ± 0.02 %, (quercetin equivalent)	spectrophotometry	[69]
		4.21 ± 0.77 mg/g dw (leaves)	HPLC-DAD	[56]
		4.12 % (aerial parts)	n.i.	[70]
	M. longifolia	13.44 ± 1.23 mg/g dw (leaves)	HPLC-DAD	[56]
Total tannins	*M. bracteata*	7.84%	n.i.	[70]
		5.33 ± 0.11 % (pyrogallol equivalent), (green branches)	spectrophotometry, hide powder and Folin Ciocalteu reagent in pH<1	[69]
Carbohydrates	*M. bracteata*	6.77 % (aerial parts)	n.i.	[70]

Organic acids `	*M. bracteata*	7.25 ± 1.71 mg/g dw (leaves) 2.45-17.21 mg/g dw (hydrolysed extract of leaves)	HPLC-DAD	[56]
		1.09–13.87 mg/g dw (twigs) 0.29–17.21 mg/g dw (hydrolysed extract)	HPLC-DAD	[76]
	M. longifolia	7.30 ± 2.44 mg/g dw (leaves) 2.61-25.07 mg/g dw (hydrolysed extract of leaves)	HPLC-DAD	[56]
		1.76–12.02 mg/g dw (twigs) 0.18–25.38 mg/g dw (hydrolysed extract)	HPLC-DAD	[76]
Total ash	*M. bracteata*	5.78 % (aerial parts)	n.i.	[70]
Sulphate ash	*M. bracteata*	10.85 % (aerial parts)	n.i.	[70]
Fat-soluble antioxidants	*M. bracteata*	1.67 ± 0.03 mg/100 g (aerial parts)	n.i.	[70]
Water-soluble antioxidants	*M. bracteata*	2.93 ± 0.04 mg/100 g (aerial parts)	n.i.	[70]
Flavonoids				
Kaempferol	*M. bracteata*	0.10-0.42 mg/g dw (hydrolysed extract of leaves)	HPLC-DAD, LC-MS	[56]
	M. longifolia	0.04-0.08 mg/g dw (hydrolysed extract of leaves)	HPLC-DAD, LC-MS	[56]
Quercetin	*M. bracteata*	0.08 ± 0.02 mg/g dw (leaves) 0.12-1.64 mg/g dw (hydrolysed extract)	HPLC-DAD, LC-MS	[56]
		15.8 ± 0.5 μg/g dw (leaves) 3.2 ± 0.3 μg/g dw (leaf surface)	HPLC-DAD, LC-MS/MS	[20]
		0.00–0.87 mg/g dw (twigs) 0.01–1.64 mg/g dw (hydrolysed extract)	HPLC-DAD	[76]
	M. longifolia	1.20 ± 0.29 mg/g dw (leaves)	HPLC-DAD, LC-MS	[56]

		1.44-2.70 mg/g dw (hydrolysed extract)		
		1534 ± 80 µg/g dw (leaves)<br>12.4 ± 0.1 µg/g dw (leaf surface)	HPLC-DAD, LC-MS/MS	[20]
		0.05–1.93 mg/g dw (twigs)<br>0.02–2.70 mg/g dw (hydrolysed extract)	HPLC-DAD	[76]
Isorhamnetin	*M. bracteata*	0.60-11.73 mg/g dw (hydrolysed extract)	HPLC-DAD, LC-MS	[56]
	M. longifolia	0.14-0.55 mg/g dw (hydrolysed extract)	HPLC-DAD, LC-MS	[56]
Rhamnazin	*M. bracteata*	0.00-1.10 mg/g dw (hydrolysed extract)	HPLC-DAD, LC-MS	[56]
	M. longifolia	0.35-1.50 mg/g dw (hydrolysed extract)	HPLC-DAD, LC-MS	[56]
Hyperoside	*M. bracteata*	1.79 ± 0.85 mg/g dw (leaves)	HPLC-DAD, LC-MS	[56]
		1237 ± 16 µg/g dw (leaves)<br>37 ± 0.3 µg/g dw (leaf surface)	HPLC-DAD, LC-MS/MS	[20]
		0.54–4.04 mg/g dw (twigs)<br>0.31–18.12 mg/g dw (hydrolysed extract)	HPLC-DAD	[76]
	M. longifolia	6.06 ± 1.35 mg/g dw (leaves)	HPLC-DAD, LC-MS	[56]
		5873 ± 52 µg/g dw (leaves)<br>17.6 ± 0.2 µg/g dw (leaf surface)	HPLC-DAD, LC-MS/MS	[20]
Astragalin	*M. bracteata*	0.10 ± 0.02 mg/g dw (leaves)	HPLC-DAD, LC-MS	[56]
		1451.3 ± 8.2 µg/g dw (leaves)<br>4.7 ± 0.4 µg/g dw (leaf surface)	HPLC-DAD, LC-MS/MS	[20]
		0.04–2.74 mg/g dw (twigs)	HPLC-DAD	[76]
	M. longifolia	1.50 ± 0.44 mg/g dw (leaves)	HPLC-DAD, LC-MS	[56]
		1309 ± 8 µg/g dw (leaves)	HPLC-DAD, LC-MS/MS	[20]

		16.0 ± 0.2 µg/g dw (leaf surface)		
		0.07–1.01 mg/g (twigs)		[76]
Isoquercitrin	*M. bracteata*	79.0 ± 4.2 µg/g dw (leaves)<br>1.4 ± 0.1 µg/g dw (leaf surface)	HPLC-DAD, LC-MS/MS	[20]
	M. longifolia	1346 ± 10 µg/g dw (leaves)<br>0.9 ± 0.0 µg/g dw (leaf surface)	HPLC-DAD, LC-MS/MS	[20]
Avicularin	*M. bracteata*	50.0 ± 6.1 µg/g dw (leaves)<br>4.0 ± 0.3 µg/g dw (leaf surface)	HPLC-DAD, LC-MS/MS	[20]
	M. longifolia	2163 ± 10 µg/g dw (leaves)<br>9.3 ± 0.1 µg/g dw (leaf surface)	HPLC-DAD, LC-MS/MS	[20]
Narcissin	*M. bracteata*	0.08 ± 0.05 mg/g dw (leaves)	HPLC-DAD, LC-MS	[56]
		79.0 ± 2.9 µg/g dw (leaves)	HPLC-DAD, LC-MS/MS	[20]
		0.00–1.01 mg/g dw (twigs)	HPLC-DAD	[76]
	M. longifolia	0.28 ± 0.08 mg/g dw (leaves)	HPLC-DAD, LC-MS	[56]
		526 ± 25 µg/g dw (leaves)	HPLC-DAD, LC-MS/MS	[20]
		0.00–1.33 mg/g dw (twigs)	HPLC-DAD	[76]
Luteolin	*M. longifolia*	335.4 ± 6.8 µg/g dw (leaves)	HPLC-DAD, LC-MS/MS	[20]
Naringenin	*M. bracteata*	165.3 ± 5.4 µg/g dw (leaves)	HPLC-DAD, LC-MS/MS	[20]
		0.00–0.17 mg/g dw (twigs)<br>0.01–0.90 mg/g dw (hydrolysed extract)	HPLC-DAD	[76]
	M. longifolia	532.4 ± 22.5 µg/g dw (leaves)<br>8.5 ± 0.1 µg/g dw (leaf surface)	HPLC-DAD, LC-MS/MS	[20]
		0.00–1.65 mg/g dw (twigs)<br>0.01–1.35 mg/g dw (hydrolysed extract)	HPLC-DAD	[76]

Phenolic acids				
Gallic acid	*M. bracteata*	7.04 ± 2.33 mg/g dw (leaves)<br>0.96-6.20 mg/g dw (hydrolysed extract)	HPLC-DAD, LC-MS	[56]
		2.27–20.40 mg/g dw (twigs)<br>0.23–7.75 mg/g dw (hydrolysed extract)	HPLC-DAD	[76]
	M. longifolia	22.70 ± 7.39 mg/g dw<br>1.37-11.94 mg/g dw (hydrolysed extract)	HPLC-DAD, LC-MS	[56]
		2.90–67.20 mg/g dw (twigs)<br>0.12–7.65 mg/g dw (hydrolysed extract)	HPLC-DAD	[76]
Ellagic acid	*M. bracteata*	5.72 ± 0.22 mg/g dw	HPLC-DAD, LC-MS	[55]
		0.72 ± 0.51 mg/g dw (leaves)<br>2.42-8.67 mg/g dw (hydrolysed extract)	HPLC-DAD, LC-MS	[56]
		1002 ± 7 μg/g dw (leaves)	HPLC-DAD, LC-MS/MS	[20]
		0.82–11.87 mg/g dw (twigs)<br>0.15–8.67 mg/g dw (hydrolysed extract)	HPLC-DAD	[76]
	M. longifolia	0.83 ± 0.22 mg/g dw (leaves)<br>0.67-11.81 mg/g dw (hydrolysed extract)	HPLC-DAD, LC-MS	[56]
		995 ± 7 μg/g dw (leaves)<br>11.15 ± 0.1 μg/g dw (leaf surface)	HPLC-DAD, LC-MS/MS	[20]
		0.22–13.50 mg/g dw (twigs)<br>0.26–11.81 mg/g dw (hydrolysed extract)	HPLC-DAD	[76]
Ferulic acid	*M. bracteata*	1.98 ± 0.26 mg/g dw (leaves)	HPLC-DAD, LC-MS	[56]
		486 ± 5 μg/g dw (leaves)<br>9.3 ± 0.5 (leaf surface)	HPLC-DAD, LC-MS/MS	[20]
		0.02–2.76 mg/g dw (twigs)	HPLC-DAD	[76]

		0.03–0.49 mg/g dw (hydrolysed extract)		
	M. longifolia	0.15 ± 0.05 mg/g dw (leaves)	HPLC-DAD, LC-MS	[56]
		221 ± 1 μg/g dw (leaves)<br>0.5 ± 0.0 μg/g dw (leaf surface)	HPLC-DAD, LC-MS/MS	[20]
		0.03–0.32 mg/g dw (twigs)<br>0.00–1.38 mg/g dw (hydrolysed extract)	HPLC-DAD	[76]
Ferulic and isoferulic acids	*M. bracteata*	0.11-0.24 mg/g dw (hydrolysed extract)	HPLC-DAD, LC-MS	[56]
	M. longifolia	0.72-1.38 mg/g dw (hydrolysed extract)	HPLC-DAD, LC-MS	[56]
Tannins				
Tamarixinin A	*M. bracteata*	0.14%		[61]
Long-chain fatty alcohols (alkanediols)				
series of hentriacontanediol isomers	*M. germanica*	3.5 per cm² of leaf surface area; 9% of the total wax mixture	GC-FID, GC-MS	[68]
homologous β-alkanediols	*M. germanica*	0.6 mg per cm² of leaf surface area; 2% of the total wax mixture	GC-FID, GC-MS	[68]
Other compounds				
Fatty acids	*M. bracteata*	in the fraction: linoleic acid 47.8%; oleic acid 33.4%; palmitic acid 10.6%	GLC	[70]
Essential oil	*M. germanica*	0.0183%	8-hour steam distillation; GC-MS (see Table S2)	[91]
Vitamin C	*M. bracteata*	10.1 mg/100 g (aerial parts)	titrimetric method	[70]
Vitamin E (tocopherol)	*M. bracteata*	3.5 mg/100 g (aerial parts)	fluorimetric method	[70]
Iron (Fe)	*M. bracteata*	3.38 %	AAS	[70]
Potassium (K)	*M. bracteata*	0.35 %	AAS	[70]

n.i. —non indicated

Table S2. Chemical constituents and relative contents of the essential oil from *M. germanica*. Data according to Zeng et al. 2014 [91].

No.	Compound	Relative content [%]
1.	Acetal	1.06
2.	Isopropyl formate	0.65
3.	(*s*)-(+)-Propylene glicerol[1]	0.88
4.	*p*-Xylene	0.04
5.	*m*-Xylene	4.17
6.	Monobutyl glycol ether	1.86
7.	3-Ethyloctane	2.86
8.	3-Ethyl-4-methylheptane	1.01
9.	Diisopentyl ether	0.36
10.	4,5-Dimethyloctane	2.55
11.	3,3,5-Trimethylheptane	0.08
12.	5-Methylheptan-3-one	0.01
13.	Phenol	0.78
14.	2-Ethylhexan-1-ol	0.46
15.	Phenylmethanol (benzyl alcohol)	0.82
16.	Phenylacetaldehyde	0.05
17.	*n*-Undecane	1.18
18.	Linalool	0.17
19.	(*Z*)-3,7-Dimethylocta-1,3,6-triene (beta-ocimene)	0.34
20.	Pelargonaldehyde (nonanal)	0.01
21.	Phenylethyl alcohol	1.01
22.	*n*-Butylbenzene	0.13
23.	Bornan-2-one (camphor)	0.22
24.	Naphthalene, molten	0.59
25.	Dodec-1-ene	0.09
26.	4,6,6-trimethylbicyclo[3.1.1]hept-3-en-2-one	0.05
27.	Benzothiazole	0.35
28.	Ethyl 5-methyl-1,2,3-thiadiazole-4-carboxylate	0.77
29.	1-Octadecene	0.15
30.	*trans*-2-Decenal	0.23
31.	*n*-Tridecane (standard material)	0.35
32.	2-Methylnaphthalene	1.07
33.	2-Methoxy-4-vinylphenol	0.04
34.	3,5-Diethylphenol	0.25
35.	*trans*-2,4-Decadienal	0.12
36.	2-*tert*-Butyl-4,6-dimethylphenol	0.31
37.	5-Allyl-2-methoxyphenol	0.01
38.	Eugenol	0.35
39.	Butanoic acid, butyl ester	2.23
40.	Decanoic acid	0.14
41.	Tetradecane	0.22
42.	2,6-Dimethylnaphthalene	2.59
43.	Vanillin	0. 34
44.	2,3-Dimethylnaphthalene	0.71
45.	Pentadecane	0.84

46.	1,6,7-Trimethylnaphthalene	5.43
47.	2,6-Bis(1,1-dimethylethyl)-4-ethylphenol	0.71
48.	Dodecanoic acid	0.02
49.	Hexadecane	1.06
50.	Cedrol	0. 36
51.	1,3-Dimethyl-5-(phenylmethyl)-benzene	0.04
52.	4-Methylhexadecane	0.85
53.	Butylated hydroxytoluene	0.14
54.	2-(3h)-Benzothiazolone	0.55
55.	1-Tetradecene	4.61
56.	Heptadecane	2.11
57.	Nonadecane	1.25
58.	2,2',5,5'-Tetramethylbiphenyl	0.45
59.	Octadecane	7.69
60.	Benzyl benzoate	4.10
61.	3,5-Di-*tert*-butyl-4-hydroxybenzaldehyde	0.21
62.	Tetradecanoic acid	0.28
63.	Anthracene	0.62
64.	Phenanthrene	0.43
65.	1-Nonadecene	0.59
66.	2,6,10,14-Tetramethylhexadecane	0.63
67.	Heneicosane	0.16
68.	Dibutyl phthalate	1.75
69.	1,2-Benzenedicarboxylic acid, butyl octyl ester	0.55
70.	Hexadecanoic acid, methyl ester	0.11
71.	*n*-Hexadecanoic acid	0.54
72.	1-Eicosene	1.21
73.	1-Chlorooctadecane	1.04
74.	1-Octadecanethiol	0.89
75.	9,12-Octadecadienoic acid (*Z*,*Z*) methyl ester	2.02
76.	Methyl *cis*-6-octadecenoate	2.41
71.	Octadecanoic acid, methyl ester	0.17
78.	12-Octadecadienoic acid	0.19
79.	(*E*)-9-Octadecenoic acid	0.23
80.	Octadecanoic acid	0.48
81.	Hexadecanamide	0.75
82.	Tetradecanamide	0.05
83.	Ethylene glycol monotetradecyl ether	0.14
84.	1-Iodo-2-methylundecane	0.04
85.	Oleic acid	0.18
86.	(*Z*)-9-Octadecenamide	0.25
87.	9-Methylnonadecane	0.02
88.	1,2-Benzenedicarboxylic acid, mono(2-ethylhexyl) ester	1.84
89.	3-Methyloctadecane	0.02
90.	6,10,14,18,22-Tetracosahexaene	0.15

[1] Inconsistency of the name with the given formula ($C_6H_{12}O_3$). The name "(*s*)-(+)-propylene glycerol" is used as a synonym of (*S*)-(+)-1,2-propanediol ($C_3H_8O_2$, m. w. 76.09 g/mol).
